# Supplementary material for: Clinical Surveillance vs. Anticoagulation For low-risk patiEnts with isolated SubSegmental Pulmonary Embolism: protocol for a multicentre randomised placebo-controlled non-inferiority trial (SAFE-SSPE)
Source: BMJ Open. 2020 Nov 19;10(11):e040151. doi: 10.1136/bmjopen-2020-040151 (PMC7678381; doi:10.1136/bmjopen-2020-040151)
Supplement: Supplementary data [file bmjopen-2020-040151supp001.pdf]

SUPPLEMENTARY FILES

Supplementary File 1: English language example of the informed consent form

SAFE-SSPE

Clinical surveillance vs. anticoagulation for low-risk patients with isolated subsegmental pulmonary embolism: a multicenter randomized placebo-controlled study

Original title of the study:
Clinical surveillance vs. anticoagulation for low-risk patients with isolated subsegmental pulmonary embolism: a multicenter randomized placebo-controlled non-inferiority trial (SAFE-SSPE)

This study is organized by:
Prof. Drahomir Aujesky, MD, MSc, Inselspital, Bern University Hospital

Dear Sir / Madam,

You have been diagnosed with a small blood clot in your pulmonary (lung) blood vessels, which specialists call a subsegmental pulmonary embolism. For this reason we would like to ask you if you would be willing to participate in a clinical study. This study is described below as follows: first a short summary to inform you about the study, followed by a more detailed description.

Summary

1	**Aim of the study**<br>The study explores the optimal management for adults with small blood clots in the pulmonary arteries, so called isolated subsegmental pulmonary embolism. It is currently unclear whether patients with isolated subsegmental pulmonary embolism really do benefit from the current treatment with blood thinners (anticoagulants). We are conducting this study to compare the effectiveness and safety of treating subsegmental pulmonary embolism with and without blood thinners.
2	**Selection of study participants**<br>You suffer from an isolated subsegmental pulmonary embolism and have a low-risk of complications. This is why we are providing you with this information leaflet.
3	**General Information about the study**<br>After exclusion of the simultaneous presence of a blood clot in the leg veins (deep vein thrombosis), patients with isolated subsegmental pulmonary embolism, will be 1:1 randomly divided with a probability of 50% each into one of two groups in this study: clinical surveillance plus placebo (a dummy-drug; a pill that does not contain any active medication) or clinical surveillance plus a blood thinner medication called rivaroxaban. Rivaroxaban is a tablet that acts as a blood thinner and is approved in Switzerland and other countries for the treatment of pulmonary embolism. The placebo/rivaroxaban group allocation is blinded, i.e neither you nor your physician nor the research team will know whether you are receiving placebo or rivaroxaban (double-blind). In an emergency however, this information can be made available at any time. The study will randomize 276 participants over a period of 4 years.<br>The treatment during the study will last 90 days. During this period the two treatment groups will be clinically monitored and compared in terms of risk of blood clots (thrombosis / embolism), bleeding, mortality, quality of life and the use of medical resources. This information will be gathered by means of three telephone calls at day 10, 30 and 90 after study enrollment.

4	**Procedure**<br>The first study visit will take place on-site immediately after enrollment and signing of the informed consent form (total duration approx. 1.5 hours) and will include the following:<br>- Ultrasound examination of the leg veins to exclude a blood clot (thrombosis). Patients with leg vein thrombosis (deep vein thrombosis) cannot participate in the study.<br>- Study participants who do not have deep vein thrombosis will be randomly assigned to either the group with clinical surveillance plus placebo or to the group with clinical surveillance plus blood thinner medication with rivaroxaban.<br>- Collection of baseline data: questions on symptoms, pre-existing medical conditions, medication; compilation of examination findings, laboratory results; questionnaire on quality of life (1)<br>- Provision of a patient diary containing study information and to record any symptoms and/or doctor's visits/ hospital stays.<br>- Provision of the corresponding study medication and start of treatment.<br><br>For the 90-day study phase, the study medication should be taken daily as prescribed. Further study contacts will be made by telephone:<br>- Telephone call 1: after approx. 10 days (duration approx. 10 minutes)<br>- Telephone call 2: after approx. 30 days (duration approx. 10 minutes)<br>- Questionnaire on quality of life (2): by mail or in-person after approx. 30 days (duration approx. 10-15 minutes)<br>- Telephone call 3: after approx. 90 days (duration approx. 10 minutes)<br>- Questionnaire on quality of life (3): by mail or in-person after approx. 90 days (duration approx. 10 minutes)<br>During the telephone calls you will be asked about possible recurrent embolism/ thrombosis and about bleeding as well as doctor's visits / hospitalizations, return to work or usual activities, quality of life and functional status, medications, and symptoms.
5	**Benefit**<br>We cannot guarantee you any personal benefit from participating in the study. Even if participation in the study does not directly benefit you, we do expect that the information we gain from the study will enable us to optimize the management for people with subsegmental pulmonary embolism in the future.
6	**Rights**<br>You decide voluntarily whether you would like to participate in the study or not. Your decision does not affect your medical treatment and you do not have to justify it.
7	**Responsibilities**<br>If you decide to participate in the study, we ask you to comply with certain requirements (e.g participation in the first study visit which includes an ultrasound examination of your leg veins, participation in telephone interviews).
8	**Risks**<br>A treatment strategy without blood thinning medication (placebo) for subsegmental pulmonary embolism could potentially lead to an increased risk of recurrent embolism or thrombosis. However, observational studies have shown that low-risk patients like yourself without blood thinners do not have an increased risk of thrombosis or embolism when compared to patients treated with blood thinners (anticoagulation). A treatment strategy with blood thinners increases the risk of bleeding.

Baumgartner C, *et al. BMJ Open* 2020; 10:e040151. doi: 10.1136/bmjopen-2020-040151

9	**Other treatment options**<br>Your physician will advise you on which other treatment options are available if you do not wish to participate in this study.
10	**Study results**<br>If we detect any findings that may affect your health during the study, we will inform you about these findings.
11	**Data confidentiality**<br>We comply with all legal data protection regulations, and all parties involved are bound by confidentiality. Your contact details will be forwarded to Inselspital, Bern University Hospital so that we can contact you for further follow-up during the study (see section 4. Procedure). The CT images of the lung may be sent unencrypted (i.e. with information about your name and date of birth) to the radiologists at the Bern University Hospital in Switzerland (coordinating study center) for subsequent central review. The rest of your personal and medical data will be protected and only used in an encrypted format. The encrypted data will only be used for other research projects if you give your separate consent for this.
12	**Withdrawal**<br>You can choose to no longer participate and withdraw from the study at any time. The data collected up until the point of study withdrawal will still be analyzed.
13	**Compensation**<br>You will not receive any financial compensation.
14	**Liability**<br>The insurance company Zürich Versicherungs-Gesellschaft AG will cover any damages that may arise within the scope of this study.
15	**Funding**<br>The study is financed by the Swiss National Science Foundation (SNSF). Bayer AG supports the study by providing the study medications (rivaroxaban and placebo).
16	**Contact person:**<br>Principal Investigator<br>Prof. Dr. med. Drahomir Aujesky<br>Universitätsklinik für Allgemeine Innere Medizin<br>Inselspital, Universitätsspital Bern<br>3010 Bern<br>The study team of Prof. Aujesky is available 24h a day at 031 632 77 83 (during office hours) or 079 737 26 55 (in emergencies outside of normal office hours).

Baumgartner C, *et al. BMJ Open* 2020; 10:e040151. doi: 10.1136/bmjopen-2020-040151

Detailed information

1. Aim of the study

The aim of this study is to investigate the efficacy and safety of a management strategy with and without blood thinning medication for patients with small blood clots in their pulmonary arteries, so-called isolated subsegmental pulmonary embolism.

2. Selection of study participants

All persons aged 18 years and over who suffer from an isolated subsegmental pulmonary embolism, who do not have a simultaneous blood clot in the deep veins of the leg (deep vein thrombosis), and who are at low risk of complications can participate in this study.

However, the following individuals cannot participate in the study: those with simultaneous deep vein thrombosis, previous unprovoked venous thrombosis or pulmonary embolism (venous thromboembolism) i.e. a venous thromboembolism which occurred without a specific trigger, active cancer, unstable cardiovascular or respiratory function, active bleeding or those who have a very high bleeding risk, persons who take medications that interact with rivaroxaban, those with severe kidney or liver disease, an allergy towards rivaroxaban, pregnant or breastfeeding women, as well as persons who have an indication for a strong (therapeutic dose) blood thinning medication because of another concomitant disease, persons who already received therapeutic-dose blood thinning medication for >72 hours before study enrollment, or who were hospitalized for more than 72 hours before the diagnosis of a subsegmental pulmonary embolism was made.

3. General Information

Pulmonary emboli are blood clots in the pulmonary arteries and cover a broad spectrum ranging from large to very small (so-called subsegmental) emboli. The vast majority of pulmonary emboli, regardless of their size, are treated with blood thinners which are associated with a risk of bleeding. However, the risk-benefit ratio of blood thinners in treating subsegmental pulmonary embolism is unclear.

In this international study with study centers in Switzerland, the Netherlands and Canada, the effectiveness and safety of a treatment strategy with or without blood thinning medication for patients with subsegmental pulmonary embolism will therefore be compared. Patients with isolated subsegmental pulmonary embolism but without simultaneous deep vein thrombosis will, after appropriate explanation and informed consent, be randomized 1:1 with a probability of 50% each into one of two groups: clinical surveillance plus placebo or clinical surveillance plus a blood thinning medication called rivaroxaban. The group allocation is blinded, i.e. neither the patient nor the physician (including the study team) are aware of the group allocation (double-blind principle). Patients who have already agreed to participate in the study but who are later diagnosed with a deep vein thrombosis, cannot continue to participate in the study. The data collected up until the diagnosis of the deep vein thrombosis will be kept confidential and stored in our encrypted database.

The study medication rivaroxaban is a blood thinner in tablet form, which is approved for use in Switzerland, the EU, and North America for the treatment of pulmonary embolism. During the first 21 days, rivaroxaban will be taken at a dose of 15mg twice daily; from day 22 the daily dose will be changed to a 20mg tablet once daily. For participants randomized to the placebo group the same treatment regimen applies (i.e. 2 tablets per day for the first 21 days, followed by 1 tablet per day thereafter). The total duration of treatment during the study is 90 days. During this study period, both treatment groups will be interviewed three times via telephone calls on days 10, 30 and 90 after study enrollment in order to carry out clinical surveillance and at the end of the study to compare the two treatment groups with regards to the risk of thrombosis/emboli, bleeding, mortality, quality of life (PEmb-QoL questionnaire), functional status, as well as the use of medical resources.

Large pulmonary emboli are usually treated with blood thinners for at least 3 months in order to reduce the risk of thrombosis / pulmonary embolism recurrence. As a side effect, however, the blood thinning medication increases the risk of bleeding, and 1-5% of patients within 3 months of starting therapy experience severe bleeding, e.g. gastrointestinal bleeding, or less frequently a brain hemorrhage. For persons with subsegmental pulmonary embolism and a low risk of complications there is no standard treatment currently available, since it is not clear from today's scientific knowledge if treatment with or without a blood thinner is better. Certain physicians prescribe blood thinners in these cases, whilst other physicians avoid using them. If our study shows that treatment with blood thinners is no better at preventing venous thrombosis / pulmonary embolism compared to a management strategy without blood thinners, then unnecessary treatment with blood thinners and the associated bleeding risk could be reduced in low-risk patients with isolated subsegmental pulmonary embolism.
The study will randomize 276 patients over 4 years.

We are conducting this study in accordance with Swiss laws and regulations. We also comply with all internationally recognized guidelines. The responsible ethics commission and EudraCT have reviewed and approved the study.
A description of this study can also be found on the website of the Bundesamt für Gesundheit (Federal Office of Public Health): www.kofam.ch (registration number SNCTP000003905).

4. Procedure

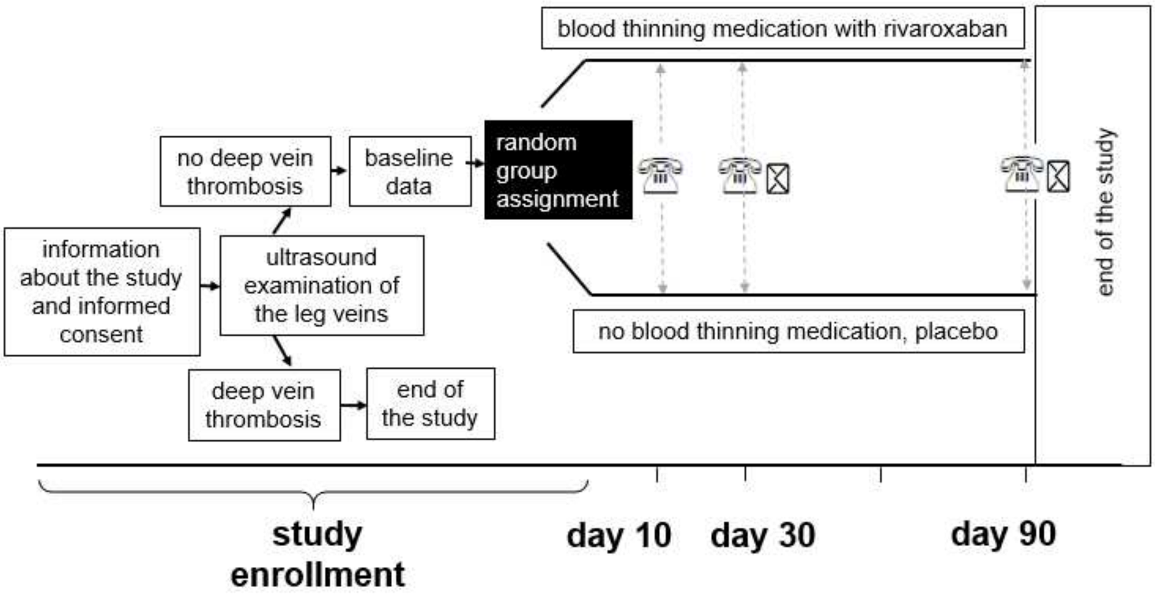

After you have been informed about the study and received this study information document, you will have time to ask any questions you may have about the study, the study information, or the informed consent form. If you wish to participate in the study, you must confirm this in writing by signing the consent form. You can keep this study information and you will receive a copy of the signed consent form.

In women of childbearing age, a pregnancy test (urine or blood test) will be performed prior to inclusion in the study, if a test has not already been performed during routine clinical practice.

Baumgartner C, *et al. BMJ Open* 2020; 10:e040151. doi: 10.1136/bmjopen-2020-040151

At the time of study enrollment a study visit will take place on-site immediately after signing the consent form. This study visit will include the following:

Collection of contact details: study personnel will ask you about your contact information (so that we can contact you for further monitoring during the study), contact details of family members/next of kin and of your general practitioner.

Ultrasound examination of the leg veins: a systematic and thorough ultrasound examination of the veins in both legs will be carried out to rule out a deep vein thrombosis, i.e. a blood clot causing a blockage in a vein. The technical term for the ultrasound procedure used is called compression ultrasonography. The veins in the thigh and lower leg will be examined for their compressibility. If a thrombosis is present, the corresponding section of vein is barely or not at all compressible. This is a non-invasive and painless procedure without any side effects. This examination will be performed on-site and with the patient lying down (if necessary, parts of the examination may also be performed in the seated position) and will be performed by a physician experienced in ultrasound examinations; the ultrasound examination takes approx. 15-20 minutes per leg, i.e. a total of about 30-40 minutes. This examination is performed because the presence of a deep vein thrombosis is an exclusion criterion for the present study.
For patients who are diagnosed with a deep vein thrombosis during the leg ultrasound examination, the study ends here and the further management plan will be determined by the treating physician.

Data collection at baseline: if a deep vein thrombosis has been excluded, study personnel will ask you questions about demographic data, risk factors for venous thromboembolism (i.e. pulmonary embolism or thrombosis), possible symptoms of venous thromboembolism, other diseases, and the medication you are taking. We will collect information from the treating physician's records on your previous illnesses, examination findings, laboratory results and results from the computer tomography examination (CT scan) through which the subsegmental pulmonary embolism was originally diagnosed. We will also ask you to complete a quality of life questionnaire regarding your diagnosis of subsegmental pulmonary embolism. Completing the questionnaire will take about 10-15 minutes. The collection of baseline data will be carried out on-site and takes approx. 30 minutes.

Randomization and delivery of the study medication and patient diary: We will provide you with the study medication, selected for you at random (randomization), i.e. rivaroxaban or placebo; you should start taking the medication immediately. In addition, we will also provide you with a patient diary which contains information about the study, the study medication and contact details of the Principal Investigator and his study team. Furthermore, we ask you to record in the diary the date of symptom resolution and the date of return to work or your usual housekeeping/leisure activities. You should also note in your diary if you suffer any bleeding or thrombosis / pulmonary embolism recurrence, if you need to visit a physician (general practitioner, specialist or emergency department) or if you are hospitalized.

The remaining study contacts will take place via telephone call and no further study visits are necessary. The remaining study contacts will take place as follows:

Telephone call: approx. 10 days (7-12 days) after study enrollment.

Telephone call: approx. 30 days (28-35 days) after study enrollment.

Questionnaire on quality of life (2): approx. 30 days (28-35 days) after study enrollment, by mail or in-person.

Telephone call: approx. 90 days (88-95 days) after study enrollment.

Questionnaire on quality of life (3): approx. 90 days (88-95 days) after study enrollment, by mail or in-person.

During the follow-up phone calls or visits you will be personally interviewed by study personnel from the Inselspital, Bern University Hospital (the institution responsible for the study in Switzerland). In case we cannot reach you personally after three attempts, we will contact your general practitioner/treating physician or other health care professional involved in your care (e.g. health care personnel from a retirement home, nursing home, or rehabilitation center) and/or the contact person you identified as your next of kin (relative, other close person). We expect the telephone interviews to take about 10 minutes each. During these telephone calls, the study coordinator will ask you about any new thrombosis / embolism and bleeding. You will also be asked about any doctor's visits or any hospital stays, the date of return to work or your usual daily activities, your functional status in everyday life, your medications and about any possible symptoms or signs compatible with thrombosis or embolism, or bleeding.
Approx. one and three months after study enrollment, we will send you another quality of life questionnaire by mail and ask you to complete it and return it to us in a pre-paid envelope.

Returning the study medication and patient diary: Once treatment is complete at the end of the study, we will ask you to please return the pill bottles containing the remaining tablets, as well as the patient diary to us in a pre-paid envelope.

Your general practitioner will be informed at the beginning of the study about your participation. It is also possible that we may contact your general practitioner / treating physician or another health care professional (e.g. hospital doctors, health care professionals from the emergency department, rehabilitation, nursing home, specialist doctors) to collect healthcare data regarding your hospital stays and any newly diagnosed illnesses. This is in order to obtain accurate medical information about your health and about the effects or side effects of treatment.

We may have to prematurely exclude you from the study. This may happen if you end up needing a long-term blood thinner for a reason other than for treatment of your subsegmental pulmonary embolism, if you end up needing a long-term medication that interacts with the study medication, if you develop recurrent pulmonary embolism or thrombosis, if you experience severe bleeding, or if you become pregnant. In this case we will inform you about the end of your study participation, compile the information collected during the previous telephone calls (see above), and ask you to please return all remaining medication (rivaroxaban or placebo) that we have given you.

5. Benefit

We cannot guarantee you any personal benefit from participating in the study. Even if participation in the study does not directly help you, we expect the information we gain from the study will contribute to improving the management of patients with subsegmental pulmonary embolism in the future. However, it is possible that by participating in the study and having the associated follow-up telephone calls, any complications you may have can be identified more quickly and communicated to your treating physician.

6. Rights

Your participation is voluntary. If you do not want to participate or later choose to withdraw from the study, tyou do not have to justify this if you do not want to. Your medical treatment/care is guaranteed regardless of your decision. You may ask questions about participation in the study at any time. Please contact the person named at the end of this information sheet.

Baumgartner C, *et al. BMJ Open* 2020; 10:e040151. doi: 10.1136/bmjopen-2020-040151

7. Responsibilities

As a participant, it is necessary that you

- adhere to the necessary guidelines and requirements of the study, such as participation in the first study visit including ultrasound examination of the leg veins, daily intake of the study medication as prescribed, participation in telephone follow-up calls (or in-person visits), keeping a patient diary, completing and returning the questionnaires on quality of life as mentioned above, and returning the pill bottles at the end of the study.
- inform the study team about new symptoms, new ailments and changes in your condition that may be related to a new thrombosis or embolism (e.g. new or increasing shortness of breath, new or worsening cough, new chest pain, coughing up blood, one-sided pain or swelling in your leg), as well as in the event of prolonged or severe bleeding or signs of possible bleeding (unusual weakness, tiredness, paleness, dizziness, headache, unexplained swelling or chest pain).
- inform the study team about simultaneous treatment and therapy from another physician and about the medication that you take.

8. Risks and burden for the participants

A treatment strategy without blood thinners for subsegmental pulmonary embolism could potentially lead to an increased risk of recurrence of pulmonary embolism or deep vein thrombosis. However, observational studies have shown that in low-risk patients like yourself there was no increased risk of recurrent pulmonary embolism or thrombosis in people without blood thinning treatment compared to those who took anticoagulants. A treatment strategy with a blood thinner leads to an increased risk of bleeding.

For women who may become pregnant

Based on animal studies, we know that rivaroxaban does not cause harm to unborn animals. Nevertheless, the effects of the study medication on the unborn child have not yet been adequately investigated. We do however know from animal studies that rivaroxaban during pregnancy is harmful and can lead to maternal bleeding complications. For this reason, female study participants must use a reliable contraceptive method during the study (e.g. hormonal methods such as the pill or coil / intrauterine system).

Should you become pregnant during the study, you must inform the study team immediately and stop taking the study medication. The study physician will discuss the next steps with you. If you are breastfeeding, you are excluded from participation.

9. Other treatment options

You do not have to participate in this study. If you do not participate, your treating physician will work with you to decide whether to start treatment with a blood thinner for at least 3 months (depending on the blood thinner, additional blood thinning injections may be necessary during the first few days), or whether to choose a management strategy without a blood thinning medication.

10. Results from the study

During the trial, the study physician will inform you about any new findings that may affect the benefit of the trial or your safety and thus your consent to participate in the trial. You will receive the information orally and in writing.

11. Confidentiality of data

Your personal and medical data will be collected for this study. Very few professionals will see your unencrypted data, and this will only occur for the purpose of carrying out tasks related to the study. Data collected for study purposes will be encrypted. Encryption means that all data related to you that could identify you (name, date of birth) is deleted and replaced by a number-key. The numbered-key list and your non-encrypted contact details can be accessed by the study team at

Baumgartner C, *et al. BMJ Open* 2020; 10:e040151. doi: 10.1136/bmjopen-2020-040151

Inselspital, Bern University Hospital (institution responsible for conducting the study in the Switzerland), so that you and, if necessary, the contact person you identified or your treating physician/medical staff can be contacted for further follow-up during the study (e.g. telephone calls, questionnaires etc., see section 4. Procedure).
All other professionals from Switzerland, the Netherlands, and Canada who are involved in our study only have access to your encrypted data for research purposes (analysis). Those who do not know the number-key cannot therefore trace the data back to you. In the case of a publication, the aggregate data also cannot be traced back to an individual person such as yourself. Your name will never appear on the internet or in a publication. Sometimes for publication in a journal there is a requirement to pass on the individual data (so-called raw data). If individual data must be transmitted, then the data is always encrypted and cannot be traced back individually to you. All persons who have access to your data within the scope of the study are bound by the principles of confidentiality. The requirements for data protection will be observed and you as a study participant have the right to view your data at any time.

Data is collected electronically using a database for research purposes (EDC System secuTrial at the Clinical Trial Unit, Mittelstrasse 43, 3012 Bern, Bern University Hospital). Data will be stored for at least 10 years.

It is possible that your encrypted data may be reused for other investigations at a later date or sent to another database in Switzerland or abroad for as yet undefined further use. This other database must comply with the same standards as the database for this study. For this further use of your data, we ask you to sign an additional consent form at the very end of this document.

This study may be reviewed by the responsible ethics committee, the medication authority Swissmedic or by the institution that initiated the study (Bern University Hospital). The Principal Investigator may have to disclose your personal and medical data for such checks. It may also be the case that a representative of the insurance company might also inspect your data in exceptional cases in the event of damage. Bayer AG who provides the study medication, may receive access to your encrypted data (i.e. without any identifying information such as name or date of birth) for monitoring purposes. All persons must maintain absolute confidentiality.

As mentioned above, it is possible that the contact person you identified, your general practitioner/treating physician or other healthcare professionals that treat you (e.g. in retirement homes, nursing homes, rehabilitation centers) may be contacted to provide us with information about your state of health.

Subsequent analyses of the CT images by radiologists at Bern University Hospital

In order to guarantee a uniform diagnosis of an isolated subsegmental pulmonary embolism among all study participants, the CT (computer tomography) images of the lung will be forwarded unencrypted i.e. with details of your name and date of birth, to the radiologists at Bern University Hospital for central review:

Inselspital Bern
University Institute for Diagnostic, Interventional, and Pediatric Radiology (Universitätsinstitut für Diagnostische, Interventionelle und Pädiatrische Radiologie)
Freiburgstrasse 10
CH-3010 Bern

CT images will be stored in a digital radiological images archiving system for at least 10 years.

12. Withdrawal
You can stop and withdraw from the study at any time if you wish. The data collected up to that point will still be evaluated in an encrypted format, otherwise the entire project will lose its value. It is not possible to completely anonymize your data in case of withdrawal, i.e. the data will remain encrypted (coded), i.e. without any patient-identifiable information. Please check whether you agree to this before you participate in the study.

13. Compensation for participants
If you participate in this study, you will not receive any financial compensation.
You or your health insurance company will not incur any costs as a result of study participation.

14. Liability
The institution (Inselspital, Bern University Hospital), which is responsible for carrying out the study in Switzerland, is liable for any damage that you might incur in connection with the research activities (e.g. investigations/treatment). The conditions and procedure for this is regulated by law. The Inselspital, Bern University Hospital has therefore taken out insurance with Zürich Versicherungs-Gesellschaft AG to cover liability in the event of possible damages.
The same liability rules apply as would to a treatment outside the scope of the present study. Namely, in the event of damages attributable to rivaroxaban (an approved medical substance used in accordance with medical standards), or damages which occur as part of the use of a placebo, or damages which would also have occurred in the case of usual care, the same liability rules apply as for a treatment outside of the study.
If you suffer any damages, please contact your study physician or the insurance company mentioned above.

15. Financing of the study
The study is fully funded by the Swiss National Science Foundation (SNSF). Bayer AG supports the study by providing the study medication.

16. Contact person
If you have any questions, uncertainties or emergencies that arise during or after the study, you can contact the Principal Investigator at any time:

Principal Investigator:
Prof. Dr. med. Drahomir Aujesky
Universitätsklinik für Allgemeine Innere Medizin
Inselspital, Universitätsspital Bern
Freiburgstrasse
3010 Bern

The study team of Prof. Aujesky can be reached 24h a day at: 031 632 77 81 or
079 737 26 55 (in emergencies outside office hours)
Email: safe-sspe@insel.ch

17. Glossary (technical terms explained)

- What does “placebo” mean?
  Some people who receive a medication do not actually get better from the medication itself, but rather from the care and attention of the physician. This can be seen by the fact that some people can feel better, even if they get a so-called sham medication or dummy-drug. This sham medication looks like a real medication and is also packaged identically. However, there is in fact no active ingredient in this sham medication. It is called a "placebo".
  Sometimes one treats some of the participants in a clinical trial with the real medication (which contains the active ingredient) and the other participants with a placebo (without the active

ingredient). It is then easier to make a comparison and assess how well the medication actually works or whether the improvement observed only occurred because people received care and attention. Sometimes the improvement simply corresponds with the natural course of the disease.

- What does “randomized” mean?
  Many studies compare two or more different types of treatment. For example, one can compare an active “real” medication with a placebo. One then forms two groups of participants, one group receives the real medication and the other group the placebo. "Randomization" then means that participants are randomly assigned to different groups. Therefore, it is a coincidence whether the participant receives the real medication or the placebo.

- What does "double-blind" mean?
  To “blind” a study serves to obtain better and more accurate results.
  "Double-blind" is therefore a study where neither the participants nor the researchers know whether a study participant is receiving the real medication or the placebo. Only the independent person who assigned the group allocation knows who receives what. When the test is over, the "blinding" will be removed. In an emergency, the "blinding" can also be removed earlier.
  A person who knows that he or she is receiving the real medication and not the placebo, will pay very different attention to symptoms in his/her body than someone who knows that he/she is only receiving the placebo. This can lead people who receive the real medication to overestimate the effect of the medication compared to those who only receive the placebo.

Baumgartner C, *et al. BMJ Open* 2020; 10:e040151. doi: 10.1136/bmjopen-2020-040151

Informed consent

Written declaration of consent for participation in a study project
Please read this form carefully. Please ask if you do not understand something or would like to know more. Your written consent is required for participation.

**IRB-Number (after submission):**	2019-02297
**Title of the study (scientific and layperson language)**:	Clinical surveillance or blood thinner for low-risk patients with isolated subsegmental pulmonary embolism: a multicenter randomized placebo-controlled study
**Responsible institution (Sponsor with address)**:	Inselspital, Universitätsspital Bern<br>Prof. Dr. med. Drahomir Aujesky<br>Freiburgstrasse<br>3010 Bern
**Site where study will be conducted**:	Inselspital, Universitätsspital Bern<br>Universitätsklinik für Notfallmedizin<br>Freiburgstrasse<br>3010 Bern
**Responsible investigator at study site:**<br>Surname und first name in block capital letters:	Prof. Dr. med. Drahomir Aujesky
**Study participant:**<br>Surname and first name in block capital letters:<br>Date of birth:	☐ female ☐ male

- I have been informed, both verbally and in writing, by the study team member signing below about the purpose of the study, the course of the study with clinical surveillance and rivaroxaban (blood thinner) or placebo, and about possible advantages and disadvantages as well as possible risks.
- I am voluntarily participating in this study and acknowledge the content of the written patient information provided. I have been given enough time to make my decision.
- My questions regarding participation in this study have been answered. I will keep the written patient information provided and will receive a copy of my written informed consent form.
- I have been informed about possible other treatments and treatment procedures.
- I agree to my general practitioner being informed about my participation in the study.
- I agree to allow the sponsor-representative's medical experts, the responsible ethics committee and the medication authority Swissmedic to inspect my unencrypted data for testing and control purposes, but in keeping with strict confidentiality regulations.
- I will be informed in the event of study findings that directly affect my health.
- I know that my personal contact details will available to the study team at Inselspital, Bern University Hospital (responsible study center in the Switzerland), so that the study team can contact me and if necessary the contact person I identified, or contact my treating physician/medical staff for further follow-up during the study period. Furthermore, I also authorize my treating physician(s) or the healthcare professionals from other institutions that treat me (e.g. heath care professionals from retirement homes, nursing homes, rehabilitation centers) to

provide the study team at Inselspital with any of my health-related data that may be relevant to this study. This information will be used exclusively for study purposes only.

- I know that my health-related and personal data can only be passed on in an encrypted format for research purposes for this study (including transfer abroad).
- I agree to the CT (computer tomography) images of my lungs, which were used to diagnose my subsegmental pulmonary embolism, being forwarded unencrypted to the radiologists at Bern University Hospital for central review (i.e. with name and date of birth specified).
- If during the ultrasound examination of my leg veins (which is performed on all study participants during the initial study visit), I am diagnosed with a deep vein thrombosis, I acknowledge that I cannot continue to participate in the study. The data collected up until this point will be kept confidential and stored in an encrypted form.
- I can withdraw from the study at any time and without giving a reason. My further medical treatment is always guaranteed and independent of whether I participate in the study or not. The data and samples collected up until the point of withdrawal will be used for the analyses in the study.
- I have been informed that an insurance company will cover damages that are attributable to this study.
- I am aware that the responsibilities stated in this study participant information sheet must be complied with. In the interest of my own health, the Principal Investigator may exclude me from the study at any time.

Place, date	Signature of participant

Confirmation by the local investigator: I hereby confirm that I have explained the nature, significance and scope of the study to this participant. I confirm that I will fulfil all my obligations regarding this study in accordance with the relevant laws. If at any time during the study, I become aware of any aspect that may affect the participant's willingness to participate in the study, I will inform the participant immediately.

Place, date	Surname and first name of the local investigator in block capital letters<br><br>Signature of the local investigator

Baumgartner C, *et al. BMJ Open* 2020; 10:e040151. doi: 10.1136/bmjopen-2020-040151

Declaration of consent to further use of data from this study in an encrypted form

**Participant:**<br>Surname and first name in block capital letters:<br>Date of birth:	☐ female ☐ male

I allow my data from this study to be re-used for medical research. This means that the data may be stored in a database and used for future, as yet undefined, research projects for an unlimited period of time. This consent is indefinite.

My decision is voluntary and I can revoke this decision at any time. If I withdraw from the study, my data will be anonymized. In the event of my decision to withdraw, I will inform the investigator and do not have to justify my decision.

I understand that the data is encrypted and the security key to decode it will be kept safe and secure. The data can be sent abroad or inland to other databases for analysis, if the same standards as in Switzerland are complied with. All legal requirements regarding data protection are complied with.

Normally, all data will be analyzed in its entirety and the results published in a summary form. If a result that is important to my health should arise, then it is possible that I will be contacted by a member of the study team.

If results from the data are commercialized, I have no claim to a share of the commercial profits.

Place, date	Signature of the participant

Confirmation by the local investigator: I hereby confirm that I have explained to this participant the nature, significance and implications of the further use of his/her data.

Place, date	Surname and first name of the informing local investigator in block capital letters<br><br>Signature of the local investigator

Baumgartner C, *et al. BMJ Open* 2020; 10:e040151. doi: 10.1136/bmjopen-2020-040151

Supplementary File 2: Case Report Form (CRF) for follow-up interview 10 days after randomization

Patient ID

Follow up interview

(04.05.2020 - 13:25:25 (CEST))

**Follow-up interview number**	○ 1 ○ 2 ○ 3
**Date of follow-up interview phone call**	☐ dd.mm.yyyy
**Follow up interview completed**	○ yes ○ no
**Please indicate the reason why follow-up call wasn't completed**	○ lost to follow-up ○ withdrawal from study ○ patient died
**Please specify the reason for withdrawal**	
**Contact(s) made to obtain follow-up information**	
**Patient**	☐
**Designated contact person (family or friend)**	☐
**Primary care physician**	☐
**Other**	☐
**Please specify "other"**	
**Agreement for consecutive follow up**	○ patient agrees to be contacted for final assessment at 90 days<br>○ patient agrees for passive follow-up (i.e. information collected from hospital medical records and/or primary care physician)
**Planned date of next follow-up call**	☐ dd.mm.yyyy

Follow up interview

Patient ID

Medical outcomes
(04.05.2020 - 13:25:25 (CEST))

VTE RECURRENCE

VTE recurrence ○ Yes ○ No

Interruption of study medication and unblinding is indicated if VTE is objectively confirmed

Date of diagnosis [ ] dd.mm.yyyy

Confirmatory diagnostic exam:

- **CTPA** ☐
- **ventilation-perfusion scintigraphy** ☐
- **pulmonary angiography** ☐
- **venous ultrasound** ☐
- **IV phlebography** ☐
- **CT or MRI for thrombosis of the iliac vein or vena cava inferior** ☐
- **echocardiography** ☐

Type of recurrent VTE ○ PE ○ DVT

Localization of PE
○ subsegmental PE
○ more proximal PE

Localization of DVT
○ proximal DVT (V.cava, iliac vein, common or superfician femoral vein, popliteal vein)
○ distal DVT (posterior or anterior tibial vein, peroneal vein, gastrocnemial vein, soleal vein)

Stop of study medication following recurrent VTE ○ Yes ○ No

Enter the date of stopping the study medication [ ] dd.mm.yyyy

CLINICALLY SIGNIFICANT BLEEDING

Clinically significant bleeding ○ Yes ○ No

Clinically significant bleeding 1

Date of bleeding episode [ ] dd.mm.yyyy

Type of physician contact(s)

- **phone call to physician** ☐
- **office visit** ☐
- **ED visit** ☐
- **hospitalization** ☐
- **none** ☐

Treatment with antiplatelet agent or NSAID at the time of bleeding ○ Yes ○ No

- **Aspirin** ☐
- **clopidogrel** ☐

Medical Outcomes

Patient ID

- prasugrel ☐
- ticagrelor ☐
- NSAIDs ☐

Packed red cell transfusions ○ Yes ○ No

Indicate the number of units ☐

Decrease in hemoglobin of at least 20 g/L within 7 days after the bleeding event ○ Yes ○ No ○ Unknown

Site of bleeding

- epistaxis ☐
- cutaneous ☐
- subcutaneous ☐
- intramuscular with compartment syndrome ☐
- intramuscular without compartment syndrome ☐
- gastrointestinal [melena, hematochezia, hematemesis] ☐
- intracranial ☐
- retroperitoneal ☐
- pulmonary ☐
- intraarticular ☐
- intraspinal ☐
- intraocular ☐
- pericardial ☐
- hematuria ☐
- vaginal ☐
- other ☐
- other, please specify ☐

Diagnostic test(s) performed to localize bleeding source

- MRI ☐
- CT ☐
- ultrasound ☐
- upper endoscopy ☐
- lower endoscopy ☐
- capsule endoscopy ☐
- bronchoscopy ☐

Medical Outcomes

Patient ID

cystoscopy ☐
arteriography ☐
erythrocyte scintigraphy ☐
diagnostic surgery ☐
other ☐
other, please specify

Treatment(s) of bleeding:

fresh frozen plasma ☐
recombinant factor VIIa concentrate ☐
prothrombin complex [factor II, VII, IX, X] ☐
factor Xa inhibitor-specific antidote [andexanet alpha] ☐
tranexamic acid ☐
invasive or surgical hemostasis procedures ☐
other ☐
other, please specify

Interruption of study medication following bleeding ○ Yes ○ No
duration of interruption ○ definite ○ temporary
date of interruption dd.mm.yyyy
date of restart dd.mm.yyyy

Clinical impact of overt bleeding:

temporary or permanent cessation of the study drug ○ Yes ○ No
overt bleeding associated with relevant pain ○ Yes ○ No
impairment of activities of daily life ○ Yes ○ No

More

Medical Outcomes

Patient ID

Medical resource utilization

(04.05.2020 - 13:25:25 (CEST))

Initial hospitalization ongoing ◯ Yes ◯ No

Date of discharge from initial hospitalization ____ dd.mm.yyyy hh:mm

Subsequent hospitalizations ◯ Yes ◯ No

Subsequent hospitalizations 1

Admission date ____ dd.mm.yyyy

Discharge date ____ dd.mm.yyyy

Name of hospital ____

Symptoms or signs leading to hospitalization

- **dyspnea** ☐
- **cough** ☐
- **chest pain** ☐
- **bloody cough [hemoptysis]** ☐
- **syncope** ☐
- **pleural effusion** ☐
- **leg pain** ☐
- **leg swelling** ☐
- **bleeding** ☐
- **other** ☐
  - **please specify** ____

Primary discharge diagnosis ____

Please specify ____

Discharge location ____

Other, please specify ____

Notes ____

More

Subsequent emergency department visits ◯ Yes ◯ No

Subsequent emergency department visits 1

Presentation date ____ dd.mm.yyyy

Specify hospital name ____

Medical Resource Utilization

Patient ID

Symptoms or signs leading to ED visit

- dyspnea ☐
- cough ☐
- chest pain ☐
- bloody cough [hemoptysis] ☐
- syncope ☐
- pleural effusion ☐
- leg pain ☐
- leg swelling ☐
- bleeding ☐
- other ☐
  - please specify

Primary diagnosis at the emergency department

Please specify

Notes

More

Subsequent physician outpatient visits ○ Yes ○ No

Subsequent physician outpatient visits 1

Visit date dd.mm.yyyy

Reason for visit

- dyspnea ☐
- cough ☐
- chest pain ☐
- bloody cough [hemoptysis] ☐
- pleural effusion ☐
- leg pain ☐
- leg swelling ☐
- bleeding ☐
- other ☐

Please specify

file:///C/Data/Temp/Medical_resource_utilization.html[05/05/2020 15:37:48]

Patient ID

**Physician diagnosis**	
**Notes**	

More

Medical Resource Utilization

Patient ID

Time to symptom resolution

(04.05.2020 - 13:25:25 (CEST))

**Resolution of dyspnea**	○ Yes ○ No ○ Symptom has not been present
**Date of symptom resolution**	[ ] dd.mm.yyyy
**Resolution of cough**	○ Yes ○ No ○ Symptom has not been present
**Date of symptom resolution**	[ ] dd.mm.yyyy
**Resolution of chest pain**	○ Yes ○ No ○ Symptom has not been present
**Date of symptom resolution**	[ ] dd.mm.yyyy
**Resolution of bloody cough**	○ Yes ○ No ○ Symptom has not been present
**Date of symptom resolution**	[ ] dd.mm.yyyy
**Returned to work or usual activities**	○ Yes ○ No
**date of returning to work or usual activities**	[ ] dd.mm.yyyy

Symptom Resolution and Return to Work

Patient ID

Discontinuation or modification of study medication

(04.05.2020 - 13:25:25 (CEST))

**Was another anticoagulant agent started during study period?**	○ Yes ○ No
**Type of anticoagulant**	
**Please specify**	
**Duration of anticoagulant treatment**	○ extended ○ temporary
**Date of starting the anticoagulant**	dd.mm.yyyy
**Date of stopping anticoagulant**	dd.mm.yyyy
**Dose of anticoagulant**	○ therapeutic ○ prophylactic
**Reason for starting another anticoagulant**	
**Atrial fibrillation / atrial flutter with a CHADS2 score of ≥1 or CHA2DS2-VASc ≥2**	☐
**mechanical heart valve**	☐
**upper or lower extremity DVT**	☐
**pulmonary embolism**	☐
**intraabdominal thrombosis (e.g. portal vein thrombosis)**	☐
**intracranial vein thrombosis**	☐
**VTE prophylaxis**	☐
**bridging for intervention/procedure**	☐
**other**	☐
**please specify**	
**Discontinuation of study medication during follow-up**	○ Yes ○ No
**Date of discontinuation**	dd.mm.yyyy
**<u>Reason for discontinuation:</u>**	
**need for prophylactic anticoagulation**	☐
**new indication for permanent therapeutic anticoagulation**	☐
**specify indication:**	
**new diagnosis of atrial fibrillation/flutter with a CHADS2 score of ≥1 or CHA2DS2-VASc ≥2**	☐
**mechanical heart valve**	☐
**upper or lower extremity DVT**	☐

Discontinuation or Modification of Study Medication

Patient ID

- pulmonary embolism ☐
- intraabdominal thrombosis (e.g. portal vein thrombosis) ☐
- intracranial vein thrombosis ☐
- other ☐
- please specify

bleeding ☐

elective invasive procedure or surgical intervention requiring interruption of anticoagulation ☐

please specify procedure/ intervention

emergency invasive procedure or surgical intervention requiring interruption of anticoagulation ☐

please specify procedure/ intervention

was an emergency anticoagulation reversal done prior to the emergency procedure? ○ Yes ○ No

indicate agent/method used for anticoagulation reversal:

- fresh frozen plasma ☐
- recombinant factor VIIa concentrate ☐
- prothrombin complex [factor II, VII, IX, X] ☐
- factor Xa inhibitor-specific antidote [andexanet alpha] ☐
- tranexamic acid ☐
- invasive or surgical hemostasis procedures) ☐

Discontinuation or Modification of Study Medication

Patient ID

**new diagnosis of pregnancy**	☐
**new use of medications interacting with rivaroxaban**	☐
**strong CYP3A4 inhibitors or inducers**	☐
**saquinavir**	☐
**indinavir**	☐
**ritonavir**	☐
**nelfinavir**	☐
**atazanavir**	☐
**fosamprenavir**	☐
**tipranavir**	☐
**darunavir**	☐
**ketoconazole**	☐
**itraconazole**	☐
**voriconazole**	☐
**posaconazole**	☐
**rifampicin**	☐
**rifabutin**	☐
**rifapentin**	☐
**phenytoin**	☐
**phenobarbital**	☐
**primidone**	☐
**carbamazepine**	☐
**St. John's Wort**	☐
**specify indication**	
**HIV**	☐
**fungal infection**	☐
**epilepsy**	☐
**depression**	☐
**other**	☐
**please specify**	

**new dual antiplatelet therapy (aspirin plus P2Y12-inhibitor)**	☐
**Please specify**	○ Aspirin plus clopidogrel<br>○ aspirin plus ticagrelor<br>○ Aspirin plus prasugrel<br>○ Other

Discontinuation or Modification of Study Medication

Patient ID

Please specify indication [ ]

new GP IIb/IIIa inhibitors ☐

specify medication
○ abciximab
○ eptifibatide
○ tirofiban

specify indication [ ]

patient withdraws from study participation ☐

Was the discontinuation temporary? ○ Yes ○ No

Date of resuming study medication [ ] dd.mm.yyyy

Discontinuation or Modification of Study Medication
