# Supplementary material for: Clinical Surveillance vs. Anticoagulation For low-risk patiEnts with isolated SubSegmental Pulmonary Embolism: protocol for a multicentre randomised placebo-controlled non-inferiority trial (SAFE-SSPE)
Source: BMJ Open. 2020 Nov 19;10(11):e040151. doi: 10.1136/bmjopen-2020-040151 (PMC7678381; doi:10.1136/bmjopen-2020-040151)
Supplement: Author's manuscript [file bmjopen-2020-040151.draft_revisions.pdf]

BMJ Open

BMJ Open is committed to open peer review. As part of this commitment we make the peer review history of every article we publish publicly available.

When an article is published we post the peer reviewers' comments and the authors' responses online. We also post the versions of the paper that were used during peer review. These are the versions that the peer review comments apply to.

The versions of the paper that follow are the versions that were submitted during the peer review process. They are not the versions of record or the final published versions. They should not be cited or distributed as the published version of this manuscript.

BMJ Open is an open access journal and the full, final, typeset and author-corrected version of record of the manuscript is available on our site with no access controls, subscription charges or pay-per-view fees (http://bmjopen.bmj.com).

If you have any questions on BMJ Open's open peer review process please email info.bmjopen@bmj.com

Rationale and Design of Clinical Surveillance vs. Anticoagulation For low-risk patiEnts with isolated SubSegmental Pulmonary Embolism: a multicenter randomized placebo-controlled non-inferiority trial (SAFE-SSPE)

Journal:	*BMJ Open*
Manuscript ID	bmjopen-2020-040151
Article Type:	Protocol
Date Submitted by the Author:	06-May-2020

Complete List of Authors:	Baumgartner, Christine; Department of General Internal Medicine, Inselspital, Bern University Hospital, University of Bern, , General Internal Medicine<br>Klok, Frederikus; Leiden University Medical Centre, Department of Thrombosis and Hemostasis<br>Carrier, Marc; Ottawa Hospital Research Institute<br>Limacher, Andreas; Universitat Bern Institut fur Sozial- und Praventivmedizin<br>Moor, Jeanne; Inselspital University Hospital Bern, Department of General Internal Medicine<br>Righini, Marc; Geneva University Hospitals, Division of Angiology and Haemostasis<br>Beer, Jürg-Hans; Kantonsspital Baden AG, Department of Internal Medicine<br>Peluso, Martina; Inselspital University Hospital Bern, Department of General Internal Medicine<br>Rakovic, Damiana; Inselspital University Hospital Bern, Department of General Internal Medicine<br>Huisman, Menno; Leiden University Medical Centre, Department of Thrombosis and Hemostasis<br>AUJESKY , Drahomir; Bern University Hospital, Switzerland, Department of General Internal Medicine

Keywords:	Anticoagulation < HAEMATOLOGY, Thromboembolism < CARDIOLOGY, Clinical trials < THERAPEUTICS

SCHOLARONE™
Manuscripts

I, the Submitting Author has the right to grant and does grant on behalf of all authors of the Work (as defined in the below author licence), an exclusive licence and/or a non-exclusive licence for contributions from authors who are: i) UK Crown employees; ii) where BMJ has agreed a CC-BY licence shall apply, and/or iii) in accordance with the terms applicable for US Federal Government officers or employees acting as part of their official duties; on a worldwide, perpetual, irrevocable, royalty-free basis to BMJ Publishing Group Ltd ("BMJ") its licensees and where the relevant Journal is co-owned by BMJ to the co-owners of the Journal, to publish the Work in this journal and any other BMJ products and to exploit all rights, as set out in our licence.

The Submitting Author accepts and understands that any supply made under these terms is made by BMJ to the Submitting Author unless you are acting as an employee on behalf of your employer or a postgraduate student of an affiliated institution which is paying any applicable article publishing charge ("APC") for Open Access articles. Where the Submitting Author wishes to make the Work available on an Open Access basis (and intends to pay the relevant APC), the terms of reuse of such Open Access shall be governed by a Creative Commons licence – details of these licences and which Creative Commons licence will apply to this Work are set out in our licence referred to above.

Other than as permitted in any relevant BMJ Author's Self Archiving Policies, I confirm this Work has not been accepted for publication elsewhere, is not being considered for publication elsewhere and does not duplicate material already published. I confirm all authors consent to publication of this Work and authorise the granting of this licence.

For peer review only

Rationale and Design of Clinical Surveillance vs. Anticoagulation For low-risk patiEnts with isolated SubSegmental Pulmonary Embolism: a multicenter randomized placebo-controlled non-inferiority trial (SAFE-SSPE)

Christine Baumgartner, MD, MAS [1]; Frederikus A. Klok, MD, PhD, FESC [2]; Marc Carrier, MD, MSc [3]; Andreas Limacher, PhD [4]; Jeanne Moor, MD [1]; Marc Righini, MD [5]; Jürg-Hans Beer, MD [6]; Martina Peluso [1]; Damiana Rakovic [1]; Menno V. Huisman, MD, PhD [2]; Drahomir Aujesky, MD, MSc [1]

Author Affiliations:

[1] Department of General Internal Medicine, Inselspital, Bern University Hospital, University of Bern, Switzerland

[2] Department of Thrombosis and Hemostasis, Leiden University Medical Center, Leiden, the Netherlands

[3] Department of Medicine, The Ottawa Hospital Research Institute at the University of Ottawa, Ottawa, Canada

[4] CTU Bern, University of Bern, Bern, Switzerland

[5] Division of Angiology and Haemostasis, Geneva University Hospital, University of Geneva, Switzerland

[6] Department of Internal Medicine, Cantonal Hospital of Baden, Baden, Switzerland

Corresponding Author:

Christine Baumgartner, MD, MAS, Department of General Internal Medicine, Inselspital, Bern University Hospital, University of Bern, Freiburgstrasse, 3010 Bern, Switzerland. Phone: +41 (0)31 632 57 69; e-mail: Christine.Baumgartner@insel.ch

For peer review only

For peer review only - http://bmjopen.bmj.com/site/about/guidelines.xhtml

Abstract word count: 281, **Text word count:** 3979**, Tables:** 3**, Figures:** 1**, Number of References:** 66

Keywords: subsegmental pulmonary embolism, anticoagulation, randomized clinical trial

For peer review only

Abstract

Introduction: The clinical significance of subsegmental pulmonary embolism (SSPE) is currently unclear. Although growing evidence from observational studies suggests that withholding anticoagulant treatment may be a safe option in selected patients with isolated SSPE, most patients with this condition receive anticoagulant treatment, which is associated with a 90-day risk of recurrent VTE of 0.8% and major bleeding of up to 5%. Given the ongoing controversy concerning the risk-benefit ratio of anticoagulation for isolated SSPE and the lack of evidence from randomized-controlled studies, the aim of this clinical trial is to evaluate the efficacy and safety of clinical surveillance without anticoagulation in low-risk patients with isolated SSPE.

Methods and Analysis: SAFE-SSPE (Surveillance vs. Anticoagulation For low-risk patiEnts with isolated SubSegmental Pulmonary Embolism: a multicenter randomized placebo-controlled non-inferiority trial) is an international, multicenter, placebo-controlled, double-blind, parallel-group non-inferiority trial conducted in Switzerland, the Netherlands, and Canada. Low-risk patients with isolated SSPE are randomized to receive clinical surveillance with either placebo (no anticoagulation) or anticoagulant treatment with rivaroxaban. All patients undergo bilateral whole-leg compression ultrasonography to exclude concomitant deep vein thrombosis before enrolment. Patients are followed for 90 days. The primary outcome is symptomatic recurrent VTE (efficacy). The secondary outcomes include clinically significant bleeding and all-cause mortality (safety). The ancillary outcomes are health-related quality of life, functional status, and medical resource utilization.

Ethics and Dissemination: The local Ethics Committees in Switzerland have approved this protocol. Submission to the Ethical Committees in the Netherlands and Canada is underway. The results of this trial will be published in a peer-reviewed journal.

Main Funding: Investigator Initiated Clinical Trials funding scheme of the Swiss National Science Foundation (SNSF). The study medication is provided free of charge by Bayer AG.

For peer review only - http://bmjopen.bmj.com/site/about/guidelines.xhtml

Trial Registration Numbers:

ClinicalTrials.gov: NCT04263038

Protocol version 2.0, 23.01.2020

For peer review only

For peer review only - http://bmjopen.bmj.com/site/about/guidelines.xhtml

Article Summary: Strengths and Limitations of this Study

- This is the first randomized trial comparing the safety and efficacy of a management strategy without anticoagulation and anticoagulant treatment in low-risk patients with isolated subsegmental pulmonary embolism (SSPE).
- We chose patient-centered outcomes and economically relevant efficiency measures that are relevant to both patient and health care professionals.
- If we show non-inferiority, the results of this trial will provide a strong scientific basis for withholding anticoagulation in low-risk patients with SSPE and has the potential to reduce bleeding complications and patient harm.
- As patients with isolated SSPE and a high risk of adverse events (e.g., patients with cancer) are excluded from this trial due to safety reasons, generalizability of the results will be limited to low-risk patients with isolated SSPE.

For peer review only

For peer review only - http://bmjopen.bmj.com/site/about/guidelines.xhtml

Introduction

Depending on thromboembolic burden and patient factors, the clinical spectrum of pulmonary embolism (PE) ranges from asymptomatic cases to massive PE with hemodynamic collapse.[1,2] Anticoagulant treatment for at least 3 months effectively reduces the risk of recurrent venous thromboembolism (VTE).[3 4] The benefit of anticoagulation, however, comes at the cost of potentially disabling and life-threatening bleeding events, with a 90-day risk of major bleeding of up to 5%.[5-8]

The widespread introduction and technological advances of multi-detector computed tomography pulmonary angiography (CTPA) have led to an 80% increase in the diagnosis of acute PE between 1998 and 2006.[9] This increase is in part due to an increase in the detection of small, peripheral PE limited to the subsegmental pulmonary arteries, i.e. subsegmental PE (SSPE),[10,11] which currently comprise 10-15% of cases with PE.[12-15] However, the clinical significance of isolated SSPE is questionable. Epidemiologic evidence suggests overdiagnosis: despite the increase in PE diagnoses in recent years, PE-related mortality has remained stable or has even decraesed.[9,16] The positive predictive value of CTPA to diagnose SSPE is as low as 25% (compared to a composite reference standard with ventilation-perfusion lung scanning with or without lower limb venous ultrasonography, or pulmonary digital-subtraction angiography),[17] and inter-observer agreement between radiologists for SSPE diagnosis is only fair,[18] indicating that differentiation between true emboli and artifacts in the subsegmental pulmonary arteries is difficult. Small PE may even occur in healthy individuals without clinical consequences,[19,2021] suggesting that SSPE may be the result from of the physiological filter function of the lung to protect the systemic circulation.

Whether patients with isolated SSPE benefit from anticoagulant treatment is currently uncertain.[4] There is growing evidence from observational studies that withholding anticoagulation may be safe in low-risk patients with isolated SSPE,[22-27] but most of such patients currently receive anticoagulant treatment,[28-31] potentially exposing them to an unnecessary risk of bleeding. Given the ongoing

For peer review only - http://bmjopen.bmj.com/site/about/guidelines.xhtml

controversy and clinical equipoise about the risk-benefit ratio of anticoagulation for isolated SSPE, we aim to evaluate the efficacy and safety of clinical surveillance without anticoagulation compared to standard anticoagulation treatment in low-risk patients with SSPE in a randomized clinical trial.

For peer review only

For peer review only - http://bmjopen.bmj.com/site/about/guidelines.xhtml

Methods and Analysis

This study protocol has been developed according to the Standard Protocol Items: Recommendation for Interventional Trials (SPIRIT) guidelines.[32]

Objectives and Hypotheses

The primary objective of this randomized trial is to compare the frequency of symptomatic, recurrent VTE in low-risk patients with isolated SSPE randomized to receive clinical surveillance plus placebo or clinical surveillance plus anticoagulant treatment with rivaroxaban. As a secondary objective, the frequency of clinically significant bleeding and all-cause mortality is compared in the two groups. Ancillary endpoints include health-related quality of life, functional status, and medical resource utilization. We hypothesize that clinical surveillance without anticoagulant treatment is non-inferior to anticoagulation in terms of recurrent VTE, while resulting in fewer clinically significant bleeding events and similar all-cause mortality. We also hypothesize that clinical surveillance, compared to anticoagulation, improves health-related quality of life and functional status and reduces medical resource utilization.

Study Design and Setting

SAFE-SSPE (clinical Surveillance vs. Anticoagulation For low-risk patiEnts with isolated SubSegmental Pulmonary Embolism) is an investigator-initiated, multicenter, randomized, placebo-controlled, double-blind, parallel-group non-inferiority trial. Low-risk patients with isolated SSPE without concomitant deep vein thrombosis (DVT) are randomly assigned in a 1:1 ratio to receive clinical surveillance plus placebo or clinical surveillance plus anticoagulation with rivaroxaban (**Figure**). Randomization is blocked and stratified by study site. To ensure concealment of allocation, the allocation sequence is generated by a data manager not involved in the study, using a computer-generated randomization schedule. All participants, care providers, investigators, study personnel,

For peer review only - http://bmjopen.bmj.com/site/about/guidelines.xhtml

members of the outcomes adjudication committee, data management personnel, and analysts are blinded to group assignment. Eligible patients are recruited in at least 27 university and medium- to high-volume non-university teaching hospitals in Switzerland, the Netherlands, and Canada.

Selection of Patients

Consecutive patients aged ≥18 years with objectively diagnosed symptomatic or asymptomatic isolated SSPE on CTPA based on the assessment of the local radiologist at the time of patient presentation are eligible for study participation after provision of informed consent (**Table 1**). Isolated SSPE is defined as multi-detector CTPA demonstrating an intraluminal filling defect in ≥1 subsegmental pulmonary artery (4th order or higher) without filling defects visualized at more proximal pulmonary artery levels.[24,33,34] Isolated subsegmental defects are classified as either single (1 subsegmental vessel involved) or multiple (≥2 subsegmental vessels involved).

The exclusion criteria were selected based on a high early risk of VTE recurrence (≥8%), the presence of cardiopulmonary compromise, contraindications to anticoagulant treatment with rivaroxaban, and a high risk of confounding (**Table 2**). Because PE usually originates from a thrombus in the deep leg veins,[35] potentially eligible patients systematically receive a single bilateral whole-leg compression ultrasonography (CUS) examination to exclude concomitant DVT, which is a well-known prognostic factor for mortality in patients with PE and represents an absolute indication for therapeutic anticoagulation.[36] If a proximal or distal DVT (with an incompressible distal vein diameter of ≥5mm)[37] is detected, patients are excluded from study participation and treated at the discretion of their managing physician. All exclusion criteria are listed in **Table 2**.

Intervention

Patients in the intervention group receive clinical surveillance and a matching rivaroxaban placebo using the same dosing schedule as for rivaroxaban (see below). Clinical surveillance is done

For peer review only - http://bmjopen.bmj.com/site/about/guidelines.xhtml

during the follow-up interviews by phone or in-person, depending on local practice. At each contact, trained study personnel complete an assessment of symptoms and review for suspected recurrent VTE and bleeding. If patients report symptoms or signs suggestive of recurrent VTE or significant bleeding, they are asked to present immediately to an ED for evaluation.

Control

Patients who are assigned to the anticoagulation group receive rivaroxaban 15 mg twice daily for the first 21 days, followed by 20 mg once daily for the remaining 90-day study period. After completion of the treatment period, the study drug is discontinued. The same surveillance schedule as in the intervention group is used.

Study Procedures

Patients are screened for eligibility if they receive a CTPA in the emergency department or within 72 hours of hospitalization to rule-in or rule-out PE, or if they present to an outpatient service within 72 hours of SSPE diagnosis. Eligibility criteria are assessed for all consecutive patients with suspected SSPE, and potentially eligible patients are asked to provide informed consent by investigators or their delegates. For those who consent to participate, a bilateral whole-leg CUS is performed by qualified examiners. All eligible and consenting patients without DVT are randomized to receive clinical surveillance or anticoagulation with rivaroxaban, and trained study personnel collect baseline data by reviewing medical records, medication lists, laboratory results that have been obtained as part of routine care, radiology reports, and by patient interview (**Table 3**). After provision of the assigned study medication, the participants are asked to immediately start with the treatment. In addition, all participants receive a patient diary consisting of two parts: the first contains important information

For peer review only - http://bmjopen.bmj.com/site/about/guidelines.xhtml

about the study, and the second part consists of a diary to record dates and type of outcomes in order to minimize recall bias (**Table 3**).

Participants are followed for 90 days using phone or in-person interviews at 10, 30, and 90 days after randomization. Trained study personnel contacts patients, family members, and/or primary care physicians and review medical charts to obtain information about outcome events. At the end of the treatment period, patients are instructed to return the medication bottles and the patient diary. Drug-adherence is assessed by counting the pills in the returned medication bottles (**Table 3**).

Data Collection and Quality

Data are collected using an electronic database (secuTrial®). The following measures are implemented to ensure optimal data quality and completeness: 1) training of study personnel in the methods of data abstraction, patient inquiry, and data recording, 2) recording of study data on standardized electronic case report forms (eCRFs), 3) operations manual providing information on definitions and acceptable data sources for all variables, 4) central data monitoring with generation of statistical reports and individual data checks, and 5) risk based on-site monitoring.

Criteria for Discontinuation of the Study Medication and Unblinding

In participants requiring prophylactic or therapeutic anticoagulation during the study period for reasons other than the index SSPE or if treatment with another prohibited agent is necessary (i.e. strong CYP3A4 inhibitors or inducers, dual antiplatelet therapy, GP IIb/IIIa inhibitors), the study drug should be temporarily interrupted and restarted as soon as possible following discontinuation of the prohibited medication. Patients who develop any condition requiring permanent therapeutic anticoagulation (e.g., atrial fibrillation) should permanently discontinue the study drug. If an invasive procedure or surgical intervention is required, the study medication should be stopped at least 24 hours prior to an elective

For peer review only - http://bmjopen.bmj.com/site/about/guidelines.xhtml

intervention, or immediately for emergency procedures. If objectively confirmed recurrent VTE or pregnancy is diagnosed during the treatment period, the study drug should be discontinued and treatment allocation unblinded. Similarly, in case of major bleeding, the study drug should be stopped, and unblinding may be necessary if emergency anticoagulation reversal is indicated.

Primary and Secondary Outcomes

The primary (efficacy) outcome is the proportion of recurrent, clinically symptomatic, objectively confirmed VTE within 90 days of randomization, defined as recurrent PE or lower limb DVT.[38,39] The objective diagnostic criterion for PE, based on available radiographic reports, is a new intraluminal filling defect on CTPA or pulmonary angiography; a perfusion defect involving at least 75% of a segment, with corresponding normal ventilation (i.e., high probability lung scan); the confirmation of new PE at autopsy; or objectively confirmed proximal DVT of the lower extremity in patients with symptoms of PE. The objective diagnosis of DVT is the non-compressibility of a venous segment on CUS or an intraluminal filling defect on contrast venography. Because compression of iliac veins and the inferior vena cava may be technically difficult, additional diagnostic criteria for iliac and caval DVT also include abnormal duplex flow patterns compatible with thrombosis or an intraluminal filling defect on computed tomography or magnetic resonance imaging venography.[40] Both proximal and distal DVTs are considered.

Secondary (safety) outcomes include the proportion of clinically significant bleeding and all-cause mortality 90 days following randomization. Clinically significant bleeding is a composite endpoint of major and clinically relevant non-major bleeding. Major bleeding is defined as fatal bleeding, symptomatic bleeding at critical sites (intracranial, intraspinal, intraocular, retroperitoneal, intraarticular, pericardial, or intramuscular with compartment syndrome), or bleeding with a reduction of hemoglobin ≥20 g/L, or bleeding leading to transfusion of ≥2 units of packed red blood cells according to the definition of the International Society on Thrombosis and Haemostasis.[41] Clinically relevant non-

For peer review only - http://bmjopen.bmj.com/site/about/guidelines.xhtml

For peer review only

major bleeding is defined as overt bleeding that does not meet criteria for major bleeding but is associated with a medical intervention, unscheduled physician contact (visit or telephone call), temporary cessation of the study drug, pain, or impairment of activities of daily life.[42]

Information about the date, type, and circumstances of outcome events is obtained from patients, family members, and/or health care providers during the follow-up interviews or by reviewing medical charts. In patients who experience recurrent VTE, the radiographic report and images confirming VTE recurrence are obtained. For patients who died during follow-up, the cause of death based on medical reports, death certificates, and autopsy reports (if available) is recorded. All medical outcome events are reviewed and adjudicated by a committee of 3 independent clinical experts unaware of treatment assignment. Based on available information, death is adjudicated as PE-related, due to major bleeding (any death following an intracranial hemorrhage or a bleeding episode leading to hemodynamic deterioration), due to another cause, or due to an undetermined cause. Death is considered PE-related in the following situations: a) autopsy-confirmed PE in the absence of another more likely cause of death, b) objectively confirmed PE within the last 48 hours before death in the absence of another more likely cause of death, or c) PE is not objectively confirmed, but is most likely the main cause of death.[43] Final classification of all medical outcomes is based on the full consensus of the committee.

Ancillary Outcomes

Ancillary outcomes include health-related quality of life and functional status, both important patient-centered outcomes, and medical resource utilization, an economically relevant efficiency measure. Disease-specific, health-related quality of life at baseline, 30 and 90 days after the index PE is assessed using the Pulmonary Embolism Quality of Life (PEmb-QoL) questionnaire.[44,45] The PEmb-QoL is a validated, self-administered 40-item questionnaire to quantify quality of life in patients having

For peer review only - http://bmjopen.bmj.com/site/about/guidelines.xhtml

experienced PE. Functional status is measured at 90 days after randomization using the post-VTE functional status scale.[46] This scale has been proposed to assess functional limitations after VTE, covering aspects of daily life (including limitations in usual activity and changes in lifestyle) that are affected by the consequences of VTE and its complications. The assessment is done using a short, structured interview 90 days after randomization.

The following measures of medical resource utilization and productivity are assessed:[47,48] initial length of stay (LOS), subsequent overall hospitalizations as well as overall ED and physician outpatient visits within 90 days of randomization, and time to return to work in workers or usual activities (e.g., household) in non-workers. Information on these outcomes are obtained from the participant during the follow-up interviews, by interview of the patient's primary care physician, and by hospital chart review. Subsequent health care contacts are classified as potentially related to VTE if a patient had chest or leg symptoms or signs (dyspnea, chest pain, pleural effusion, or leg pain or swelling), or bleeding complications.

Withdrawals and Loss to Follow-Up

Given the low treatment and follow-up burden and the short follow-up period of 90 days, we expect that completeness of follow-up data collection will be close to 100% based on prior experience.[47] A patient who withdraws consent for follow-up at 10 or 30 days may still agree with passive follow-up (i.e. that study personnel may collect follow-up information from medical records of the participant's primary care physician) or to continue with the assessment at 90 days, if given the option. If study withdrawal occurs, data collected up to the time of withdrawal is used in a coded manner. If a participant is lost to follow-up, primary care physicians and surrogates are contacted, and the hospital records are consulted to obtain information about primary and secondary outcomes and survival status.

For peer review only - http://bmjopen.bmj.com/site/about/guidelines.xhtml

Post-hoc Evaluation of Radiologic Inter-Observer Agreement

CTPA images undergo central review at the Bern University Hospital by a panel of two experienced thoracic radiologists blinded to the interpretation of the radiologist at the enrolling site. Based on the consensus of this panel, the initial CTPA readings are classified into 3 categories: 1) presence of isolated SSPE (true-positive isolated SSPE), 2) absence of any PE (false-positive isolated SSPE), and 3) presence of segmental, lobar, or central PE (false-negative isolated SSPE). The panel also evaluates the technical quality of the CTPA examination (adequacy of opacification, breathing artifacts) and the number of filling defects (i.e., single vs. multiple isolated SSPE). The central review of CTPA images is done post-hoc in 6-month batches.

Sample Size Calculation

Assumptions on VTE recurrence risk are based on data from 127 low-risk patients with isolated SSPE who received anticoagulants (warfarin or low-molecular-weight heparin), showing a VTE recurrence risk of 0.8% at 90 days after diagnosis.[23] We chose an absolute non-inferiority margin of 3.5% on the basis of recruitment feasibility, clinical acceptability, and previous studies. This corresponds to a difference which is considered acceptable by most physicians and patients for the following reasons. First, our margin is within the range of the 3-month VTE recurrence proportion (0.5-5%) below which thrombosis specialists would not initiate anticoagulation for PE.[49] Second, the definition of a clinically acceptable non-inferiority margin for recurrent VTE must also take into account the potential benefits of withholding anticoagulation, i.e. the substantially lower risk of clinically significant bleeding (<1% versus 7% within 3 months for patients receiving anticoagulants).[5,23,25,50] Indeed, a patient group with PE who was involved in the trial planning process indicated that given the bleeding risk associated with anticoagulants, a VTE recurrence proportion of <5% seemed acceptable. Finally, similar non-inferiority

For peer review only - http://bmjopen.bmj.com/site/about/guidelines.xhtml

margins (3-5%) have been used in key studies comparing different drug treatment regimens and inpatient vs. outpatient management for acute VTE.[47,51-56]

To determine the sample size, we used a Monte-Carlo simulation approach based on an Agresti-Caffo confidence interval (CI) for risk difference.[57] Assuming a baseline VTE recurrence proportion of 1.0% at 90 days in both treatment groups, an absolute margin of 3.5% defining non-inferiority for clinical surveillance, and a sampling ratio of 1:1 allowing 5% attrition (dropouts, including patients who died from non-VTE-related causes) in each group during 90 days, we estimated that 276 patients (138 per group) would result in at least 80% power to establish non-inferiority at an one-sided type I error of 5%.

Planned Statistical Analyses

As recommended by the CONSORT statement for non-inferiority trials,[58] we will perform both intention-to-treat (ITT) and per-protocol (PP) analyses. For the primary outcome, ITT and PP analyses should reach the same conclusion to consider results to be robust. For the remaining outcomes, the ITT analysis will be the primary analysis, the PP analysis a secondary analysis.

In the ITT analysis, all randomized patients will be analyzed within the treatment group to which they were randomized. In the PP analysis, patients with protocol violations will be excluded (crossover to study treatment from other group; patients receiving a different type or dose of anticoagulation than requested by the study protocol; patients with missing primary outcome data; patients violating relevant eligibility criteria; patients stopping study treatment within 1 month after randomization, or patients in whom the diagnosis of isolated SSPE was refuted by the central CTPA review panel).

We will describe the prevalence of recurrent VTE, clinically significant bleeding (including its individual components, major and clinically relevant non-major bleeding), and all-cause mortality at 90 days after randomization with 95% Wilson CIs by treatment group. Non-inferiority of the primary outcome (VTE recurrence) among patients in the clinical surveillance vs. the anticoagulation group will

For peer review only - http://bmjopen.bmj.com/site/about/guidelines.xhtml

be assessed based on the Agresti-Caffo CI for risk difference.[57] If the upper limit of the one-sided 95% Agresti-Caffo CI will be lower than the pre-specified non-inferiority margin, clinical surveillance will be considered non-inferior to anticoagulation. For secondary outcomes, we will calculate the risk difference with a two-sided 95% Agresti-Caffo CI and compare groups using an exact binomial test.[57]

PEmb-QoL dimension scores at 30 and 90 days as well as the change in scores from baseline will be presented by treatment group as means with 95% CIs. The change in dimension scores and the differences in the change between groups will be analyzed using a repeated-measures, linear mixed-effects model adjusted for the respective baseline value. Functional status, which is measured on a scale from 0 to 5 at 90 days, will be presented as median and interquartile range and compared between groups using the non-parametric Wilcoxon rank-sum test. Count data (subsequent hospitalizations, outpatient visits) will be presented by treatment group as rate with an exact 95% Poisson CI and compared using a rate ratio and exact p-value. Time-to-event outcomes (initial LOS, time to return to work/usual activities) will be presented as medians and interquartile ranges. For LOS, we will compare groups using the Wilcoxon rank-sum test. For return to work, we will display Kaplan-Meier curves and compare groups by the log-rank test. An alpha level of <0.05 will define statistical significance.

In secondary analyses, we will calculate the cumulative incidence and the difference in cumulative incidence of VTE recurrence and clinically significant bleeding, correcting for withdrawals and losses to follow-up by censoring and for death unrelated to VTE recurrence or bleeding as a competing event. All-cause mortality will be assessed likewise, however a competing event does not apply. In a further secondary analysis, we will use model-based approaches for all outcomes. For time-to-event outcomes (VTE recurrence, clinically significant bleeding, LOS, return to work/usual activity), we will use competing risk regression according to Fine and Gray,[59] accounting for non-VTE/non-bleeding-related death or all-cause death as a competing event. For mortality, we will use Cox

For peer review only - http://bmjopen.bmj.com/site/about/guidelines.xhtml

regression. For count data, we will use a negative binomial model, and also consider zero-inflation. In case of heterogeneity across sites, we will adjust models for site using random-effects models.

Data Monitoring and Interim Safety Analysis

An independent Data and Safety Monitoring Board (DSMB) consisting of 3 members unaffiliated with any of the participating institutions will evaluate unblinded interim safety results after 100 and 190 randomized patients have completed the 90-day follow-up. To monitor recurrent VTE in the intervention group, formal interim analyses will be performed using a Bayesian approach. Based on literature,[23] we expect a frequency of VTE recurrence of 1.0% (0-4.5%). We regard a frequency of >5% in the surveillance group as clinically unacceptable. We will calculate the prior probability distribution of events based on the expected frequency as well as the posterior probability distribution using actually observed data at each interim analysis. Based on the posterior distribution, we will calculate the probability that the true proportion of VTE recurrence in the surveillance group exceeds the threshold of unacceptable frequency. If the probability of exceedance is >70%, the surveillance arm will be considered inferior and the DSMB will recommend to stop the trial. The final decision will lie with the Steering Committee. This procedure is conservative regarding the type-I error rate; therefore, we will not adjust the significance level in the final analysis.

Patient and Public Involvement

We have partnered with a group of patients who had recently experienced PE in the planning process of this trial, including the selection of patient-centered outcomes, the establishment of a safe surveillance schedule, and the determination of a clinically acceptable non-inferiority margin. Patients are not involved in the recruitment and conduct of the study or the dissemination of study results.

For peer review only - http://bmjopen.bmj.com/site/about/guidelines.xhtml

Data Sharing

After publication of the study results, a de-identified patient-level data set relating to the primary publication along with the latest version of the study protocol, the informed consent form, the statistical analysis plan, the analysis code, and the data management plan of the study will be made publicly available in the *Bern Open Repository and Information System* (*BORIS) Research Data*.

Ethics and Dissemination

This trial is conducted in accordance with the Declaration of Helsinki, the International Council on Harmonization Good Clinical Practice (ICH-GCP) guidelines, and all applicable national legal and regulatory requirements. Authorization by the local Ethics Committees and Swissmedic has been obtained in Switzerland, and the submission process to the relevant Dutch and Canadian Ethics Committees and regulatory authorities is ongoing. All changes in research activity or unanticipated problems involving risks to human subjects will be reported promptly to the Ethics Committees. All participating investigators/institutions will permit study-related monitoring, audits, Ethics Committee reviews, and regulatory inspections, and will provide direct access to source documents and data. Protection of confidentiality is ensured according to regulatory requirements.

The primary study results will be presented at scientific conferences and published in a peer-reviewed medical journal. We also plan local presentations for physicians at the participating sites. Participants will receive a letter with the study results explained in lay language. Further public dissemination of the results is planned through publications in the lay press and via social media.

Conclusion

The SAFE-SSPE trial addresses an important gap of knowledge, the optimal management of SSPE, and represents the first randomized, direct comparison of clinical surveillance alone versus

For peer review only - http://bmjopen.bmj.com/site/about/guidelines.xhtml

anticoagulant treatment for low-risk patients with isolated SSPE. As the number of SSPE patients who may eventually receive anticoagulant treatment is likely to rise in the future with the further dissemination and advancement of CTPA technology, a strong scientific basis for withholding anticoagulation in low risk patients with SSPE is urgently needed. The results of this trial have the potential to improve quality and efficiency of care by reducing bleeding episodes and resource utilization and increasing health-related quality of life.

Current Status of the SAFE-SSPE trial

It is planned to start patient recruitment in Mai 2020. Follow-up of the last participant is expected to be completed in February 2024.

For peer review only

For peer review only - http://bmjopen.bmj.com/site/about/guidelines.xhtml

Authors' Contributions

Study concept and design: DA, MH, ST, MC, FAK, JHB, MR, AL, CB

Drafting of the manuscript: CB, DA

Critical revision of the manuscript for important intellectual content: MH, ST, MC, FAK, JHB, MR, AL, JM, MP, DR

Statistical analysis plan: AL, ST

Obtained funding: DA, MR, JHB

Administrative, technical, or material support: MP, JM, DR, CB

Supervision: DA

Acknowledgements

We would like to thank Sven Trelle, Director of the CTU Bern (Clinical Trials Unit) for his methodological inputs and organizational work concerning this study.

Funding Statement

This study is supported by an Investigator Initiated Clinical Trials (IICT) grant of the Swiss National Science Foundation (SNSF 33IC30_185616). The study medication was provided free of charge by Bayer AG.

Competing Interests Statement

The authors declare that there is no competing interest with regard to the intellectual concern and proprietary of affairs. The funding source had no role in the design of this study and does not have any role in the conduct, the collection, management, analysis, and interpretation of the data, the writing of the manuscript, or the decision to submit the results for publication.

For peer review only - http://bmjopen.bmj.com/site/about/guidelines.xhtml

Conveyance and Peer Review

This trial protocol has been peer-reviewed for approval of funding and ethical approval prior to submission.

Roles and Responsibilities

Sponsor-Investigator:

Prof. Drahomir Aujesky, MD, MSc, Department of General Internal Medicine, Inselspital, Bern University Hospital, University of Bern, Freiburgstrasse, CH-3010 Bern, Switzerland. Phone: +41 (0)31 632 88 84; e-mail: drahomir.aujesky@insel.ch

Role: sponsor-investigator, coordinating principal investigator, chair of the Steering Committee; responsible for the study design, overseeing the management of the study, interpretation of the data, and writing of the manuscript.

Legal Representative of the Sponsor in the Netherlands:

Frederikus A. Klok, MD, PhD, FESC, Leiden University Medical Center, Albinusdreef 2, NL-2333 ZA Leiden, The Netherlands. Phone: +31 71 529 8127; e-mail: F.A.Klok@lumc.nl

role: Steering Committee member, venous thromboembolism expert, site investigator, recruitment, legal representative of the sponsor in the Netherlands

Legal representative of the Sponsor in Canada:

Prof. Marc Carrier, MD, MSc, Department of Medicine, The Ottawa Hospital Research Institute at the University of Ottawa, CA-Ottawa, K1H8L6, Canada. Phone: +1 613 737 8899, e-mail: mcarrier@toh.ca

role: Steering Committee member, venous thromboembolism expert, site investigator, recruitment, legal representative of the sponsor in Canada

For peer review only

Steering Committee:

Chair: Prof. Drahomir Aujesky, MD, MSc

Steering committee members: Prof. Jürg-Hans Beer, MD; Prof. Marc Carrier, MD, MSc; Dr. Frederikus A. Klok, MD, PhD, FESC; Prof. Marc Righini, MD

The study steering committee is responsible for the overall supervision of the trial and regularly meets to discuss scientific and logistic aspects of the trial.

Monitoring Institution and Data Management:

CTU Bern (Clinical Trials Unit), University of Bern, Mittelstrasse 43, CH-3012 Bern, Switzerland. Phone: +41 (0)31 631 35 04; e-mail: sven.trelle@ctu.unibe.ch

Date Safety Monitoring Board:

An independent Data Safety Monitoring Board (DSMB) evaluates unblinded VTE outcomes on a regular basis and make recommendations to the Steering Committee to amend or terminate the trial should any safety concerns arise. The DSMB includes 3 members unaffiliated with Bern University Hospital or any research partners: 2 experts in the area of VTE, and 1 expert in statistics and methodology of randomized trials.

Other Relevant Committees and Institutions:

The study is managed and coordinated at the Inselspital, Bern University Hospital.

An independent outcome adjudication committee consisting of 3 clinical experts at the Inselspital, Bern University Hospital, is responsible for adjudication of the primary and secondary outcomes events in a blinded fashion.

For peer review only - http://bmjopen.bmj.com/site/about/guidelines.xhtml

For peer review only

A central CT review panel, consisting of two experienced thoracic radiologists at the Inselspital, Bern, University Hospital, centrally reviews all CTPA images.

The Hospital Pharmacy of the Inselspital, Bern University Hospital, is in charge of the pharmaceutical tasks.

For peer review only

For peer review only - http://bmjopen.bmj.com/site/about/guidelines.xhtml

References

1. Di Nisio M, van Es N, Buller HR. Deep vein thrombosis and pulmonary embolism. *Lancet.* 2016;388(10063):3060-3073.

2. Huisman MV, Barco S, Cannegieter SC, et al. Pulmonary embolism. *Nat Rev Dis Primers.* 2018;4:18028.

3. Castellucci LA, Cameron C, Le Gal G, et al. Clinical and safety outcomes associated with treatment of acute venous thromboembolism: a systematic review and meta-analysis. *JAMA.* 2014;312(11):1122-1135.

4. Kearon C, Akl EA, Ornelas J, et al. Antithrombotic Therapy for VTE Disease: CHEST Guideline and Expert Panel Report. *Chest.* 2016;149(2):315-352.

5. Buller HR, Prins MH, Lensin AW, et al. Oral rivaroxaban for the treatment of symptomatic pulmonary embolism. *N Engl J Med.* 2012;366(14):1287-1297.

6. Jun M, Lix LM, Durand M, et al. Comparative safety of direct oral anticoagulants and warfarin in venous thromboembolism: multicentre, population based, observational study. *Bmj.* 2017;359:j4323.

7. Linkins LA, Choi PT, Douketis JD. Clinical impact of bleeding in patients taking oral anticoagulant therapy for venous thromboembolism: a meta-analysis. *Ann Intern Med.* 2003;139(11):893-900.

8. Scherz N, Mean M, Limacher A, et al. Prospective, multicenter validation of prediction scores for major bleeding in elderly patients with venous thromboembolism. *J Thromb Haemost.* 2013;11(3):435-443.

9. Wiener RS, Schwartz LM, Woloshin S. Time trends in pulmonary embolism in the United States: evidence of overdiagnosis. *Arch Intern Med.* 2011;171(9):831-837.

For peer review only - http://bmjopen.bmj.com/site/about/guidelines.xhtml

10. Auer RC, Schulman AR, Tuorto S, et al. Use of helical CT is associated with an increased incidence of postoperative pulmonary emboli in cancer patients with no change in the number of fatal pulmonary emboli. *J Am Coll Surg.* 2009;208(5):871-878; discussion 878-880.

11. Ikesaka R, Carrier M. Clinical significance and management of subsegmental pulmonary embolism. *J Thromb Thrombolysis.* 2015;39(3):311-314.

12. Carrier M, Righini M, Wells PS, et al. Subsegmental pulmonary embolism diagnosed by computed tomography: incidence and clinical implications. A systematic review and meta-analysis of the management outcome studies. *J Thromb Haemost.* 2010;8(8):1716-1722.

13. Carrier M, Righini M, Le Gal G. Symptomatic subsegmental pulmonary embolism: what is the next step? *J Thromb Haemost.* 2012;10(8):1486-1490.

14. Stein PD, Goodman LR, Hull RD, Dalen JE, Matta F. Diagnosis and management of isolated subsegmental pulmonary embolism: review and assessment of the options. *Clin Appl Thromb Hemost.* 2012;18(1):20-26.

15. van der Pol LM, Bistervels IM, van Mens TE, et al. Lower prevalence of subsegmental pulmonary embolism after application of the YEARS diagnostic algorithm. *Br J Haematol.* 2018.

16. Barco S, Mahmoudpour SH, Valerio L, et al. Trends in mortality related to pulmonary embolism in the European Region, 2000-15: analysis of vital registration data from the WHO Mortality Database. *Lancet Respir Med.* 2020;8(3):277-287.

17. Stein PD, Fowler SE, Goodman LR, et al. Multidetector computed tomography for acute pulmonary embolism. *N Engl J Med.* 2006;354(22):2317-2327.

18. Ghanima W, Nielssen BE, Holmen LO, Witwit A, Al-Ashtari A, Sandset PM. Multidetector computed tomography (MDCT) in the diagnosis of pulmonary embolism: interobserver agreement among radiologists with varied levels of experience. *Acta Radiol.* 2007;48(2):165-170.

For peer review only

For peer review only - http://bmjopen.bmj.com/site/about/guidelines.xhtml

19. Gurney JW. No fooling around: direct visualization of pulmonary embolism. *Radiology.* 1993;188(3):618-619.

20. Schoepf UJ, Costello P. CT angiography for diagnosis of pulmonary embolism: state of the art. *Radiology.* 2004;230(2):329-337.

21. Carrier M, Klok FA. Symptomatic subsegmental pulmonary embolism: to treat or not to treat? *Hematology Am Soc Hematol Educ Program.* 2017;2017(1):237-241.

22. Cha SI, Shin KM, Lee JW, et al. Clinical characteristics of patients with peripheral pulmonary embolism. *Respiration.* 2010;80(6):500-508.

23. Donato AA, Khoche S, Santora J, Wagner B. Clinical outcomes in patients with isolated subsegmental pulmonary emboli diagnosed by multidetector CT pulmonary angiography. *Thromb Res.* 2010;126(4):e266-270.

24. Le Gal G, Righini M, Parent F, van Strijen M, Couturaud F. Diagnosis and management of subsegmental pulmonary embolism. *J Thromb Haemost.* 2006;4(4):724-731.

25. Mehta D, Barnett M, Zhou L, et al. Management and outcomes of single subsegmental pulmonary embolus: a retrospective audit at North Shore Hospital, New Zealand. *Intern Med J.* 2014;44(9):872-876.

26. Bariteau A, Stewart LK, Emmett TW, Kline JA. Systematic review and meta-analysis of outcomes of patients with subsegmental pulmonary embolism with and without anticoagulation treatment. *Acad Emerg Med.* 2018.

27. Eyer BA, Goodman LR, Washington L. Clinicians' response to radiologists' reports of isolated subsegmental pulmonary embolism or inconclusive interpretation of pulmonary embolism using MDCT. *AJR Am J Roentgenol.* 2005;184(2):623-628.

For peer review only - http://bmjopen.bmj.com/site/about/guidelines.xhtml

28. Douketis JD, Foster GA, Crowther MA, Prins MH, Ginsberg JS. Clinical risk factors and timing of recurrent venous thromboembolism during the initial 3 months of anticoagulant therapy. *Arch Intern Med.* 2000;160(22):3431-3436.

29. Pesavento R, de Conti G, Minotto I, et al. The value of 64-detector row computed tomography for the exclusion of pulmonary embolism. *Thromb Haemost.* 2011;105(5):901-907.

30. Raslan IA, Chong J, Gallix B, Lee TC, McDonald EG. Rates of Overtreatment and Treatment-Related Adverse Effects Among Patients With Subsegmental Pulmonary Embolism. *JAMA Intern Med.* 2018;178(9):1272-1274.

31. Yoo HH, Queluz TH, El Dib R. Anticoagulant treatment for subsegmental pulmonary embolism. *Cochrane Database Syst Rev.* 2016(1):CD010222.

32. Chan AW, Tetzlaff JM, Altman DG, et al. SPIRIT 2013 statement: defining standard protocol items for clinical trials. *Ann Intern Med.* 2013;158(3):200-207.

33. Peiman S, Abbasi M, Allameh SF, Asadi Gharabaghi M, Abtahi H, Safavi E. Subsegmental pulmonary embolism: A narrative review. *Thromb Res.* 2016;138:55-60.

34. Schoellnast H, Deutschmann HA, Fritz GA, Stessel U, Schaffler GJ, Tillich M. MDCT angiography of the pulmonary arteries: influence of iodine flow concentration on vessel attenuation and visualization. *AJR Am J Roentgenol.* 2005;184(6):1935-1939.

35. Kearon C. Natural history of venous thromboembolism. *Circulation.* 2003;107(23 Suppl 1):I22-30.

36. Jimenez D, Aujesky D, Diaz G, et al. Prognostic significance of deep vein thrombosis in patients presenting with acute symptomatic pulmonary embolism. *Am J Respir Crit Care Med.* 2010;181(9):983-991.

37. Righini M, Le Gal G, Aujesky D, et al. Complete venous ultrasound in outpatients with suspected pulmonary embolism. *J Thromb Haemost.* 2009;7(3):406-412.

For peer review only

For peer review only - http://bmjopen.bmj.com/site/about/guidelines.xhtml

38. Buller HR, Cohen AT, Davidson B, et al. Idraparinux versus standard therapy for venous thromboembolic disease. *N Engl J Med.* 2007;357(11):1094-1104.

39. Lim W, Le Gal G, Bates SM, et al. American Society of Hematology 2018 guidelines for management of venous thromboembolism: diagnosis of venous thromboembolism. *Blood Adv.* 2018;2(22):3226-3256.

40. Mean M, Righini M, Jaeger K, et al. The Swiss cohort of elderly patients with venous thromboembolism (SWITCO65+): rationale and methodology. *J Thromb Thrombolysis.* 2013;36(4):475-483.

41. Schulman S, Kearon C. Definition of major bleeding in clinical investigations of antihemostatic medicinal products in non-surgical patients. *J Thromb Haemost.* 2005;3(4):692-694.

42. Raskob GE, Spyropoulos AC, Zrubek J, et al. The MARINER trial of rivaroxaban after hospital discharge for medical patients at high risk of VTE. Design, rationale, and clinical implications. *Thromb Haemost.* 2016;115(6):1240-1248.

43. Tritschler T, Kraaijpoel N, Girard P, et al. Definition of pulmonary embolism-related death and classification of the cause of death in venous thromboembolism studies: communication from the SSC of the ISTH. *J Thromb Haemost.* in press.

44. Cohn DM, Nelis EA, Busweiler LA, Kaptein AA, Middeldorp S. Quality of life after pulmonary embolism: the development of the PEmb-QoL questionnaire. *J Thromb Haemost.* 2009;7(6):1044-1046.

45. Klok FA, Cohn DM, Middeldorp S, et al. Quality of life after pulmonary embolism: validation of the PEmb-QoL Questionnaire. *J Thromb Haemost.* 2010;8(3):523-532.

46. Klok FA, Barco S, Siegerink B. Measuring functional limitations after venous thromboembolism: A call to action. *Thromb Res.* 2019;178:59-62.

For peer review only - http://bmjopen.bmj.com/site/about/guidelines.xhtml

47. Aujesky D, Roy PM, Verschuren F, et al. Outpatient versus inpatient treatment for patients with acute pulmonary embolism: an international, open-label, randomised, non-inferiority trial. *Lancet.* 2011;378(9785):41-48.

48. Yealy DM, Auble TE, Stone RA, et al. Effect of increasing the intensity of implementing pneumonia guidelines: a randomized, controlled trial. *Ann Intern Med.* 2005;143(12):881-894.

49. Carrier M, Kimpton M, G LEG, et al. The management of a sub-segmental pulmonary embolism: a cross-sectional survey of Canadian thrombosis physicians. *J Thromb Haemost.* 2011;9(7):1412-1415.

50. Mauri L, D'Agostino RB, Sr. Challenges in the Design and Interpretation of Noninferiority Trials. *N Engl J Med.* 2017;377(14):1357-1367.

51. Levine M, Gent M, Hirsh J, et al. A comparison of low-molecular-weight heparin administered primarily at home with unfractionated heparin administered in the hospital for proximal deep-vein thrombosis. *N Engl J Med.* 1996;334(11):677-681.

52. Koopman MM, Prandoni P, Piovella F, et al. Treatment of venous thrombosis with intravenous unfractionated heparin administered in the hospital as compared with subcutaneous low-molecular-weight heparin administered at home. The Tasman Study Group. *N Engl J Med.* 1996;334(11):682-687.

53. Columbus I, Buller HR, Gent M, et al. Low-molecular-weight heparin in the treatment of patients with venous thromboembolism. *N Engl J Med.* 1997;337(10):657-662.

54. Buller HR, Davidson BL, Decousus H, et al. Fondaparinux or enoxaparin for the initial treatment of symptomatic deep venous thrombosis: a randomized trial. *Ann Intern Med.* 2004;140(11):867-873.

For peer review only

For peer review only - http://bmjopen.bmj.com/site/about/guidelines.xhtml

55. Buller HR, Davidson BL, Decousus H, et al. Subcutaneous fondaparinux versus intravenous unfractionated heparin in the initial treatment of pulmonary embolism. *N Engl J Med.* 2003;349(18):1695-1702.

56. Agnelli G, Buller HR, Cohen A, et al. Oral apixaban for the treatment of acute venous thromboembolism. *N Engl J Med.* 2013;369(9):799-808.

57. Agresti A, Caffo B. Simple and effective confidence intervals for proportions and differences of proportions result from adding two successes and two failures. *The American Statistician.* 2000;54:280-288.

58. Piaggio G, Elbourne DR, Pocock SJ, Evans SJ, Altman DG, Group C. Reporting of noninferiority and equivalence randomized trials: extension of the CONSORT 2010 statement. *JAMA.* 2012;308(24):2594-2604.

59. Fine JP, Gray RJ. Proportional hazards model for the subdistribution of a competing risk. *J Am Stat Assoc.* 1999;94:496-509.

60. van der Hulle T, den Exter PL, Planquette B, et al. Risk of recurrent venous thromboembolism and major hemorrhage in cancer-associated incidental pulmonary embolism among treated and untreated patients: a pooled analysis of 926 patients. *J Thromb Haemost.* 2016;14(1):105-113.

61. Kearon C, Ageno W, Cannegieter SC, et al. Categorization of patients as having provoked or unprovoked venous thromboembolism: guidance from the SSC of ISTH. *J Thromb Haemost.* 2016;14(7):1480-1483.

62. Prandoni P, Noventa F, Ghirarduzzi A, et al. The risk of recurrent venous thromboembolism after discontinuing anticoagulation in patients with acute proximal deep vein thrombosis or pulmonary embolism. A prospective cohort study in 1,626 patients. *Haematologica.* 2007;92(2):199-205.

For peer review only

63. Iorio A, Kearon C, Filippucci E, et al. Risk of recurrence after a first episode of symptomatic venous thromboembolism provoked by a transient risk factor: a systematic review. *Arch Intern Med.* 2010;170(19):1710-1716.

64. van der Hulle T, Tan M, den Exter PL, et al. Recurrence risk after anticoagulant treatment of limited duration for late, second venous thromboembolism. *Haematologica.* 2015;100(2):188-193.

65. Moores LK. Are We Overtreating Isolated Subsegmental Pulmonary Embolism?: First Do No Harm. *JAMA Intern Med.* 2018;178(9):1274-1275.

66. Giugliano RP, Ruff CT, Braunwald E, et al. Edoxaban versus warfarin in patients with atrial fibrillation. *N Engl J Med.* 2013;369(22):2093-2104.

For peer review only

Table 1. Study Population, Intervention, Control, and Outcomes

**Population**	Consecutive adult low-risk patients with an objective diagnosis of **isolated subsegmental PE** who have no concomitant DVT.
**Intervention**	**Clinical surveillance plus a matching rivaroxaban placebo,** one tablet twice daily for the first 21 days, followed by 1 tablet once daily for the remaining 90-da study period. Clinical surveillance is done at 10, 30 and 90 days following randomization by phone or by in-person visits, depending on local practice. At each contact, trained study personnel complete an assessment of symptoms and review for suspected recurrent VTE and bleeding using a checklist of pre-defined questions. Patients are also instructed to contact study personnel or report to the ED immediately if any symptoms/signs compatible with recurrent VTE or significant bleeding occur.
**Control**	**Clinical surveillance plus anticoagulation with rivaroxaban**, dosed at 15 mg twice daily for the first 21 days, followed by 20 mg once daily for the remaining 90-day study period. The same surveillance schedule as in the clinical surveillance group is used. Patients also receive the same instructions to contact study personnel or report to the ED if any signs or symptoms of VTE or significant bleeding occur.
**Outcomes**	Primary outcome: **recurrent, clinically symptomatic, objectively confirmed VTE** within 90 days of randomization, defined as recurrent fatal or nonfatal PE or lower limb DVT (efficacy).<br>Secondary outcomes: **clinically significant bleeding** and **all-cause mortality** at 90 days of randomization (safety).<br>Ancillary outcomes: **health-related quality of life, functional status, and medical resource utilization** at 90 days of randomization.<br>In a post-hoc analysis, **radiological inter-observer agreement** for SSPE will be assessed.

Abbreviations: DVT, deep vein thrombosis; ED, emergency department; PE, pulmonary embolism; SSPE, subsegmental pulmonary embolism; VTE, venous thromboembolism.

For peer review only - http://bmjopen.bmj.com/site/about/guidelines.xhtml

Table 2. Exclusion Criteria and their Rationale

Exclusion Criterion	Rationale
1) Presence of leg DVT or upper extremity DVT (subclavian vein or above)	absolute indication for therapeutic anticoagulation
2) Active cancer	high risk of recurrent VTE if left untreated[60]
3) History of ≥1 prior episode of unprovoked VTE[61]	high risk of recurrent VTE after stopping anticoagulation[62-64]
4) Clinical instability (systolic blood pressure <100 mm Hg or arterial oxygen saturation <92% at ambient air) at the time of presentation	risk of clinical deterioration[4,14,65]
5) Active bleeding or at high risk of bleeding (e.g., signs of active bleeding, ischemic stroke during preceding <10 days,[56] major gastrointestinal bleeding during preceding <3 months, intracranial or intraocular bleeding <6 months,[56] major trauma or surgery during preceding <1 month,[56,66] platelets <75,000 per mm^3,[47] or double anti-platelet therapy at the time of enrolment)	contraindication to rivaroxaban
6) Severe renal failure (creatinine clearance <30 ml/min.)	contraindication to rivaroxaban
7) Severe liver insufficiency (Child-Pugh B and C)	contraindication to rivaroxaban
8) Concomitant use of strong CYP3A4 inhibitors (i.e., HIV protease inhibitors [saquinavir, indinavir, ritonavir, nelfinavir, amprenavir, lopinavir, atazanavir, fosamprenavir, tipranavir, darunavir], systemic azole antifungals [i.e., ketoconazole, itraconazole, voriconazole, or posaconazole]) or strong CYP3A4 inducers (i.e., rifampicin, rifabutin, rifapentin, phenytoin, phenobarbital, primidone, carbamazepine, or St. John's Wort)	contraindication to rivaroxaban
9) Known hypersensitivity to rivaroxaban	contraindication to rivaroxaban
10) Need for therapeutic anticoagulation for another reason (atrial fibrillation/flutter, mechanical heart valves, previous VTE, known antiphospholipid antibody syndrome with unprovoked VTE)	randomization to placebo unethical
11) Therapeutic anticoagulation for >72 hours for any reason at the time of screening	could confound study outcomes
12) Hospitalized for >72 hours prior to the diagnosis of isolated SSPE (hospital-acquired VTE)	could confound study outcomes due to influence of co-treatments
13) Known pregnancy or breast-feeding	contraindication to rivaroxaban
14) Lack of safe contraception in women of childbearing potential	rivaroxaban is contraindicated in pregnancy
15) Refusal or inability to provide informed consent	unethical
16) Prior enrolment in this trial	confounds study outcomes

Abbreviations: DVT, deep vein thrombosis; VTE, venous thromboembolism

For peer review only

For peer review only - http://bmjopen.bmj.com/site/about/guidelines.xhtml

Table 3. Study Schedule

Study Period	Enrolment & Allocation	Baseline	Post-allocation		Close-out
**Visit/Follow-up phone call**	**1**		**2✆****	**3✆****	**4✆****
**Time (day with allowed visit window)**	**d0**	**d0**	**d10 (7-12)**	**d30 (28-35)**	**d90 (88-95)**
Eligibility screen (in-/exclusion criteria)	x				
Patient information and informed consent	x				
Bilateral whole-leg compression ultrasonography *	x				
Randomization	x				
Demographic characteristics		x			
Risk factors for venous thromboembolism		x			
Symptoms of venous thromboembolism		x			
Comorbid conditions		x			
Physical examination findings		x			
Laboratory test results		x			
Imaging findings		x			
Concomitant treatments		x			
Health-related quality of life (PEmb-QoL) †		x		x	x
Functional status ‡					x
Treatment setting		x			
Distribution and instruction of study drug		x			
Daily intake of study medication: placebo or rivaroxaban		x (immediate start on day 0)			
Instructions & distribution of patient diary §		x			
Recurrent venous thromboembolism			x	x	x
Clinically significant bleeding			x	x	x
All-cause mortality			x	x	x
Medical resource utilization			x	x	x
Time to symptom resolution			x	x	x
New concomitant treatments			x	x	x
Interruption of the study drug			x	x	x
Adherence ||					x
Serious adverse events reporting #		x (immediate start after inclusion on day 0)			

* Participants with concomitant deep vein thrombosis are excluded from further study participation (screening failures).

For peer review only

For peer review only - http://bmjopen.bmj.com/site/about/guidelines.xhtml

† At the baseline visit, participants are asked to fill in the PEmb-QoL questionnaire. For follow-up assessments, participants receive the PEmb-QoL including a pre-stamped envelope by mail and are asked to complete and return the questionnaire. If the follow-up interview is done in-person, the PEmb-QoL can be administered during the office visit.

‡ Functional status is assessed using the post-VTE functional status scale

§ The first part of the patient diary consists of an information on the study outline, surveillance interviews, and symptoms and signs suggestive for recurrent venous thromboembolism and bleeding, the investigator's contact information/emergency telephone number if these symptoms/signs occur, how to take the study medications, and instructions to return the drug bottles at the end of the study. The second part consists of a patient diary where patients are asked to record dates and type of outcomes and measures of health resource utilization (e.g., physician visits), and the time to symptom resolution and return to work/usual activities.

|| Adherence is assessed by counting the pill count of the returned medication bottles.

A serious adverse event is defined as any untoward medical occurrence that results in in death, is life-threatening, requires in-patient hospitalization or prolongation of existing hospitalization, results in persistent or significant disability/incapacity, or is a congenital anomaly/birth defect. In addition, important medical events that may jeopardize the patient or may require an intervention to prevent one of these outcomes is also considered serious.

** phone or in-person follow-up, depending on local practice

For peer review only - http://bmjopen.bmj.com/site/about/guidelines.xhtml

Figure. Overview of the Study Design

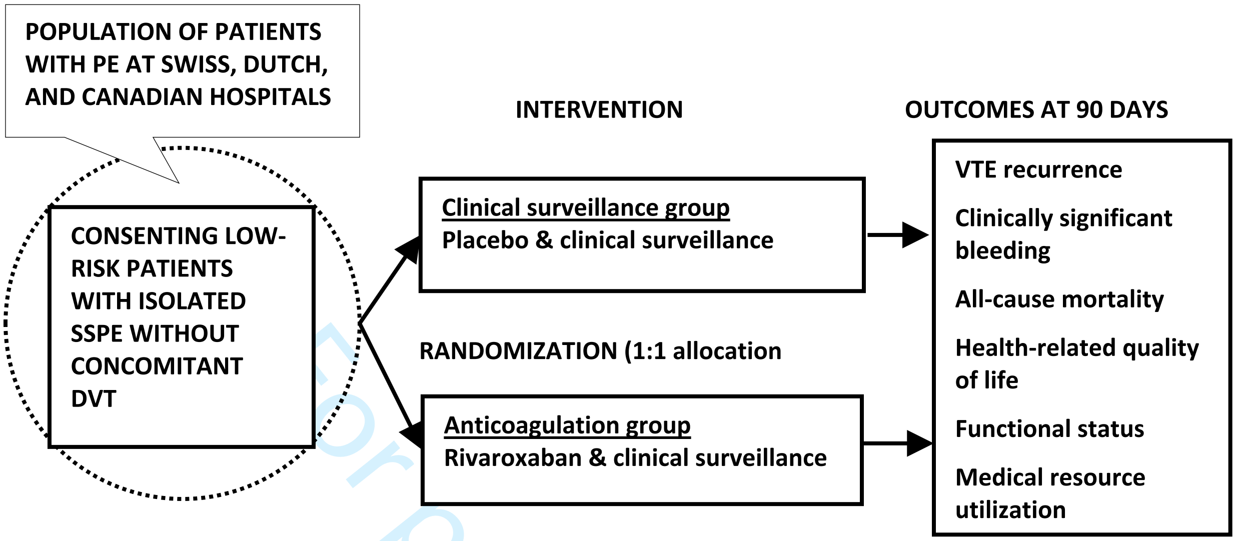

For peer review only - http://bmjopen.bmj.com/site/about/guidelines.xhtml

STANDARD PROTOCOL ITEMS: RECOMMENDATIONS FOR INTERVENTIONAL TRIALS

SPIRIT 2013 Checklist: Recommended items to address in a clinical trial protocol and related documents*

Section/item	Item No	Description	Page No
**Administrative information**			
Title	1	Descriptive title identifying the study design, population, interventions, and, if applicable, trial acronym	1
Trial registration	2a	Trial identifier and registry name. If not yet registered, name of intended registry	4
	2b	All items from the World Health Organization Trial Registration Data Set	4
Protocol version	3	Date and version identifier	4
Funding	4	Sources and types of financial, material, and other support	3
Roles and responsibilities	5a	Names, affiliations, and roles of protocol contributors	1, 21
	5b	Name and contact information for the trial sponsor	22
	5c	Role of study sponsor and funders, if any, in study design; collection, management, analysis, and interpretation of data; writing of the report; and the decision to submit the report for publication, including whether they will have ultimate authority over any of these activities	21
	5d	Composition, roles, and responsibilities of the coordinating centre, steering committee, endpoint adjudication committee, data management team, and other individuals or groups overseeing the trial, if applicable (see Item 21a for data monitoring committee)	21-24
**Introduction**			
Background and rationale	6a	Description of research question and justification for undertaking the trial, including summary of relevant studies (published and unpublished) examining benefits and harms for each intervention	6
	6b	Explanation for choice of comparators	6
Objectives	7	Specific objectives or hypotheses	8

For peer review only

Trial design	8	Description of trial design including type of trial (eg, parallel group, crossover, factorial, single group), allocation ratio, and framework (eg, superiority, equivalence, noninferiority, exploratory)	8
**Methods: Participants, interventions, and outcomes**			
Study setting	9	Description of study settings (eg, community clinic, academic hospital) and list of countries where data will be collected. Reference to where list of study sites can be obtained	8
Eligibility criteria	10	Inclusion and exclusion criteria for participants. If applicable, eligibility criteria for study centres and individuals who will perform the interventions (eg, surgeons, psychotherapists)	9
Interventions	11a	Interventions for each group with sufficient detail to allow replication, including how and when they will be administered	9, 10
	11b	Criteria for discontinuing or modifying allocated interventions for a given trial participant (eg, drug dose change in response to harms, participant request, or improving/worsening disease)	11-12
	11c	Strategies to improve adherence to intervention protocols, and any procedures for monitoring adherence (eg, drug tablet return, laboratory tests)	11
	11d	Relevant concomitant care and interventions that are permitted or prohibited during the trial	11
Outcomes	12	Primary, secondary, and other outcomes, including the specific measurement variable (eg, systolic blood pressure), analysis metric (eg, change from baseline, final value, time to event), method of aggregation (eg, median, proportion), and time point for each outcome. Explanation of the clinical relevance of chosen efficacy and harm outcomes is strongly recommended	12-14
Participant timeline	13	Time schedule of enrolment, interventions (including any run-ins and washouts), assessments, and visits for participants. A schematic diagram is highly recommended (see Figure)	35
Sample size	14	Estimated number of participants needed to achieve study objectives and how it was determined, including clinical and statistical assumptions supporting any sample size calculations	15

For peer review only - http://bmjopen.bmj.com/site/about/guidelines.xhtml

Recruitment	15	Strategies for achieving adequate participant enrolment to reach target sample size	10
**Methods: Assignment of interventions (for controlled trials)**			
Allocation:			
Sequence generation	16a	Method of generating the allocation sequence (eg, computer-generated random numbers), and list of any factors for stratification. To reduce predictability of a random sequence, details of any planned restriction (eg, blocking) should be provided in a separate document that is unavailable to those who enrol participants or assign interventions	8
Allocation concealment mechanism	16b	Mechanism of implementing the allocation sequence (eg, central telephone; sequentially numbered, opaque, sealed envelopes), describing any steps to conceal the sequence until interventions are assigned	8
Implementation	16c	Who will generate the allocation sequence, who will enrol participants, and who will assign participants to interventions	8
Blinding (masking)	17a	Who will be blinded after assignment to interventions (eg, trial participants, care providers, outcome assessors, data analysts), and how	8-9
	17b	If blinded, circumstances under which unblinding is permissible, and procedure for revealing a participant's allocated intervention during the trial	11
**Methods: Data collection, management, and analysis**			
Data collection methods	18a	Plans for assessment and collection of outcome, baseline, and other trial data, including any related processes to promote data quality (eg, duplicate measurements, training of assessors) and a description of study instruments (eg, questionnaires, laboratory tests) along with their reliability and validity, if known. Reference to where data collection forms can be found, if not in the protocol	10, 11
	18b	Plans to promote participant retention and complete follow-up, including list of any outcome data to be collected for participants who discontinue or deviate from intervention protocols	14

For peer review only - http://bmjopen.bmj.com/site/about/guidelines.xhtml

Data management	19	Plans for data entry, coding, security, and storage, including any related processes to promote data quality (eg, double data entry; range checks for data values). Reference to where details of data management procedures can be found, if not in the protocol	11
Statistical methods	20a	Statistical methods for analysing primary and secondary outcomes. Reference to where other details of the statistical analysis plan can be found, if not in the protocol	16
	20b	Methods for any additional analyses (eg, subgroup and adjusted analyses)	16,17
	20c	Definition of analysis population relating to protocol non-adherence (eg, as randomised analysis), and any statistical methods to handle missing data (eg, multiple imputation)	16, 17
**Methods: Monitoring**			
Data monitoring	21a	Composition of data monitoring committee (DMC); summary of its role and reporting structure; statement of whether it is independent from the sponsor and competing interests; and reference to where further details about its charter can be found, if not in the protocol. Alternatively, an explanation of why a DMC is not needed	18
	21b	Description of any interim analyses and stopping guidelines, including who will have access to these interim results and make the final decision to terminate the trial	18
Harms	22	Plans for collecting, assessing, reporting, and managing solicited and spontaneously reported adverse events and other unintended effects of trial interventions or trial conduct	(18), 33
Auditing	23	Frequency and procedures for auditing trial conduct, if any, and whether the process will be independent from investigators and the sponsor	11
**Ethics and dissemination**			
Research ethics approval	24	Plans for seeking research ethics committee/institutional review board (REC/IRB) approval	19

For peer review only - http://bmjopen.bmj.com/site/about/guidelines.xhtml

Protocol amendments	25	Plans for communicating important protocol modifications (eg, changes to eligibility criteria, outcomes, analyses) to relevant parties (eg, investigators, REC/IRBs, trial participants, trial registries, journals, regulators)	19
Consent or assent	26a	Who will obtain informed consent or assent from potential trial participants or authorised surrogates, and how (see Item 32)	10
	26b	Additional consent provisions for collection and use of participant data and biological specimens in ancillary studies, if applicable	n.a.
Confidentiality	27	How personal information about potential and enrolled participants will be collected, shared, and maintained in order to protect confidentiality before, during, and after the trial	19
Declaration of interests	28	Financial and other competing interests for principal investigators for the overall trial and each study site	21
Access to data	29	Statement of who will have access to the final trial dataset, and disclosure of contractual agreements that limit such access for investigators	18
Ancillary and post-trial care	30	Provisions, if any, for ancillary and post-trial care, and for compensation to those who suffer harm from trial participation	n.a.
Dissemination policy	31a	Plans for investigators and sponsor to communicate trial results to participants, healthcare professionals, the public, and other relevant groups (eg, via publication, reporting in results databases, or other data sharing arrangements), including any publication restrictions	19
	31b	Authorship eligibility guidelines and any intended use of professional writers	(22)
	31c	Plans, if any, for granting public access to the full protocol, participant-level dataset, and statistical code	19
**Appendices**			
Informed consent materials	32	Model consent form and other related documentation given to participants and authorised surrogates	
Biological specimens	33	Plans for collection, laboratory evaluation, and storage of biological specimens for genetic or molecular analysis in the current trial and for future use in ancillary studies, if applicable	n/a

For peer review only

*It is strongly recommended that this checklist be read in conjunction with the SPIRIT 2013 Explanation & Elaboration for important clarification on the items. Amendments to the protocol should be tracked and dated. The SPIRIT checklist is copyrighted by the SPIRIT Group under the Creative Commons "Attribution-NonCommercial-NoDerivs 3.0 Unported" license.

For peer review only

BMJ Open

Rationale and Design of Clinical Surveillance vs. Anticoagulation For low-risk patiEnts with isolated SubSegmental Pulmonary Embolism: a multicenter randomized placebo-controlled non-inferiority trial (SAFE-SSPE)

Journal:	*BMJ Open*
Manuscript ID	bmjopen-2020-040151.R1
Article Type:	Protocol
Date Submitted by the Author:	12-Aug-2020

Complete List of Authors:	Baumgartner, Christine; Inselspital University Hospital Bern, General Internal Medicine<br>Klok, Frederikus; Leiden University Medical Center, Department of Thrombosis and Hemostasis<br>Carrier, Marc; Ottawa Hospital Research Institute<br>Limacher, Andreas; Universitat Bern Institut fur Sozial- und Praventivmedizin<br>Moor, Jeanne; Inselspital University Hospital Bern, Department of General Internal Medicine<br>Righini, Marc; Geneva University Hospitals, Division of Angiology and Haemostasis<br>Beer, Jürg-Hans; Kantonsspital Baden AG, Department of Internal Medicine<br>Peluso, Martina; Inselspital University Hospital Bern, Department of General Internal Medicine<br>Rakovic, Damiana; Inselspital University Hospital Bern, Department of General Internal Medicine<br>Huisman, Menno; Leiden University Medical Center, Department of Thrombosis and Hemostasis<br>AUJESKY , Drahomir; Inselspital University Hospital Bern, Department of General Internal Medicine

**Primary Subject Heading**:	Cardiovascular medicine
Secondary Subject Heading:	Emergency medicine, Evidence based practice, Research methods
Keywords:	Anticoagulation < HAEMATOLOGY, Thromboembolism < CARDIOLOGY, Clinical trials < THERAPEUTICS

SCHOLARONE™
Manuscripts

I, the Submitting Author has the right to grant and does grant on behalf of all authors of the Work (as defined in the below author licence), an exclusive licence and/or a non-exclusive licence for contributions from authors who are: i) UK Crown employees; ii) where BMJ has agreed a CC-BY licence shall apply, and/or iii) in accordance with the terms applicable for US Federal Government officers or employees acting as part of their official duties; on a worldwide, perpetual, irrevocable, royalty-free basis to BMJ Publishing Group Ltd ("BMJ") its licensees and where the relevant Journal is co-owned by BMJ to the co-owners of the Journal, to publish the Work in this journal and any other BMJ products and to exploit all rights, as set out in our licence.

The Submitting Author accepts and understands that any supply made under these terms is made by BMJ to the Submitting Author unless you are acting as an employee on behalf of your employer or a postgraduate student of an affiliated institution which is paying any applicable article publishing charge ("APC") for Open Access articles. Where the Submitting Author wishes to make the Work available on an Open Access basis (and intends to pay the relevant APC), the terms of reuse of such Open Access shall be governed by a Creative Commons licence – details of these licences and which Creative Commons licence will apply to this Work are set out in our licence referred to above.

Other than as permitted in any relevant BMJ Author's Self Archiving Policies, I confirm this Work has not been accepted for publication elsewhere, is not being considered for publication elsewhere and does not duplicate material already published. I confirm all authors consent to publication of this Work and authorise the granting of this licence.

For peer review only

Rationale and Design of Clinical Surveillance vs. Anticoagulation For low-risk patiEnts with isolated SubSegmental Pulmonary Embolism: a multicenter randomized placebo-controlled non-inferiority trial (SAFE-SSPE)

Christine Baumgartner, MD, MAS [1]; Frederikus A. Klok, MD, PhD, FESC [2]; Marc Carrier, MD, MSc [3]; Andreas Limacher, PhD [4]; Jeanne Moor, MD [1]; Marc Righini, MD [5]; Jürg-Hans Beer, MD [6]; Martina Peluso [1]; Damiana Rakovic [1]; Menno V. Huisman, MD, PhD [2]; Drahomir Aujesky, MD, MSc [1]

Author Affiliations:

[1] Department of General Internal Medicine, Inselspital, Bern University Hospital, University of Bern, Switzerland

[2] Department of Thrombosis and Hemostasis, Leiden University Medical Center, Leiden, the Netherlands

[3] Department of Medicine, The Ottawa Hospital Research Institute at the University of Ottawa, Ottawa, Canada

[4] CTU Bern, University of Bern, Bern, Switzerland

[5] Division of Angiology and Haemostasis, Geneva University Hospital, University of Geneva, Switzerland

[6] Department of Internal Medicine, Cantonal Hospital of Baden, Baden, Switzerland

Corresponding Author:

Christine Baumgartner, MD, MAS, Department of General Internal Medicine, Inselspital, Bern University Hospital, University of Bern, Freiburgstrasse, 3010 Bern, Switzerland. Phone: +41 (0)31 632 57 69; e-mail: Christine.Baumgartner@insel.ch

Abstract word count: 281, **Text word count:** 4241**, Tables:** 3**, Figures:** 1**, Number of References:** 68

For peer review only - http://bmjopen.bmj.com/site/about/guidelines.xhtml

Keywords: subsegmental pulmonary embolism, anticoagulation, randomized clinical trial

For peer review only

For peer review only - http://bmjopen.bmj.com/site/about/guidelines.xhtml

Abstract

Introduction: The clinical significance of subsegmental pulmonary embolism (SSPE) is currently unclear. Although growing evidence from observational studies suggests that withholding anticoagulant treatment may be a safe option in selected patients with isolated SSPE, most patients with this condition receive anticoagulant treatment, which is associated with a 90-day risk of recurrent VTE of 0.8% and major bleeding of up to 5%. Given the ongoing controversy concerning the risk-benefit ratio of anticoagulation for isolated SSPE and the lack of evidence from randomized-controlled studies, the aim of this clinical trial is to evaluate the efficacy and safety of clinical surveillance without anticoagulation in low-risk patients with isolated SSPE.

Methods and Analysis: SAFE-SSPE (Surveillance vs. Anticoagulation For low-risk patiEnts with isolated SubSegmental Pulmonary Embolism: a multicenter randomized placebo-controlled non-inferiority trial) is an international, multicenter, placebo-controlled, double-blind, parallel-group non-inferiority trial conducted in Switzerland, the Netherlands, and Canada. Low-risk patients with isolated SSPE are randomized to receive clinical surveillance with either placebo (no anticoagulation) or anticoagulant treatment with rivaroxaban. All patients undergo bilateral whole-leg compression ultrasonography to exclude concomitant deep vein thrombosis before enrolment. Patients are followed for 90 days. The primary outcome is symptomatic recurrent VTE (efficacy). The secondary outcomes include clinically significant bleeding and all-cause mortality (safety). The ancillary outcomes are health-related quality of life, functional status, and medical resource utilization.

Ethics and Dissemination: The local Ethics Committees in Switzerland have approved this protocol. Submission to the Ethical Committees in the Netherlands and Canada is underway. The results of this trial will be published in a peer-reviewed journal.

Main Funding: Investigator Initiated Clinical Trials funding scheme of the Swiss National Science Foundation (SNSF). The study medication is provided free of charge by Bayer AG.

For peer review only - http://bmjopen.bmj.com/site/about/guidelines.xhtml

Trial Registration Numbers:

ClinicalTrials.gov: NCT04263038

Protocol version 2.0, 23.01.2020

For peer review only

For peer review only - http://bmjopen.bmj.com/site/about/guidelines.xhtml

Article Summary: Strengths and Limitations of this Study

- This is the first randomized trial comparing the safety and efficacy of a management strategy without anticoagulation and anticoagulant treatment in low-risk patients with isolated subsegmental pulmonary embolism (SSPE).
- We chose patient-centered outcomes and economically relevant efficiency measures that are relevant to both patient and health care professionals.
- If we show non-inferiority, the results of this trial will provide a strong scientific basis for withholding anticoagulation in low-risk patients with SSPE and has the potential to reduce bleeding complications, patient harm, and costs.
- As patients with isolated SSPE and a high risk of adverse events (e.g., patients with cancer) are excluded from this trial due to safety reasons, generalizability of the results will be limited to low-risk patients with isolated SSPE.

For peer review only

Introduction

Depending on thromboembolic burden and patient factors, the clinical spectrum of pulmonary embolism (PE) ranges from asymptomatic cases to massive PE with hemodynamic collapse.[1,2] Anticoagulant treatment for at least 3 months effectively reduces the risk of recurrent venous thromboembolism (VTE).[3 4] The benefit of anticoagulation, however, comes at the cost of potentially disabling and life-threatening bleeding events, with a 90-day risk of major bleeding of up to 5%.[5-8]

The widespread introduction and technological advances of multi-detector computed tomography pulmonary angiography (CTPA) have led to an 80% increase in the diagnosis of acute PE between 1998 and 2006.[9] This increase is in part due to an increase in the detection of small, peripheral PE limited to the subsegmental pulmonary arteries, i.e. subsegmental PE (SSPE),[10,11] which currently comprise 10-15% of cases with PE.[12-15] However, the clinical significance of isolated SSPE is questionable. Epidemiologic evidence suggests overdiagnosis: despite the increase in PE diagnoses in recent years, PE-related mortality has remained stable or has even decraesed.[9,16] The positive predictive value of CTPA to diagnose SSPE is as low as 25% (compared to a composite reference standard with ventilation-perfusion lung scanning with or without lower limb venous ultrasonography, or pulmonary digital-subtraction angiography),[17] and inter-observer agreement between radiologists for SSPE diagnosis is only fair,[18] indicating that differentiation between true emboli and artifacts in the subsegmental pulmonary arteries is difficult. Small PE may even occur in healthy individuals without clinical consequences,[19-21] suggesting that SSPE may be the result from of the physiological filter function of the lung to protect the systemic circulation.

Whether patients with isolated SSPE benefit from anticoagulant treatment is currently uncertain.[4] There is growing evidence from observational studies that withholding anticoagulation may be safe in patients with isolated SSPE who are at low risk of recurrent or progressive VTE,[22-27] but most of such patients currently receive anticoagulant treatment,[28-31] potentially exposing them to an unnecessary risk

For peer review only - http://bmjopen.bmj.com/site/about/guidelines.xhtml

of bleeding. Given the ongoing controversy and clinical equipoise about the risk-benefit ratio of anticoagulation for isolated SSPE, we aim to evaluate the efficacy and safety of clinical surveillance without anticoagulation compared to standard anticoagulation treatment in low-risk patients with SSPE in a randomized clinical trial.

For peer review only

For peer review only - http://bmjopen.bmj.com/site/about/guidelines.xhtml

Methods and Analysis

This study protocol has been developed according to the Standard Protocol Items: Recommendation for Interventional Trials (SPIRIT) guidelines.[32]

Objectives and Hypotheses

The primary objective of this randomized trial is to compare the frequency of symptomatic, recurrent VTE in low-risk patients with isolated SSPE randomized to receive clinical surveillance plus placebo or clinical surveillance plus anticoagulant treatment with rivaroxaban. As a secondary objective, the frequency of clinically significant bleeding and all-cause mortality is compared in the two groups. Ancillary endpoints include health-related quality of life, functional status, and medical resource utilization. We hypothesize that clinical surveillance without anticoagulant treatment is non-inferior to anticoagulation in terms of recurrent VTE, while resulting in fewer clinically significant bleeding events and similar all-cause mortality. We also hypothesize that clinical surveillance, compared to anticoagulation, improves health-related quality of life and functional status and reduces medical resource utilization.

Study Design and Setting

SAFE-SSPE (clinical Surveillance vs. Anticoagulation For low-risk patiEnts with isolated SubSegmental Pulmonary Embolism) is an investigator-initiated, multicenter, randomized, placebo-controlled, double-blind, parallel-group non-inferiority trial. Patients with isolated SSPE at low risk for VTE progression or recurrence and without concomitant deep vein thrombosis (DVT) are randomly assigned in a 1:1 ratio to receive clinical surveillance plus placebo or clinical surveillance plus anticoagulation with rivaroxaban (**Figure**). Randomization is blocked and stratified by study site. To ensure concealment of allocation, the allocation sequence is generated by a data manager not involved in the study, using a computer-generated randomization schedule. All participants, care providers,

For peer review only - http://bmjopen.bmj.com/site/about/guidelines.xhtml

investigators, study personnel, members of the outcomes adjudication committee, data management personnel, and analysts are blinded to group assignment. Eligible patients are recruited in at least 27 university and medium- to high-volume non-university teaching hospitals in Switzerland, the Netherlands, and Canada.

Selection of Patients

Consecutive patients aged ≥18 years with objectively diagnosed symptomatic or asymptomatic isolated SSPE on CTPA based on the assessment of the local radiologist at the time of patient presentation are eligible for study participation after provision of informed consent (**Table 1**). Isolated SSPE is defined as multi-detector CTPA demonstrating an intraluminal filling defect in ≥1 subsegmental pulmonary artery (4th order or higher) without filling defects visualized at more proximal pulmonary artery levels.[24,33,34] Isolated subsegmental defects are classified as either single (1 subsegmental vessel involved) or multiple (≥2 subsegmental vessels involved). Patients with both symptomatic and incidentally detected, asymptomatic isolated SSPE are potentially eligible because symptoms of PE may be subtle and difficult to elucidate, and symptomatic and asymptomatic PE appear to have a similar prognosis.[35] We will enroll both patients with single and multiple isolated SSPE because there is no convincing evidence that these conditions are prognostically different.

The exclusion criteria were selected based on a high early risk of VTE recurrence (≥8%), the presence of cardiopulmonary compromise (i.e. hypotension or hypoxemia), contraindications to anticoagulant treatment with rivaroxaban, and a high risk of confounding (**Table 2**). Because PE usually originates from a thrombus in the deep leg veins,[45] potentially eligible patients systematically receive a single bilateral whole-leg compression ultrasonography (CUS) examination to exclude concomitant DVT, which is a well-known prognostic factor for mortality in patients with PE and represents an absolute indication for therapeutic anticoagulation.[46] If a proximal or distal DVT (with an incompressible distal

For peer review only - http://bmjopen.bmj.com/site/about/guidelines.xhtml

vein diameter of ≥5mm)[47] is detected, patients are excluded from study participation and treated at the discretion of their managing physician. All exclusion criteria are listed in **Table 2**.

Intervention

Patients in the intervention group receive clinical surveillance and a matching rivaroxaban placebo orally using the same dosing schedule, frequency of administration, and duration of treatment as for rivaroxaban (see below). Clinical surveillance is done during the follow-up interviews by phone or in-person, depending on local practice. At each contact, trained study personnel complete an assessment of symptoms and review for suspected recurrent VTE and bleeding. If patients report symptoms or signs suggestive of recurrent VTE or significant bleeding, they are asked to present immediately to an ED for evaluation.

Control

Patients who are assigned to the anticoagulation group receive oral rivaroxaban 15 mg twice daily for the first 21 days, followed by 20 mg once daily for the remaining 90-day study period. After completion of the treatment period, the study drug is discontinued. The same surveillance schedule as in the intervention group is used.

Study Procedures

Patients are screened for eligibility if they receive a CTPA in the emergency department or within 72 hours of hospitalization to rule-in or rule-out PE, or if they present to an outpatient service within 72 hours of SSPE diagnosis. Eligibility criteria are assessed for all consecutive patients with suspected SSPE, and potentially eligible patients are asked to provide informed consent by investigators or their delegates. For those who consent to participate, a bilateral whole-leg CUS is performed by qualified

For peer review only - http://bmjopen.bmj.com/site/about/guidelines.xhtml

examiners (e.g. radiologists, vascular specialists, or emergency physicians). All eligible and consenting patients without DVT are randomized to receive clinical surveillance or anticoagulation with rivaroxaban, and trained study personnel collect baseline data by reviewing medical records, medication lists, laboratory results that have been obtained as part of routine care, radiology reports, and by patient interview (**Table 3**). After provision of the assigned study medication, the participants are asked to immediately start with the treatment. In addition, all participants receive a patient diary consisting of two parts: the first contains important information about the study, and the second part consists of a diary to record dates and type of outcomes in order to minimize recall bias (**Table 3**).

Participants are followed for 90 days using phone or in-person interviews at 10, 30, and 90 days after randomization (Supplementary File). Trained study personnel contacts patients, family members, and/or primary care physicians and review medical charts to obtain information about outcome events. At the end of the treatment period, patients are instructed to return the medication bottles and the patient diary. Drug-adherence is assessed by counting the pills in the returned medication bottles (**Table 3**).

Data Collection and Quality

Data are collected using an electronic database (secuTrial®). The following measures are implemented to ensure optimal data quality and completeness: 1) training of study personnel in the methods of data abstraction, patient inquiry, and data recording, 2) recording of study data on standardized electronic case report forms (eCRFs), 3) operations manual providing information on definitions and acceptable data sources for all variables, 4) central data monitoring with generation of statistical reports and individual data checks, and 5) risk based on-site monitoring (i.e., based on key performance indicators such as inappropriate recruitment rate, change of principal investigator, high number of queries raised by central data monitoring, high number of protocol deviations, etc.).

For peer review only - http://bmjopen.bmj.com/site/about/guidelines.xhtml

Criteria for Discontinuation of the Study Medication and Unblinding

In participants requiring prophylactic or therapeutic anticoagulation during the study period for reasons other than the index SSPE or if treatment with another prohibited agent is necessary (i.e. strong CYP3A4 inhibitors or inducers, dual antiplatelet therapy, GP IIb/IIIa inhibitors), the study drug should be temporarily interrupted and restarted as soon as possible following discontinuation of the prohibited medication. Patients who develop any condition requiring permanent therapeutic anticoagulation (e.g., atrial fibrillation) should permanently discontinue the study drug. If an invasive procedure or surgical intervention is required, the study medication should be stopped at least 24 hours prior to an elective intervention, or immediately for emergency procedures. If objectively confirmed recurrent VTE or pregnancy is diagnosed during the treatment period, the study drug should be discontinued and treatment allocation unblinded. Similarly, in case of major bleeding, the study drug should be stopped, and unblinding may be necessary if emergency anticoagulation reversal is indicated.

Primary and Secondary Outcomes

The primary (efficacy) outcome is the proportion of recurrent, clinically symptomatic, objectively confirmed VTE within 90 days of randomization, defined as recurrent PE or lower limb DVT.[48,49] The objective diagnostic criterion for PE, based on available radiographic reports, is a new intraluminal filling defect on CTPA or pulmonary angiography; a perfusion defect involving at least 75% of a segment, with corresponding normal ventilation (i.e., high probability lung scan); the confirmation of new PE at autopsy; or objectively confirmed proximal DVT of the lower extremity in patients with symptoms of PE. The objective diagnosis of DVT is the non-compressibility of a venous segment on CUS or an intraluminal filling defect on contrast venography. Because compression of iliac veins and the inferior vena cava may be technically difficult, additional diagnostic criteria for iliac and caval DVT also include abnormal duplex

For peer review only - http://bmjopen.bmj.com/site/about/guidelines.xhtml

flow patterns compatible with thrombosis or an intraluminal filling defect on computed tomography or magnetic resonance imaging venography.[50] Both proximal and distal DVTs are considered.

Separate secondary (safety) outcomes include the proportion of clinically significant bleeding and all-cause mortality 90 days following randomization. Clinically significant bleeding is a composite endpoint of major and clinically relevant non-major bleeding. Major bleeding is defined as fatal bleeding, symptomatic bleeding at critical sites (intracranial, intraspinal, intraocular, retroperitoneal, intraarticular, pericardial, or intramuscular with compartment syndrome), or bleeding with a reduction of hemoglobin ≥20 g/L, or bleeding leading to transfusion of ≥2 units of packed red blood cells according to the definition of the International Society on Thrombosis and Haemostasis.[51] Clinically relevant non-major bleeding is defined as overt bleeding that does not meet criteria for major bleeding but is associated with a medical intervention, unscheduled physician contact (visit or telephone call), temporary cessation of the study drug, pain, or impairment of activities of daily life.[52]

Information about the date, type, and circumstances of outcome events is obtained from patients, family members, and/or health care providers during the follow-up interviews or by reviewing medical charts. In patients who experience recurrent VTE, the radiographic report and images confirming VTE recurrence are obtained. For patients who died during follow-up, the cause of death based on medical reports, death certificates, and autopsy reports (if available) is recorded. All medical outcome events are reviewed and adjudicated by a committee of 3 independent clinical experts unaware of treatment assignment. Based on available information, death is adjudicated as PE-related, due to major bleeding (any death following an intracranial hemorrhage or a bleeding episode leading to hemodynamic deterioration), due to another cause, or due to an undetermined cause. Death is considered PE-related in the following situations: a) autopsy-confirmed PE in the absence of another more likely cause of death, b) objectively confirmed PE within the last 48 hours before death in the absence of another more likely cause of death, or c) PE is not objectively confirmed, but is most likely the

For peer review only - http://bmjopen.bmj.com/site/about/guidelines.xhtml

main cause of death.[53] Final classification of all medical outcomes is based on the full consensus of the committee.

Ancillary Outcomes

Ancillary outcomes include health-related quality of life and functional status, both important patient-centered outcomes, and medical resource utilization, an economically relevant efficiency measure. Disease-specific, health-related quality of life at baseline, 30 and 90 days after the index PE is assessed using the Pulmonary Embolism Quality of Life (PEmb-QoL) questionnaire.[54,55] The PEmb-QoL is a validated, self-administered 40-item questionnaire to quantify quality of life in patients having experienced PE. Functional status is measured at 90 days after randomization using the post-VTE functional status scale.[56,57] This scale has been proposed to assess functional limitations after VTE, covering aspects of daily life (including limitations in usual activity and changes in lifestyle) that are affected by the consequences of VTE and its complications. The assessment is done using a short, structured interview 90 days after randomization.

The following measures of medical resource utilization and productivity are assessed:[44,58] initial length of stay (LOS), subsequent overall hospitalizations as well as overall ED and physician outpatient visits within 90 days of randomization, and time to return to work in workers or usual activities (e.g., household) in non-workers. Information on these outcomes are obtained from the participant during the follow-up interviews, by interview of the patient's primary care physician, and by hospital chart review. Subsequent health care contacts are classified as potentially related to VTE if a patient had chest or leg symptoms or signs (dyspnea, chest pain, pleural effusion, or leg pain or swelling), or bleeding complications.

Withdrawals and Loss to Follow-Up

For peer review only - http://bmjopen.bmj.com/site/about/guidelines.xhtml

Given the low treatment and follow-up burden and the short follow-up period of 90 days, we expect that completeness of follow-up data collection will be close to 100% based on prior experience.[44] A patient who withdraws consent for follow-up at 10 or 30 days may still agree with passive follow-up (i.e. that study personnel may collect follow-up information from medical records of the participant's primary care physician) or to continue with the assessment at 90 days, if given the option. If study withdrawal occurs, data collected up to the time of withdrawal is used in a coded manner. If a participant is lost to follow-up, primary care physicians and surrogates are contacted, and the hospital records are consulted to obtain information about primary and secondary outcomes and survival status.

Post-hoc Evaluation of Radiologic Inter-Observer Agreement

CTPA images undergo central review at the Bern University Hospital by a panel of two experienced thoracic radiologists blinded to the interpretation of the radiologist at the enrolling site. Based on the consensus of this panel, the initial CTPA readings are classified into 3 categories: 1) presence of isolated SSPE (true-positive isolated SSPE), 2) absence of any PE (false-positive isolated SSPE), and 3) presence of segmental, lobar, or central PE (false-negative isolated SSPE). The panel also evaluates the technical quality of the CTPA examination (adequacy of opacification, breathing artifacts) and the number of filling defects (i.e., single vs. multiple isolated SSPE). Confirmation of the SSPE diagnosis prior to enrolling the patient is logistically not feasible due to the short timeline between diagnosis and enrolment, and it would not reflect real-world practice, thus limiting the external validity of our study results. Therefore, the central review of CTPA images is done post-hoc in 6-month batches.

Sample Size Calculation

Assumptions on VTE recurrence risk are based on data from 127 low-risk patients with isolated SSPE who received anticoagulants (warfarin or low-molecular-weight heparin), showing a VTE recurrence

For peer review only - http://bmjopen.bmj.com/site/about/guidelines.xhtml

risk of 0.8% at 90 days after diagnosis.[23] We chose an absolute non-inferiority margin of 3.5% on the basis of recruitment feasibility, clinical acceptability, and previous studies. This corresponds to a difference which is considered acceptable by most physicians and patients for the following reasons. First, our margin is within the range of the 3-month VTE recurrence proportion (0.5-5%) below which thrombosis specialists would not initiate anticoagulation for PE.[59] Second, the definition of a clinically acceptable non-inferiority margin for recurrent VTE must also take into account the potential benefits of withholding anticoagulation, i.e. the substantially lower risk of clinically significant bleeding (<1% versus 7% within 3 months for patients receiving anticoagulants).[5,23,25,60] Indeed, a patient group with PE who was involved in the trial planning process indicated that given the bleeding risk associated with anticoagulants, a VTE recurrence proportion of <5% seemed acceptable. Finally, similar non-inferiority margins (3-5%) have been used in key studies comparing different drug treatment regimens and inpatient vs. outpatient management for acute VTE.[42,44,61-65]

To determine the sample size, we used a Monte-Carlo simulation approach based on an Agresti-Caffo confidence interval (CI) for risk difference.[66] Assuming a baseline VTE recurrence proportion of 1.0% at 90 days in both treatment groups, an absolute margin of 3.5% defining non-inferiority for clinical surveillance, and a sampling ratio of 1:1 allowing 5% attrition (dropouts, including patients who died from non-VTE-related causes) in each group during 90 days, we estimated that 276 patients (138 per group) would result in at least 80% power to establish non-inferiority at an one-sided type I error of 5%.

Planned Statistical Analyses

As recommended by the CONSORT statement for non-inferiority trials,[67] we will perform both intention-to-treat (ITT) and per-protocol (PP) analyses. For the primary outcome, ITT and PP analyses should reach the same conclusion to consider results to be robust. For the remaining outcomes, the ITT analysis will be the primary analysis, the PP analysis a secondary analysis.

For peer review only - http://bmjopen.bmj.com/site/about/guidelines.xhtml

In the ITT analysis, all randomized patients will be analyzed within the treatment group to which they were randomized. In the PP analysis, patients with protocol violations will be excluded (crossover to study treatment from other group; patients receiving a different type or dose of anticoagulation than requested by the study protocol; patients with missing primary outcome data; patients violating relevant eligibility criteria; patients stopping study treatment within 1 month after randomization, or patients in whom the diagnosis of isolated SSPE was refuted by the central CTPA review panel).

We will describe the prevalence of recurrent VTE, clinically significant bleeding (including its individual components, major and clinically relevant non-major bleeding), and all-cause mortality at 90 days after randomization with 95% Wilson CIs by treatment group. Non-inferiority of the primary outcome (VTE recurrence) among patients in the clinical surveillance vs. the anticoagulation group will be assessed based on the Agresti-Caffo CI for risk difference.[66] If the upper limit of the one-sided 95% Agresti-Caffo CI will be lower than the pre-specified non-inferiority margin, clinical surveillance will be considered non-inferior to anticoagulation. For secondary outcomes, we will calculate the risk difference with a two-sided 95% Agresti-Caffo CI and compare groups using an exact binomial test.[66]

PEmb-QoL dimension scores at 30 and 90 days as well as the change in scores from baseline will be presented by treatment group as means with 95% CIs. The change in dimension scores and the differences in the change between groups will be analyzed using a repeated-measures, linear mixed-effects model adjusted for the respective baseline value. Functional status, which is measured on a scale from 0 (no functional limitations) to 5 (death) at 90 days, will be presented as median and interquartile range and compared between groups using the non-parametric Wilcoxon rank-sum test. Count data (subsequent hospitalizations, outpatient visits) will be presented by treatment group as rate with an exact 95% Poisson CI and compared using a rate ratio and exact p-value. Time-to-event outcomes (initial LOS, time to return to work/usual activities) will be presented as medians and interquartile ranges. For LOS, we will compare groups using the Wilcoxon rank-sum test. For return to work, we will display

For peer review only - http://bmjopen.bmj.com/site/about/guidelines.xhtml

Kaplan-Meier curves and compare groups by the log-rank test. An alpha level of <0.05 will define statistical significance.

In secondary analyses, we will calculate the cumulative incidence and the difference in cumulative incidence of VTE recurrence and clinically significant bleeding, correcting for withdrawals and losses to follow-up by censoring and for death unrelated to VTE recurrence or bleeding as a competing event. All-cause mortality will be assessed likewise, however a competing event does not apply. In a further secondary analysis, we will use model-based approaches for all outcomes. For time-to-event outcomes (VTE recurrence, clinically significant bleeding, LOS, return to work/usual activity), we will use competing risk regression according to Fine and Gray,[68] accounting for non-VTE/non-bleeding-related death or all-cause death as a competing event. For mortality, we will use Cox regression. For count data, we will use a negative binomial model, and also consider zero-inflation. In case of heterogeneity across sites, we will adjust models for site using random-effects models.

Data Monitoring and Interim Safety Analysis

An independent Data and Safety Monitoring Board (DSMB) consisting of 3 members unaffiliated with any of the participating institutions will evaluate unblinded interim safety results after 100 and 190 randomized patients have completed the 90-day follow-up. To monitor recurrent VTE in the intervention group, formal interim analyses will be performed using a Bayesian approach. Based on literature,[23] we expect a frequency of VTE recurrence of 1.0% (0-4.5%). We regard a frequency of >5% in the surveillance group as clinically unacceptable. We will calculate the prior probability distribution of events based on the expected frequency as well as the posterior probability distribution using actually observed data at each interim analysis. Based on the posterior distribution, we will calculate the probability that the true proportion of VTE recurrence in the surveillance group exceeds the threshold of unacceptable frequency. If the probability of exceedance is >70%, the surveillance arm will be considered inferior and the DSMB

For peer review only - http://bmjopen.bmj.com/site/about/guidelines.xhtml

will recommend to stop the trial. The final decision will lie with the Steering Committee. This procedure is conservative regarding the type-I error rate; therefore, we will not adjust the significance level in the final analysis.

Patient and Public Involvement

We have partnered with a group of patients who had recently experienced PE in the planning process of this trial, including the selection of patient-centered outcomes, the establishment of a safe surveillance schedule, and the determination of a clinically acceptable non-inferiority margin. Patients are not involved in the recruitment and conduct of the study or the dissemination of study results.

Data Sharing

After publication of the study results, a de-identified patient-level data set relating to the primary publication along with the latest version of the study protocol, the informed consent form, the statistical analysis plan, the analysis code, and the data management plan of the study will be made publicly available in the *Bern Open Repository and Information System* (*BORIS) Research Data*.

Ethics and Dissemination

This trial is conducted in accordance with the Declaration of Helsinki, the International Council on Harmonization Good Clinical Practice (ICH-GCP) guidelines, and all applicable national legal and regulatory requirements. Authorization by the local Ethics Committees and Swissmedic has been obtained in Switzerland, and the submission process to the relevant Dutch and Canadian Ethics Committees and regulatory authorities is ongoing. All changes in research activity or unanticipated problems involving risks to human subjects will be reported promptly to the Ethics Committees. All participating investigators/institutions will permit study-related monitoring, audits, Ethics Committee

For peer review only - http://bmjopen.bmj.com/site/about/guidelines.xhtml

reviews, and regulatory inspections, and will provide direct access to source documents and data. Protection of confidentiality is ensured according to regulatory requirements.

Study personnel informs eligible patients with a diagnosis of isolated SSPE about all aspects of the study participation, including the goals, procedures, and potential risks/benefits associated with the study. Potential participants are informed that the decision to participate in the study is entirely voluntary and that they may withdraw from the study at any time, with no effect on their current or future treatment. Written informed consent is obtained from all eligible patients prior to any study-related procedure, including bilateral whole-leg CUS. The primary study results will be presented at scientific conferences and published in a peer-reviewed medical journal. We also plan local presentations for physicians at the participating sites. Participants will receive a letter with the study results explained in lay language. Further public dissemination of the results is planned through publications in the lay press and via social media.

Conclusion

The SAFE-SSPE trial addresses an important gap of knowledge, the optimal management of SSPE, and represents the first randomized, direct comparison of clinical surveillance alone versus anticoagulant treatment for low-risk patients with isolated SSPE. As the number of SSPE patients who may eventually receive anticoagulant treatment is likely to rise in the future with the further dissemination and advancement of CTPA technology, a strong scientific basis for withholding anticoagulation in low risk patients with SSPE is urgently needed. The results of this trial have the potential to improve quality and efficiency of care by reducing bleeding episodes and resource utilization and increasing health-related quality of life.

For peer review only - http://bmjopen.bmj.com/site/about/guidelines.xhtml

Current Status of the SAFE-SSPE trial

Patient recruitment has started in May 2020; by July 2020, 6 patients have been enrolled in the trial.

Follow-up of the last participant is expected to be completed in February 2024.

For peer review only

For peer review only - http://bmjopen.bmj.com/site/about/guidelines.xhtml

Authors' Contributions

Study concept and design: DA, MH, MC, FAK, JHB, MR, AL, CB

Drafting of the manuscript: CB, DA

Critical revision of the manuscript for important intellectual content: MH, MC, FAK, JHB, MR, AL, JM, MP, DR

Statistical analysis plan: AL

Obtained funding: DA, MR, JHB

Administrative, technical, or material support: MP, JM, DR, CB

Supervision: DA

Acknowledgements

We would like to thank Sven Trelle, Director of the CTU Bern (Clinical Trials Unit) for his methodological inputs and organizational work concerning this study.

Funding Statement

This study is supported by an Investigator Initiated Clinical Trials (IICT) grant of the Swiss National Science Foundation (SNSF 33IC30_185616). The study medication was provided free of charge by Bayer AG.

Competing Interests Statement

Dr. Carrier reports grants from Leo Pharma, BMS, Pfizer, personal fees from Bayer, BMS, Pfizer, Sanofi, Leo Pharma, Servier, outside the submitted work. All other authors declare that they have no competing interest with regard to the intellectual concern and proprietary of affairs. The funding source had no role in the design of this study and does not have any role in the conduct, the collection, management, analysis, and interpretation of the data, the writing of the manuscript, or the decision to submit the results for

For peer review only

For peer review only - http://bmjopen.bmj.com/site/about/guidelines.xhtml

publication.

Conveyance and Peer Review

This trial protocol has been peer-reviewed for approval of funding and ethical approval prior to submission.

Roles and Responsibilities

Sponsor-Investigator:

Prof. Drahomir Aujesky, MD, MSc, Department of General Internal Medicine, Inselspital, Bern University Hospital, University of Bern, Freiburgstrasse, CH-3010 Bern, Switzerland. Phone: +41 (0)31 632 88 84; e-mail: drahomir.aujesky@insel.ch

Role: sponsor-investigator, coordinating principal investigator, chair of the Steering Committee; responsible for the study design, overseeing the management of the study, interpretation of the data, and writing of the manuscript.

Legal Representative of the Sponsor in the Netherlands:

Frederikus A. Klok, MD, PhD, FESC, Leiden University Medical Center, Albinusdreef 2, NL-2333 ZA Leiden, The Netherlands. Phone: +31 71 529 8127; e-mail: F.A.Klok@lumc.nl

role: Steering Committee member, venous thromboembolism expert, site investigator, recruitment, legal representative of the sponsor in the Netherlands

Legal representative of the Sponsor in Canada:

Prof. Marc Carrier, MD, MSc, Department of Medicine, The Ottawa Hospital Research Institute at the University of Ottawa, CA-Ottawa, K1H8L6, Canada. Phone: +1 613 737 8899, e-mail: mcarrier@toh.ca

role: Steering Committee member, venous thromboembolism expert, site investigator, recruitment, legal representative of the sponsor in Canada

For peer review only

Steering Committee:

Chair: Prof. Drahomir Aujesky, MD, MSc

Steering committee members: Prof. Jürg-Hans Beer, MD; Prof. Marc Carrier, MD, MSc; Dr. Frederikus A. Klok, MD, PhD, FESC; Prof. Marc Righini, MD

The study steering committee is responsible for the overall supervision of the trial and regularly meets to discuss scientific and logistic aspects of the trial.

Monitoring Institution and Data Management:

CTU Bern (Clinical Trials Unit), University of Bern, Mittelstrasse 43, CH-3012 Bern, Switzerland. Phone: +41 (0)31 631 35 04; e-mail: sven.trelle@ctu.unibe.ch

Date Safety Monitoring Board:

An independent Data Safety Monitoring Board (DSMB) evaluates unblinded VTE outcomes on a regular basis and make recommendations to the Steering Committee to amend or terminate the trial should any safety concerns arise. The DSMB includes 3 members unaffiliated with Bern University Hospital or any research partners: 2 experts in the area of VTE, and 1 expert in statistics and methodology of randomized trials.

Other Relevant Committees and Institutions:

The study is managed and coordinated at the Inselspital, Bern University Hospital.

An independent outcome adjudication committee consisting of 3 clinical experts at the Inselspital, Bern University Hospital, is responsible for adjudication of the primary and secondary outcomes events in a blinded fashion.

For peer review only - http://bmjopen.bmj.com/site/about/guidelines.xhtml

A central CT review panel, consisting of two experienced thoracic radiologists at the Inselspital, Bern, University Hospital, centrally reviews all CTPA images.

The Hospital Pharmacy of the Inselspital, Bern University Hospital, is in charge of the pharmaceutical tasks.

For peer review only

For peer review only - http://bmjopen.bmj.com/site/about/guidelines.xhtml

References

1. Di Nisio M, van Es N, Buller HR. Deep vein thrombosis and pulmonary embolism. *Lancet.* 2016;388(10063):3060-3073.

2. Huisman MV, Barco S, Cannegieter SC, et al. Pulmonary embolism. *Nat Rev Dis Primers.* 2018;4:18028.

3. Castellucci LA, Cameron C, Le Gal G, et al. Clinical and safety outcomes associated with treatment of acute venous thromboembolism: a systematic review and meta-analysis. *JAMA.* 2014;312(11):1122-1135.

4. Kearon C, Akl EA, Ornelas J, et al. Antithrombotic Therapy for VTE Disease: CHEST Guideline and Expert Panel Report. *Chest.* 2016;149(2):315-352.

5. Buller HR, Prins MH, Lensin AW, et al. Oral rivaroxaban for the treatment of symptomatic pulmonary embolism. *N Engl J Med.* 2012;366(14):1287-1297.

6. Jun M, Lix LM, Durand M, et al. Comparative safety of direct oral anticoagulants and warfarin in venous thromboembolism: multicentre, population based, observational study. *Bmj.* 2017;359:j4323.

7. Linkins LA, Choi PT, Douketis JD. Clinical impact of bleeding in patients taking oral anticoagulant therapy for venous thromboembolism: a meta-analysis. *Ann Intern Med.* 2003;139(11):893-900.

8. Scherz N, Mean M, Limacher A, et al. Prospective, multicenter validation of prediction scores for major bleeding in elderly patients with venous thromboembolism. *J Thromb Haemost.* 2013;11(3):435-443.

9. Wiener RS, Schwartz LM, Woloshin S. Time trends in pulmonary embolism in the United States: evidence of overdiagnosis. *Arch Intern Med.* 2011;171(9):831-837.

For peer review only

For peer review only - http://bmjopen.bmj.com/site/about/guidelines.xhtml

10. Auer RC, Schulman AR, Tuorto S, et al. Use of helical CT is associated with an increased incidence of postoperative pulmonary emboli in cancer patients with no change in the number of fatal pulmonary emboli. *J Am Coll Surg.* 2009;208(5):871-878; discussion 878-880.

11. Ikesaka R, Carrier M. Clinical significance and management of subsegmental pulmonary embolism. *J Thromb Thrombolysis.* 2015;39(3):311-314.

12. Carrier M, Righini M, Wells PS, et al. Subsegmental pulmonary embolism diagnosed by computed tomography: incidence and clinical implications. A systematic review and meta-analysis of the management outcome studies. *J Thromb Haemost.* 2010;8(8):1716-1722.

13. Carrier M, Righini M, Le Gal G. Symptomatic subsegmental pulmonary embolism: what is the next step? *J Thromb Haemost.* 2012;10(8):1486-1490.

14. Stein PD, Goodman LR, Hull RD, Dalen JE, Matta F. Diagnosis and management of isolated subsegmental pulmonary embolism: review and assessment of the options. *Clin Appl Thromb Hemost.* 2012;18(1):20-26.

15. van der Pol LM, Bistervels IM, van Mens TE, et al. Lower prevalence of subsegmental pulmonary embolism after application of the YEARS diagnostic algorithm. *Br J Haematol.* 2018.

16. Barco S, Mahmoudpour SH, Valerio L, et al. Trends in mortality related to pulmonary embolism in the European Region, 2000-15: analysis of vital registration data from the WHO Mortality Database. *Lancet Respir Med.* 2020;8(3):277-287.

17. Stein PD, Fowler SE, Goodman LR, et al. Multidetector computed tomography for acute pulmonary embolism. *N Engl J Med.* 2006;354(22):2317-2327.

18. Ghanima W, Nielssen BE, Holmen LO, Witwit A, Al-Ashtari A, Sandset PM. Multidetector computed tomography (MDCT) in the diagnosis of pulmonary embolism: interobserver agreement among radiologists with varied levels of experience. *Acta Radiol.* 2007;48(2):165-170.

For peer review only - http://bmjopen.bmj.com/site/about/guidelines.xhtml

19. Gurney JW. No fooling around: direct visualization of pulmonary embolism. *Radiology.* 1993;188(3):618-619.

20. Schoepf UJ, Costello P. CT angiography for diagnosis of pulmonary embolism: state of the art. *Radiology.* 2004;230(2):329-337.

21. Carrier M, Klok FA. Symptomatic subsegmental pulmonary embolism: to treat or not to treat? *Hematology Am Soc Hematol Educ Program.* 2017;2017(1):237-241.

22. Cha SI, Shin KM, Lee JW, et al. Clinical characteristics of patients with peripheral pulmonary embolism. *Respiration.* 2010;80(6):500-508.

23. Donato AA, Khoche S, Santora J, Wagner B. Clinical outcomes in patients with isolated subsegmental pulmonary emboli diagnosed by multidetector CT pulmonary angiography. *Thromb Res.* 2010;126(4):e266-270.

24. Le Gal G, Righini M, Parent F, van Strijen M, Couturaud F. Diagnosis and management of subsegmental pulmonary embolism. *J Thromb Haemost.* 2006;4(4):724-731.

25. Mehta D, Barnett M, Zhou L, et al. Management and outcomes of single subsegmental pulmonary embolus: a retrospective audit at North Shore Hospital, New Zealand. *Intern Med J.* 2014;44(9):872-876.

26. Bariteau A, Stewart LK, Emmett TW, Kline JA. Systematic review and meta-analysis of outcomes of patients with subsegmental pulmonary embolism with and without anticoagulation treatment. *Acad Emerg Med.* 2018.

27. Eyer BA, Goodman LR, Washington L. Clinicians' response to radiologists' reports of isolated subsegmental pulmonary embolism or inconclusive interpretation of pulmonary embolism using MDCT. *AJR Am J Roentgenol.* 2005;184(2):623-628.

For peer review only

For peer review only - http://bmjopen.bmj.com/site/about/guidelines.xhtml

28. Douketis JD, Foster GA, Crowther MA, Prins MH, Ginsberg JS. Clinical risk factors and timing of recurrent venous thromboembolism during the initial 3 months of anticoagulant therapy. *Arch Intern Med.* 2000;160(22):3431-3436.

29. Pesavento R, de Conti G, Minotto I, et al. The value of 64-detector row computed tomography for the exclusion of pulmonary embolism. *Thromb Haemost.* 2011;105(5):901-907.

30. Raslan IA, Chong J, Gallix B, Lee TC, McDonald EG. Rates of Overtreatment and Treatment-Related Adverse Effects Among Patients With Subsegmental Pulmonary Embolism. *JAMA Intern Med.* 2018;178(9):1272-1274.

31. Yoo HH, Queluz TH, El Dib R. Anticoagulant treatment for subsegmental pulmonary embolism. *Cochrane Database Syst Rev.* 2016(1):CD010222.

32. Chan AW, Tetzlaff JM, Altman DG, et al. SPIRIT 2013 statement: defining standard protocol items for clinical trials. *Ann Intern Med.* 2013;158(3):200-207.

33. Peiman S, Abbasi M, Allameh SF, Asadi Gharabaghi M, Abtahi H, Safavi E. Subsegmental pulmonary embolism: A narrative review. *Thromb Res.* 2016;138:55-60.

34. Schoellnast H, Deutschmann HA, Fritz GA, Stessel U, Schaffler GJ, Tillich M. MDCT angiography of the pulmonary arteries: influence of iodine flow concentration on vessel attenuation and visualization. *AJR Am J Roentgenol.* 2005;184(6):1935-1939.

35. Klok FA, Huisman MV. Management of incidental pulmonary embolism. *Eur Respir J.* 2017;49(6).

36. van der Hulle T, den Exter PL, Planquette B, et al. Risk of recurrent venous thromboembolism and major hemorrhage in cancer-associated incidental pulmonary embolism among treated and untreated patients: a pooled analysis of 926 patients. *J Thromb Haemost.* 2016;14(1):105-113.

37. Kearon C, Ageno W, Cannegieter SC, et al. Categorization of patients as having provoked or unprovoked venous thromboembolism: guidance from the SSC of ISTH. *J Thromb Haemost.* 2016;14(7):1480-1483.

For peer review only - http://bmjopen.bmj.com/site/about/guidelines.xhtml

38. Prandoni P, Noventa F, Ghirarduzzi A, et al. The risk of recurrent venous thromboembolism after discontinuing anticoagulation in patients with acute proximal deep vein thrombosis or pulmonary embolism. A prospective cohort study in 1,626 patients. *Haematologica.* 2007;92(2):199-205.

39. Iorio A, Kearon C, Filippucci E, et al. Risk of recurrence after a first episode of symptomatic venous thromboembolism provoked by a transient risk factor: a systematic review. *Arch Intern Med.* 2010;170(19):1710-1716.

40. van der Hulle T, Tan M, den Exter PL, et al. Recurrence risk after anticoagulant treatment of limited duration for late, second venous thromboembolism. *Haematologica.* 2015;100(2):188-193.

41. Moores LK. Are We Overtreating Isolated Subsegmental Pulmonary Embolism?: First Do No Harm. *JAMA Intern Med.* 2018;178(9):1274-1275.

42. Agnelli G, Buller HR, Cohen A, et al. Oral apixaban for the treatment of acute venous thromboembolism. *N Engl J Med.* 2013;369(9):799-808.

43. Giugliano RP, Ruff CT, Braunwald E, et al. Edoxaban versus warfarin in patients with atrial fibrillation. *N Engl J Med.* 2013;369(22):2093-2104.

44. Aujesky D, Roy PM, Verschuren F, et al. Outpatient versus inpatient treatment for patients with acute pulmonary embolism: an international, open-label, randomised, non-inferiority trial. *Lancet.* 2011;378(9785):41-48.

45. Kearon C. Natural history of venous thromboembolism. *Circulation.* 2003;107(23 Suppl 1):I22-30.

46. Jimenez D, Aujesky D, Diaz G, et al. Prognostic significance of deep vein thrombosis in patients presenting with acute symptomatic pulmonary embolism. *Am J Respir Crit Care Med.* 2010;181(9):983-991.

For peer review only

For peer review only - http://bmjopen.bmj.com/site/about/guidelines.xhtml

47. Righini M, Le Gal G, Aujesky D, et al. Complete venous ultrasound in outpatients with suspected pulmonary embolism. *J Thromb Haemost.* 2009;7(3):406-412.

48. Buller HR, Cohen AT, Davidson B, et al. Idraparinux versus standard therapy for venous thromboembolic disease. *N Engl J Med.* 2007;357(11):1094-1104.

49. Lim W, Le Gal G, Bates SM, et al. American Society of Hematology 2018 guidelines for management of venous thromboembolism: diagnosis of venous thromboembolism. *Blood Adv.* 2018;2(22):3226-3256.

50. Mean M, Righini M, Jaeger K, et al. The Swiss cohort of elderly patients with venous thromboembolism (SWITCO65+): rationale and methodology. *J Thromb Thrombolysis.* 2013;36(4):475-483.

51. Schulman S, Kearon C. Definition of major bleeding in clinical investigations of antihemostatic medicinal products in non-surgical patients. *J Thromb Haemost.* 2005;3(4):692-694.

52. Raskob GE, Spyropoulos AC, Zrubek J, et al. The MARINER trial of rivaroxaban after hospital discharge for medical patients at high risk of VTE. Design, rationale, and clinical implications. *Thromb Haemost.* 2016;115(6):1240-1248.

53. Tritschler T, Kraaijpoel N, Girard P, et al. Definition of pulmonary embolism-related death and classification of the cause of death in venous thromboembolism studies: communication from the SSC of the ISTH. *J Thromb Haemost.* in press.

54. Cohn DM, Nelis EA, Busweiler LA, Kaptein AA, Middeldorp S. Quality of life after pulmonary embolism: the development of the PEmb-QoL questionnaire. *J Thromb Haemost.* 2009;7(6):1044-1046.

55. Klok FA, Cohn DM, Middeldorp S, et al. Quality of life after pulmonary embolism: validation of the PEmb-QoL Questionnaire. *J Thromb Haemost.* 2010;8(3):523-532.

For peer review only - http://bmjopen.bmj.com/site/about/guidelines.xhtml

56. Klok FA, Barco S, Siegerink B. Measuring functional limitations after venous thromboembolism: A call to action. *Thromb Res.* 2019;178:59-62.

57. Boon G, Barco S, Bertoletti L, et al. Measuring functional limitations after venous thromboembolism: Optimization of the Post-VTE Functional Status (PVFS) Scale. *Thromb Res.* 2020;190:45-51.

58. Yealy DM, Auble TE, Stone RA, et al. Effect of increasing the intensity of implementing pneumonia guidelines: a randomized, controlled trial. *Ann Intern Med.* 2005;143(12):881-894.

59. Carrier M, Kimpton M, G LEG, et al. The management of a sub-segmental pulmonary embolism: a cross-sectional survey of Canadian thrombosis physicians. *J Thromb Haemost.* 2011;9(7):1412-1415.

60. Mauri L, D'Agostino RB, Sr. Challenges in the Design and Interpretation of Noninferiority Trials. *N Engl J Med.* 2017;377(14):1357-1367.

61. Levine M, Gent M, Hirsh J, et al. A comparison of low-molecular-weight heparin administered primarily at home with unfractionated heparin administered in the hospital for proximal deep-vein thrombosis. *N Engl J Med.* 1996;334(11):677-681.

62. Koopman MM, Prandoni P, Piovella F, et al. Treatment of venous thrombosis with intravenous unfractionated heparin administered in the hospital as compared with subcutaneous low-molecular-weight heparin administered at home. The Tasman Study Group. *N Engl J Med.* 1996;334(11):682-687.

63. Columbus I, Buller HR, Gent M, et al. Low-molecular-weight heparin in the treatment of patients with venous thromboembolism. *N Engl J Med.* 1997;337(10):657-662.

64. Buller HR, Davidson BL, Decousus H, et al. Fondaparinux or enoxaparin for the initial treatment of symptomatic deep venous thrombosis: a randomized trial. *Ann Intern Med.* 2004;140(11):867-873.

For peer review only

For peer review only - http://bmjopen.bmj.com/site/about/guidelines.xhtml

65. Buller HR, Davidson BL, Decousus H, et al. Subcutaneous fondaparinux versus intravenous unfractionated heparin in the initial treatment of pulmonary embolism. *N Engl J Med.* 2003;349(18):1695-1702.

66. Agresti A, Caffo B. Simple and effective confidence intervals for proportions and differences of proportions result from adding two successes and two failures. *The American Statistician.* 2000;54:280-288.

67. Piaggio G, Elbourne DR, Pocock SJ, Evans SJ, Altman DG, Group C. Reporting of noninferiority and equivalence randomized trials: extension of the CONSORT 2010 statement. *JAMA.* 2012;308(24):2594-2604.

68. Fine JP, Gray RJ. Proportional hazards model for the subdistribution of a competing risk. *J Am Stat Assoc.* 1999;94:496-509.

For peer review only - http://bmjopen.bmj.com/site/about/guidelines.xhtml

Figure Legend

Figure. Overview of the Study Design.

SAFE-SSPE is a randomized, placebo-controlled, parallel-group non-inferiority trial. Low-risk patients with isolated SSPE without concomitant DVT are randomly assigned in a 1:1 ratio to receive placebo ("clinical surveillance group") or anticoagulant treatment with rivaroxaban ("anticoagulation group"). The primary study outcomes are symptomatic recurrent VTE within 90 days of randomization. Secondary outcomes include clinically significant bleeding and all-cause mortality, and ancillary outcomes will be health-related quality of life, functional status, and medical resource utilization.

Abbreviations: DVT, deep vein thrombosis; PE, pulmonary embolism; SSPE, subsegmental pulmonary embolism; VTE, venous thromboembolism.

For peer review only

For peer review only - http://bmjopen.bmj.com/site/about/guidelines.xhtml

1 **Table 1. Study Population, Intervention, Control, and Outcomes**

**Population**	Consecutive adult low-risk patients with an objective diagnosis of **isolated subsegmental PE** who have no concomitant DVT.
**Intervention**	**Clinical surveillance plus a matching rivaroxaban placebo,** one tablet twice daily for the first 21 days, followed by 1 tablet once daily for the remaining 90-da study period. Clinical surveillance is done at 10, 30 and 90 days following randomization by phone or by in-person visits, depending on local practice. At each contact, trained study personnel complete an assessment of symptoms and review for suspected recurrent VTE and bleeding using a checklist of pre-defined questions. Patients are also instructed to contact study personnel or report to the ED immediately if any symptoms/signs compatible with recurrent VTE or significant bleeding occur.
**Control**	**Clinical surveillance plus anticoagulation with rivaroxaban**, dosed at 15 mg twice daily for the first 21 days, followed by 20 mg once daily for the remaining 90-day study period. The same surveillance schedule as in the clinical surveillance group is used. Patients also receive the same instructions to contact study personnel or report to the ED if any signs or symptoms of VTE or significant bleeding occur.
**Outcomes**	Primary outcome: **recurrent, clinically symptomatic, objectively confirmed VTE** within 90 days of randomization, defined as recurrent fatal or nonfatal PE or lower limb DVT (efficacy).<br>Secondary outcomes: **clinically significant bleeding** and **all-cause mortality** at 90 days of randomization (safety).<br>Ancillary outcomes: **health-related quality of life, functional status, and medical resource utilization** at 90 days of randomization.<br>In a post-hoc analysis, **radiological inter-observer agreement** for SSPE will be assessed.

2

3 Abbreviations: DVT, deep vein thrombosis; ED, emergency department; PE, pulmonary embolism; SSPE,

4 subsegmental pulmonary embolism; VTE, venous thromboembolism.

5

For peer review only

For peer review only - http://bmjopen.bmj.com/site/about/guidelines.xhtml

Table 2. Exclusion Criteria and their Rationale

**Exclusion Criterion**	**Rationale**
1) Presence of leg DVT or upper extremity DVT (subclavian vein or above)	absolute indication for therapeutic anticoagulation
2) Active cancer	high risk of recurrent VTE if left untreated[36]
3) History of ≥1 prior episode of unprovoked VTE (±thrombophilia) [37]	high risk of recurrent VTE after stopping anticoagulation[38-40]
4) Clinical instability (systolic blood pressure <100 mm Hg or arterial oxygen saturation <92% at ambient air) at the time of presentation	risk of clinical deterioration[4,14,41]
5) Active bleeding or at high risk of bleeding (e.g., signs of active bleeding, ischemic stroke during preceding <10 days,[42] major gastrointestinal bleeding during preceding <3 months, intracranial or intraocular bleeding <6 months,[42] major trauma or surgery during preceding <1 month,[42,43] platelets <75,000 per mm^3,[44] or double anti-platelet therapy at the time of enrolment)	contraindication to rivaroxaban
6) Severe renal failure (creatinine clearance <30 ml/min.)	contraindication to rivaroxaban
7) Severe liver insufficiency (Child-Pugh B and C)	contraindication to rivaroxaban
8) Concomitant use of strong CYP3A4 inhibitors (i.e., HIV protease inhibitors [saquinavir, indinavir, ritonavir, nelfinavir, amprenavir, lopinavir, atazanavir, fosamprenavir, tipranavir, darunavir], systemic azole antifungals [i.e., ketoconazole, itraconazole, voriconazole, or posaconazole]) or strong CYP3A4 inducers (i.e., rifampicin, rifabutin, rifapentin, phenytoin, phenobarbital, primidone, carbamazepine, or St. John's Wort)	contraindication to rivaroxaban
9) Known hypersensitivity to rivaroxaban	contraindication to rivaroxaban
10) Need for therapeutic anticoagulation for another reason (atrial fibrillation/flutter, mechanical heart valves, previous VTE, known antiphospholipid antibody syndrome with unprovoked VTE)	randomization to placebo unethical
11) Therapeutic anticoagulation for >72 hours for any reason at the time of screening	could confound study outcomes
12) Hospitalized for >72 hours prior to the diagnosis of isolated SSPE (hospital-acquired VTE)	could confound study outcomes due to influence of co-treatments
13) Known pregnancy or breast-feeding	contraindication to rivaroxaban
14) Lack of safe contraception in women of childbearing potential	rivaroxaban is contraindicated in pregnancy
15) Refusal or inability to provide informed consent	unethical
16) Prior enrolment in this trial	confounds study outcomes

Abbreviations: DVT, deep vein thrombosis; VTE, venous thromboembolism

For peer review only

For peer review only - http://bmjopen.bmj.com/site/about/guidelines.xhtml

Table 3. Study Schedule

Study Period	Enrolment & Allocation	Baseline	Post-allocation		Close-out
**Visit/Follow-up phone call**	**1**		**2☽**††**	**3☽****	**4☽****
**Time (day with allowed visit window)**	**d0**	**d0**	**d10 (7-12)**	**d30 (28-35)**	**d90 (88-95)**
Eligibility screen (in-/exclusion criteria)	x				
Patient information and informed consent	x				
Bilateral whole-leg compression ultrasonography *	x				
Randomization	x				
Demographic characteristics		x			
Risk factors for venous thromboembolism		x			
Symptoms of venous thromboembolism		x			
Comorbid conditions		x			
Physical examination findings		x			
Laboratory test results		x			
Imaging findings		x			
Concomitant treatments		x			
Health-related quality of life (PEmb-QoL) †		x		x	x
Functional status ‡					x
Treatment setting		x			
Distribution and instruction of study drug		x			
Daily intake of study medication: placebo or rivaroxaban		x (immediate start on day 0)			
Instructions & distribution of patient diary §		x			
Recurrent venous thromboembolism			x	x	x
Clinically significant bleeding			x	x	x
All-cause mortality			x	x	x
Medical resource utilization			x	x	x
Time to symptom resolution			x	x	x
New concomitant treatments			x	x	x
Interruption of the study drug			x	x	x
Adherence ||					x
Serious adverse events reporting #		x (immediate start after inclusion on day 0)			

* Participants with concomitant deep vein thrombosis are excluded from further study participation (screening failures).

† At the baseline visit, participants are asked to fill in the PEmb-QoL questionnaire. For follow-up assessments, participants receive the PEmb-QoL including a pre-stamped envelope by mail and are asked

For peer review only

For peer review only - http://bmjopen.bmj.com/site/about/guidelines.xhtml

to complete and return the questionnaire. If the follow-up interview is done in-person, the PEmb-QoL can be administered during the office visit.

‡ Functional status is assessed using the post-VTE functional status scale

§ The first part of the patient diary consists of an information on the study outline, surveillance interviews, and symptoms and signs suggestive for recurrent venous thromboembolism and bleeding, the investigator's contact information/emergency telephone number if these symptoms/signs occur, how to take the study medications, and instructions to return the drug bottles at the end of the study. The second part consists of a patient diary where patients are asked to record dates and type of outcomes and measures of health resource utilization (e.g., physician visits), and the time to symptom resolution and return to work/usual activities.

|| Adherence is assessed by counting the pill count of the returned medication bottles.

A serious adverse event is defined as any untoward medical occurrence that results in in death, is life-threatening, requires in-patient hospitalization or prolongation of existing hospitalization, results in persistent or significant disability/incapacity, or is a congenital anomaly/birth defect. In addition, important medical events that may jeopardize the patient or may require an intervention to prevent one of these outcomes is also considered serious.

** phone or in-person follow-up, depending on local practice

†† see Supplementary File for the case report form of the 10-day follow-up interview as an illustration of the content of the follow-up phone calls

For peer review only - http://bmjopen.bmj.com/site/about/guidelines.xhtml

For peer review only - http://bmjopen.bmj.com/site/about/guidelines.xhtml

Figure. Overview of the Study Design

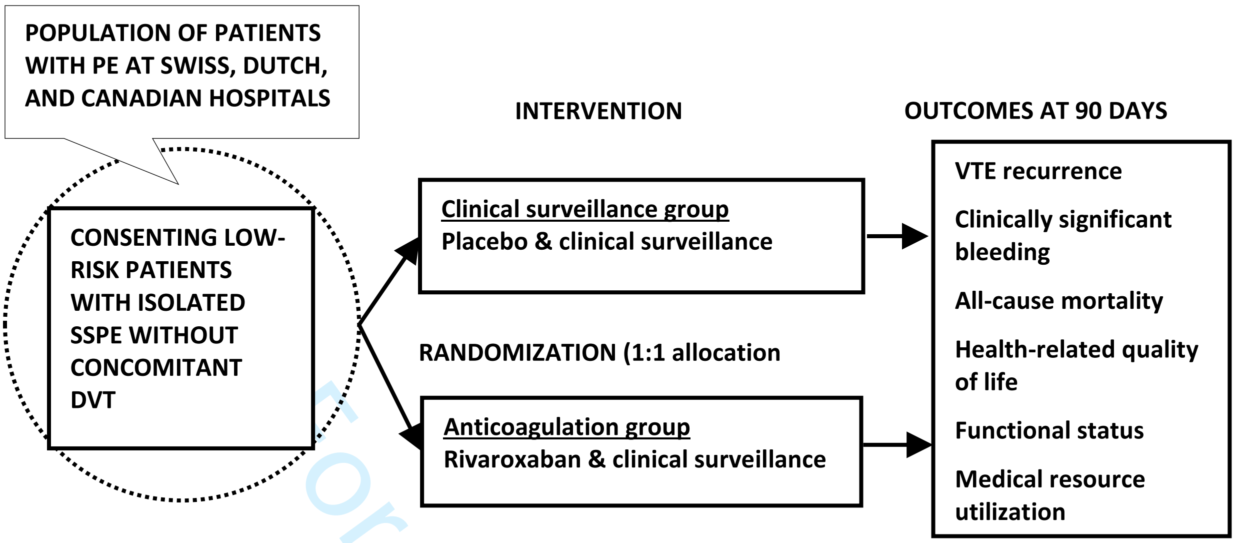

For peer review only

<u>Supplementary File: Case Report Form (CRF) for follow-up interview 10 days after randomization.</u>

For peer review only

Patient ID

Follow up interview

(04.05.2020 - 13:25:25 (CEST))

**Follow-up interview number**	○ 1 ○ 2 ○ 3
**Date of follow-up interview phone call**	[ ] dd.mm.yyyy
**Follow up interview completed**	○ yes ○ no
**Please indicate the reason why follow-up call wasn't completed**	○ lost to follow-up ○ withdrawal from study ○ patient died
**Please specify the reason for withdrawal**	
**Contact(s) made to obtain follow-up information**	
**Patient**	☐
**Designated contact person (family or friend)**	☐
**Primary care physician**	☐
**Other**	☐
**Please specify "other"**	
**Agreement for consecutive follow up**	○ patient agrees to be contacted for final assessment at 90 days<br>○ patient agrees for passive follow-up (i.e. information collected from hospital medical records and/or primary care physician)
**Planned date of next follow-up call**	[ ] dd.mm.yyyy

For peer review only - http://bmjopen.bmj.com/site/about/guidelines.xhtml

Patient ID

Medical outcomes

(04.05.2020 - 13:25:25 (CEST))

VTE RECURRENCE

VTE recurrence ○ Yes ○ No

Interruption of study medication and unblinding is indicated if VTE is objectively confirmed

Date of diagnosis [ ] dd.mm.yyyy

Confirmatory diagnostic exam:

- CTPA ☐
- ventilation-perfusion scintigraphy ☐
- pulmonary angiography ☐
- venous ultrasound ☐
- IV phlebography ☐
- CT or MRI for thrombosis of the iliac vein or vena cava inferior ☐
- echocardiography ☐

Type of recurrent VTE ○ PE ○ DVT

Localization of PE ○ subsegmental PE ○ more proximal PE

Localization of DVT ○ proximal DVT (V.cava, iliac vein, common or superfician femoral vein, popliteal vein) ○ distal DVT (posterior or anterior tibial vein, peroneal vein, gastrocnemial vein, soleal vein)

Stop of study medication following recurrent VTE ○ Yes ○ No

Enter the date of stopping the study medication [ ] dd.mm.yyyy

CLINICALLY SIGNIFICANT BLEEDING

Clinically significant bleeding ○ Yes ○ No

Clinically significant bleeding 1

Date of bleeding episode [ ] dd.mm.yyyy

Type of physician contact(s)

- phone call to physician ☐
- office visit ☐
- ED visit ☐
- hospitalization ☐
- none ☐

Treatment with antiplatelet agent or NSAID at the time of bleeding ○ Yes ○ No

- Aspirin ☐
- clopidogrel ☐

For peer review only - http://bmjopen.bmj.com/site/about/guidelines.xhtml

Patient ID

prasugrel	☐
ticagrelor	☐
NSAIDs	☐
**Packed red cell transfusions**	○ Yes ○ No
Indicate the number of units	☐
**Decrease in hemoglobin of at least 20 g/L within 7 days after the bleeding event**	○ Yes ○ No ○ Unknown
**Site of bleeding**	
epistaxis	☐
cutaneous	☐
subcutaneous	☐
intramuscular with compartment syndrome	☐
intramuscular without compartment syndrome	☐
gastrointestinal [melena, hematochezia, hematemesis]	☐
intracranial	☐
retroperitoneal	☐
pulmonary	☐
intraarticular	☐
intraspinal	☐
intraocular	☐
pericardial	☐
hematuria	☐
vaginal	☐
other	☐
other, please specify	
**Diagnostic test(s) performed to localize bleeding source**	
MRI	☐
CT	☐
ultrasound	☐
upper endoscopy	☐
lower endoscopy	☐
capsule endoscopy	☐
bronchoscopy	☐

Medical Outcomes

Patient ID

cystoscopy	☐
arteriography	☐
erythrocyte scintigraphy	☐
diagnostic surgery	☐
other	☐
other, please specify	
**Treatment(s) of bleeding:**	
fresh frozen plasma	☐
recombinant factor VIIa concentrate	☐
prothrombin complex [factor II, VII, IX, X]	☐
factor Xa inhibitor-specific antidote [andexanet alpha]	☐
tranexamic acid	☐
invasive or surgical hemostasis procedures	☐
other	☐
other, please specify	
Interruption of study medication following bleeding	○ Yes ○ No
duration of interruption	○ definite ○ temporary
date of interruption	dd.mm.yyyy
date of restart	dd.mm.yyyy
**Clinical impact of overt bleeding:**	
temporary or permanent cessation of the study drug	○ Yes ○ No
overt bleeding associated with relevant pain	○ Yes ○ No
impairment of activities of daily life	○ Yes ○ No

More

Patient ID

Medical resource utilization

(04.05.2020 - 13:25:25 (CEST))

Initial hospitalization ongoing ○ Yes ○ No

Date of discharge from initial hospitalization [ ] dd.mm.yyyy hh:mm

Subsequent hospitalizations ○ Yes ○ No

Subsequent hospitalizations 1

Admission date [ ] dd.mm.yyyy

Discharge date [ ] dd.mm.yyyy

Name of hospital [ ]

Symptoms or signs leading to hospitalization

- **dyspnea** ☐
- **cough** ☐
- **chest pain** ☐
- **bloody cough [hemoptysis]** ☐
- **syncope** ☐
- **pleural effusion** ☐
- **leg pain** ☐
- **leg swelling** ☐
- **bleeding** ☐
- **other** ☐
  - **please specify** [ ]

Primary discharge diagnosis [ ]

Please specify [ ]

Discharge location [ ]

Other, please specify [ ]

Notes [ ]

More

Subsequent emergency department visits ○ Yes ○ No

Subsequent emergency department visits 1

Presentation date [ ] dd.mm.yyyy

Specify hospital name [ ]

Patient ID

Symptoms or signs leading to ED visit

- **dyspnea** ☐
- **cough** ☐
- **chest pain** ☐
- **bloody cough [hemoptysis]** ☐
- **syncope** ☐
- **pleural effusion** ☐
- **leg pain** ☐
- **leg swelling** ☐
- **bleeding** ☐
- **other** ☐
  - **please specify**

Primary diagnosis at the emergency department

Please specify

Notes

More

Subsequent physician outpatient visits ○ Yes ○ No

Subsequent physician outpatient visits 1

Visit date dd.mm.yyyy

Reason for visit

- **dyspnea** ☐
- **cough** ☐
- **chest pain** ☐
- **bloody cough [hemoptysis]** ☐
- **pleural effusion** ☐
- **leg pain** ☐
- **leg swelling** ☐
- **bleeding** ☐
- **other** ☐

Please specify

For peer review only - http://bmjopen.bmj.com/site/about/guidelines.xhtml

Patient ID

Physician diagnosis

Notes

More

Medical Resource Utilization

For peer review only

Patient ID

Time to symptom resolution

(04.05.2020 - 13:25:25 (CEST))

**Resolution of dyspnea**	○ Yes ○ No ○ Symptom has not been present
**Date of symptom resolution**	[ ] dd.mm.yyyy
**Resolution of cough**	○ Yes ○ No ○ Symptom has not been present
**Date of symptom resolution**	[ ] dd.mm.yyyy
**Resolution of chest pain**	○ Yes ○ No ○ Symptom has not been present
**Date of symptom resolution**	[ ] dd.mm.yyyy
**Resolution of bloody cough**	○ Yes ○ No ○ Symptom has not been present
**Date of symptom resolution**	[ ] dd.mm.yyyy
**Returned to work or usual activities**	○ Yes ○ No
**date of returning to work or usual activities**	[ ] dd.mm.yyyy

For peer review only - http://bmjopen.bmj.com/site/about/guidelines.xhtml

Patient ID

Discontinuation or modification of study medication

(04.05.2020 - 13:25:25 (CEST))

Was another anticoagulant agent started during study period? ○ Yes ○ No

Type of anticoagulant [ ]

Please specify [ ]

Duration of anticoagulant treatment ○ extended ○ temporary

Date of starting the anticoagulant [ ] dd.mm.yyyy

Date of stopping anticoagulant [ ] dd.mm.yyyy

Dose of anticoagulant ○ therapeutic ○ prophylactic

Reason for starting another anticoagulant

- **Atrial fibrillation / atrial flutter with a CHADS2 score of ≥1 or CHA2DS2-VASc ≥2** ☐
- **mechanical heart valve** ☐
- **upper or lower extremity DVT** ☐
- **pulmonary embolism** ☐
- **intraabdominal thrombosis (e.g. portal vein thrombosis)** ☐
- **intracranial vein thrombosis** ☐
- **VTE prophylaxis** ☐
- **bridging for intervention/procedure** ☐
- **other** ☐
- **please specify** [ ]

Discontinuation of study medication during follow-up ○ Yes ○ No

Date of discontinuation [ ] dd.mm.yyyy

Reason for discontinuation:

need for prophylactic anticoagulation ☐

new indication for permanent therapeutic anticoagulation ☐

specify indication:

- **new diagnosis of atrial fibrillation/flutter with a CHADS2 score of ≥1 or CHA2DS2-VASc ≥2** ☐
- **mechanical heart valve** ☐
- **upper or lower extremity DVT** ☐

For peer review only - http://bmjopen.bmj.com/site/about/guidelines.xhtml

Patient ID

pulmonary embolism ☐

intraabdominal thrombosis (e.g. portal vein thrombosis) ☐

intracranial vein thrombosis ☐

other ☐

please specify

bleeding ☐

elective invasive procedure or surgical intervention requiring interruption of anticoagulation ☐

please specify procedure/ intervention

emergency invasive procedure or surgical intervention requiring interruption of anticoagulation ☐

please specify procedure/ intervention

was an emergency anticoagulation reversal done prior to the emergency procedure? ○ Yes ○ No

indicate agent/method used for anticoagulation reversal:

fresh frozen plasma ☐

recombinant factor VIIa concentrate ☐

prothrombin complex [factor II, VII, IX, X] ☐

factor Xa inhibitor-specific antidote [andexanet alpha] ☐

tranexamic acid ☐

invasive or surgical hemostasis procedures) ☐

For peer review only - http://bmjopen.bmj.com/site/about/guidelines.xhtml

Patient ID

new diagnosis of pregnancy	☐
new use of medications interacting with rivaroxaban	☐
strong CYP3A4 inhibitors or inducers	☐
saquinavir	☐
indinavir	☐
ritonavir	☐
nelfinavir	☐
atazanavir	☐
fosamprenavir	☐
tipranavir	☐
darunavir	☐
ketoconazole	☐
itraconazole	☐
voriconazole	☐
posaconazole	☐
rifampicin	☐
rifabutin	☐
rifapentin	☐
phenytoin	☐
phenobarbital	☐
primidone	☐
carbamazepine	☐
St. John's Wort	☐
specify indication	
HIV	☐
fungal infection	☐
epilepsy	☐
depression	☐
other	☐
please specify	

new dual antiplatelet therapy (aspirin plus P2Y12-inhibitor)	☐
Please specify	○ Aspirin plus clopidogrel<br>○ aspirin plus ticagrelor<br>○ Aspirin plus prasugrel<br>○ Other

For peer review only - http://bmjopen.bmj.com/site/about/guidelines.xhtml

Patient ID

Please specify indication

new GP IIb/IIIa inhibitors ☐

specify medication
- ○ abciximab
- ○ eptifibatide
- ○ tirofiban

specify indication

patient withdraws from study participation ☐

Was the discontinuation temporary? ○ Yes ○ No

Date of resuming study medication dd.mm.yyyy

iew only

For peer review only - http://bmjopen.bmj.com/site/about/guidelines.xhtml

STANDARD PROTOCOL ITEMS: RECOMMENDATIONS FOR INTERVENTIONAL TRIALS

SPIRIT 2013 Checklist: Recommended items to address in a clinical trial protocol and related documents*

Section/item	Item No	Description	Page No
**Administrative information**			
Title	1	Descriptive title identifying the study design, population, interventions, and, if applicable, trial acronym	1
Trial registration	2a	Trial identifier and registry name. If not yet registered, name of intended registry	4
	2b	All items from the World Health Organization Trial Registration Data Set	4
Protocol version	3	Date and version identifier	4
Funding	4	Sources and types of financial, material, and other support	3
Roles and responsibilities	5a	Names, affiliations, and roles of protocol contributors	1, 22
	5b	Name and contact information for the trial sponsor	23
	5c	Role of study sponsor and funders, if any, in study design; collection, management, analysis, and interpretation of data; writing of the report; and the decision to submit the report for publication, including whether they will have ultimate authority over any of these activities	22
	5d	Composition, roles, and responsibilities of the coordinating centre, steering committee, endpoint adjudication committee, data management team, and other individuals or groups overseeing the trial, if applicable (see Item 21a for data monitoring committee)	22-25
**Introduction**			
Background and rationale	6a	Description of research question and justification for undertaking the trial, including summary of relevant studies (published and unpublished) examining benefits and harms for each intervention	6
	6b	Explanation for choice of comparators	6
Objectives	7	Specific objectives or hypotheses	8

For peer review only

Trial design	8	Description of trial design including type of trial (eg, parallel group, crossover, factorial, single group), allocation ratio, and framework (eg, superiority, equivalence, noninferiority, exploratory)	8
**Methods: Participants, interventions, and outcomes**			
Study setting	9	Description of study settings (eg, community clinic, academic hospital) and list of countries where data will be collected. Reference to where list of study sites can be obtained	8
Eligibility criteria	10	Inclusion and exclusion criteria for participants. If applicable, eligibility criteria for study centres and individuals who will perform the interventions (eg, surgeons, psychotherapists)	9
Interventions	11a	Interventions for each group with sufficient detail to allow replication, including how and when they will be administered	10
	11b	Criteria for discontinuing or modifying allocated interventions for a given trial participant (eg, drug dose change in response to harms, participant request, or improving/worsening disease)	12
	11c	Strategies to improve adherence to intervention protocols, and any procedures for monitoring adherence (eg, drug tablet return, laboratory tests)	11
	11d	Relevant concomitant care and interventions that are permitted or prohibited during the trial	11
Outcomes	12	Primary, secondary, and other outcomes, including the specific measurement variable (eg, systolic blood pressure), analysis metric (eg, change from baseline, final value, time to event), method of aggregation (eg, median, proportion), and time point for each outcome. Explanation of the clinical relevance of chosen efficacy and harm outcomes is strongly recommended	12-14
Participant timeline	13	Time schedule of enrolment, interventions (including any run-ins and washouts), assessments, and visits for participants. A schematic diagram is highly recommended (see Figure)	37
Sample size	14	Estimated number of participants needed to achieve study objectives and how it was determined, including clinical and statistical assumptions supporting any sample size calculations	15-16

For peer review only - http://bmjopen.bmj.com/site/about/guidelines.xhtml

Recruitment	15	Strategies for achieving adequate participant enrolment to reach target sample size	10
**Methods: Assignment of interventions (for controlled trials)**			
Allocation:			
Sequence generation	16a	Method of generating the allocation sequence (eg, computer-generated random numbers), and list of any factors for stratification. To reduce predictability of a random sequence, details of any planned restriction (eg, blocking) should be provided in a separate document that is unavailable to those who enrol participants or assign interventions	8
Allocation concealment mechanism	16b	Mechanism of implementing the allocation sequence (eg, central telephone; sequentially numbered, opaque, sealed envelopes), describing any steps to conceal the sequence until interventions are assigned	8
Implementation	16c	Who will generate the allocation sequence, who will enrol participants, and who will assign participants to interventions	8
Blinding (masking)	17a	Who will be blinded after assignment to interventions (eg, trial participants, care providers, outcome assessors, data analysts), and how	8-9
	17b	If blinded, circumstances under which unblinding is permissible, and procedure for revealing a participant's allocated intervention during the trial	12
**Methods: Data collection, management, and analysis**			
Data collection methods	18a	Plans for assessment and collection of outcome, baseline, and other trial data, including any related processes to promote data quality (eg, duplicate measurements, training of assessors) and a description of study instruments (eg, questionnaires, laboratory tests) along with their reliability and validity, if known. Reference to where data collection forms can be found, if not in the protocol	11
	18b	Plans to promote participant retention and complete follow-up, including list of any outcome data to be collected for participants who discontinue or deviate from intervention protocols	15

For peer review only - http://bmjopen.bmj.com/site/about/guidelines.xhtml

Data management	19	Plans for data entry, coding, security, and storage, including any related processes to promote data quality (eg, double data entry; range checks for data values). Reference to where details of data management procedures can be found, if not in the protocol	11
Statistical methods	20a	Statistical methods for analysing primary and secondary outcomes. Reference to where other details of the statistical analysis plan can be found, if not in the protocol	16
	20b	Methods for any additional analyses (eg, subgroup and adjusted analyses)	16-18
	20c	Definition of analysis population relating to protocol non-adherence (eg, as randomised analysis), and any statistical methods to handle missing data (eg, multiple imputation)	16- 18
**Methods: Monitoring**			
Data monitoring	21a	Composition of data monitoring committee (DMC); summary of its role and reporting structure; statement of whether it is independent from the sponsor and competing interests; and reference to where further details about its charter can be found, if not in the protocol. Alternatively, an explanation of why a DMC is not needed	18
	21b	Description of any interim analyses and stopping guidelines, including who will have access to these interim results and make the final decision to terminate the trial	18, 18
Harms	22	Plans for collecting, assessing, reporting, and managing solicited and spontaneously reported adverse events and other unintended effects of trial interventions or trial conduct	(18), 37
Auditing	23	Frequency and procedures for auditing trial conduct, if any, and whether the process will be independent from investigators and the sponsor	11
**Ethics and dissemination**			
Research ethics approval	24	Plans for seeking research ethics committee/institutional review board (REC/IRB) approval	20

For peer review only - http://bmjopen.bmj.com/site/about/guidelines.xhtml

Protocol amendments	25	Plans for communicating important protocol modifications (eg, changes to eligibility criteria, outcomes, analyses) to relevant parties (eg, investigators, REC/IRBs, trial participants, trial registries, journals, regulators)	19
Consent or assent	26a	Who will obtain informed consent or assent from potential trial participants or authorised surrogates, and how (see Item 32)	10, 20
	26b	Additional consent provisions for collection and use of participant data and biological specimens in ancillary studies, if applicable	n.a.
Confidentiality	27	How personal information about potential and enrolled participants will be collected, shared, and maintained in order to protect confidentiality before, during, and after the trial	20
Declaration of interests	28	Financial and other competing interests for principal investigators for the overall trial and each study site	22
Access to data	29	Statement of who will have access to the final trial dataset, and disclosure of contractual agreements that limit such access for investigators	19
Ancillary and post-trial care	30	Provisions, if any, for ancillary and post-trial care, and for compensation to those who suffer harm from trial participation	n.a.
Dissemination policy	31a	Plans for investigators and sponsor to communicate trial results to participants, healthcare professionals, the public, and other relevant groups (eg, via publication, reporting in results databases, or other data sharing arrangements), including any publication restrictions	20
	31b	Authorship eligibility guidelines and any intended use of professional writers	(23)
	31c	Plans, if any, for granting public access to the full protocol, participant-level dataset, and statistical code	19
**Appendices**			
Informed consent materials	32	Model consent form and other related documentation given to participants and authorised surrogates	
Biological specimens	33	Plans for collection, laboratory evaluation, and storage of biological specimens for genetic or molecular analysis in the current trial and for future use in ancillary studies, if applicable	n/a

For peer review only - http://bmjopen.bmj.com/site/about/guidelines.xhtml

*It is strongly recommended that this checklist be read in conjunction with the SPIRIT 2013 Explanation & Elaboration for important clarification on the items. Amendments to the protocol should be tracked and dated. The SPIRIT checklist is copyrighted by the SPIRIT Group under the Creative Commons "Attribution-NonCommercial-NoDerivs 3.0 Unported" license.

For peer review only

BMJ Open

Clinical Surveillance vs. Anticoagulation For low-risk patiEnts with isolated SubSegmental Pulmonary Embolism: Protocol for a multicenter randomized placebo-controlled non-inferiority trial (SAFE-SSPE)

Journal:	*BMJ Open*
Manuscript ID	bmjopen-2020-040151.R2
Article Type:	Protocol
Date Submitted by the Author:	08-Sep-2020
Complete List of Authors:	Baumgartner, Christine; Inselspital University Hospital Bern, General Internal Medicine<br>Klok, Frederikus; Leiden University Medical Center, Department of Thrombosis and Hemostasis<br>Carrier, Marc; Ottawa Hospital Research Institute<br>Limacher, Andreas; Universitat Bern Institut fur Sozial- und Praventivmedizin<br>Moor, Jeanne; Inselspital University Hospital Bern, Department of General Internal Medicine<br>Righini, Marc; Geneva University Hospitals, Division of Angiology and Haemostasis<br>Beer, Jürg-Hans; Kantonsspital Baden AG, Department of Internal Medicine<br>Peluso, Martina; Inselspital University Hospital Bern, Department of General Internal Medicine<br>Rakovic, Damiana; Inselspital University Hospital Bern, Department of General Internal Medicine<br>Huisman, Menno; Leiden University Medical Center, Department of Thrombosis and Hemostasis<br>AUJESKY , Drahomir; Inselspital University Hospital Bern, Department of General Internal Medicine
**Primary Subject Heading**:	Cardiovascular medicine
Secondary Subject Heading:	Emergency medicine, Evidence based practice, Research methods
Keywords:	Anticoagulation < HAEMATOLOGY, Thromboembolism < CARDIOLOGY, Clinical trials < THERAPEUTICS

SCHOLARONE™
Manuscripts

I, the Submitting Author has the right to grant and does grant on behalf of all authors of the Work (as defined in the below author licence), an exclusive licence and/or a non-exclusive licence for contributions from authors who are: i) UK Crown employees; ii) where BMJ has agreed a CC-BY licence shall apply, and/or iii) in accordance with the terms applicable for US Federal Government officers or employees acting as part of their official duties; on a worldwide, perpetual, irrevocable, royalty-free basis to BMJ Publishing Group Ltd ("BMJ") its licensees and where the relevant Journal is co-owned by BMJ to the co-owners of the Journal, to publish the Work in this journal and any other BMJ products and to exploit all rights, as set out in our licence.

The Submitting Author accepts and understands that any supply made under these terms is made by BMJ to the Submitting Author unless you are acting as an employee on behalf of your employer or a postgraduate student of an affiliated institution which is paying any applicable article publishing charge ("APC") for Open Access articles. Where the Submitting Author wishes to make the Work available on an Open Access basis (and intends to pay the relevant APC), the terms of reuse of such Open Access shall be governed by a Creative Commons licence – details of these licences and which Creative Commons licence will apply to this Work are set out in our licence referred to above.

Other than as permitted in any relevant BMJ Author's Self Archiving Policies, I confirm this Work has not been accepted for publication elsewhere, is not being considered for publication elsewhere and does not duplicate material already published. I confirm all authors consent to publication of this Work and authorise the granting of this licence.

For peer review only

Clinical Surveillance vs. Anticoagulation For low-risk patiEnts with isolated SubSegmental Pulmonary Embolism: Protocol for a multicenter randomized placebo-controlled non-inferiority trial (SAFE-SSPE)

Christine Baumgartner, MD, MAS [1]; Frederikus A. Klok, MD, PhD, FESC [2]; Marc Carrier, MD, MSc [3]; Andreas Limacher, PhD [4]; Jeanne Moor, MD [1]; Marc Righini, MD [5]; Jürg-Hans Beer, MD [6]; Martina Peluso [1]; Damiana Rakovic [1]; Menno V. Huisman, MD, PhD [2]; Drahomir Aujesky, MD, MSc [1]

Author Affiliations:

[1] Department of General Internal Medicine, Inselspital, Bern University Hospital, University of Bern, Switzerland

[2] Department of Thrombosis and Hemostasis, Leiden University Medical Center, Leiden, the Netherlands

[3] Department of Medicine, The Ottawa Hospital Research Institute at the University of Ottawa, Ottawa, Canada

[4] CTU Bern, University of Bern, Bern, Switzerland

[5] Division of Angiology and Haemostasis, Geneva University Hospital, University of Geneva, Switzerland

[6] Department of Internal Medicine, Cantonal Hospital of Baden, Baden, Switzerland

Corresponding Author:

Christine Baumgartner, MD, MAS, Department of General Internal Medicine, Inselspital, Bern University Hospital, University of Bern, Freiburgstrasse, 3010 Bern, Switzerland. Phone: +41 (0)31 632 57 69; e-mail: Christine.Baumgartner@insel.ch

For peer review only

For peer review only - http://bmjopen.bmj.com/site/about/guidelines.xhtml

Abstract word count: 281, **Text word count:** 4364**, Tables:** 3**, Figures:** 1**, Number of References:** 70

Keywords: subsegmental pulmonary embolism, anticoagulation, randomized clinical trial

For peer review only

For peer review only - http://bmjopen.bmj.com/site/about/guidelines.xhtml

Abstract

Introduction: The clinical significance of subsegmental pulmonary embolism (SSPE) is currently unclear. Although growing evidence from observational studies suggests that withholding anticoagulant treatment may be a safe option in selected patients with isolated SSPE, most patients with this condition receive anticoagulant treatment, which is associated with a 90-day risk of recurrent VTE of 0.8% and major bleeding of up to 5%. Given the ongoing controversy concerning the risk-benefit ratio of anticoagulation for isolated SSPE and the lack of evidence from randomized-controlled studies, the aim of this clinical trial is to evaluate the efficacy and safety of clinical surveillance without anticoagulation in low-risk patients with isolated SSPE.

Methods and Analysis: SAFE-SSPE (Surveillance vs. Anticoagulation For low-risk patiEnts with isolated SubSegmental Pulmonary Embolism: a multicenter randomized placebo-controlled non-inferiority trial) is an international, multicenter, placebo-controlled, double-blind, parallel-group non-inferiority trial conducted in Switzerland, the Netherlands, and Canada. Low-risk patients with isolated SSPE are randomized to receive clinical surveillance with either placebo (no anticoagulation) or anticoagulant treatment with rivaroxaban. All patients undergo bilateral whole-leg compression ultrasonography to exclude concomitant deep vein thrombosis before enrolment. Patients are followed for 90 days. The primary outcome is symptomatic recurrent VTE (efficacy). The secondary outcomes include clinically significant bleeding and all-cause mortality (safety). The ancillary outcomes are health-related quality of life, functional status, and medical resource utilization.

Ethics and Dissemination: The local Ethics Committees in Switzerland have approved this protocol. Submission to the Ethical Committees in the Netherlands and Canada is underway. The results of this trial will be published in a peer-reviewed journal.

Main Funding: Investigator Initiated Clinical Trials funding scheme of the Swiss National Science Foundation (SNSF). The study medication is provided free of charge by Bayer AG.

For peer review only - http://bmjopen.bmj.com/site/about/guidelines.xhtml

Trial Registration Numbers:

ClinicalTrials.gov: NCT04263038

Protocol version 2.0, 23.01.2020

For peer review only

For peer review only - http://bmjopen.bmj.com/site/about/guidelines.xhtml

Article Summary: Strengths and Limitations of this Study

- This is the first randomized trial comparing the safety and efficacy of a management strategy without anticoagulation and anticoagulant treatment in low-risk patients with isolated subsegmental pulmonary embolism (SSPE).
- We chose patient-centered outcomes and economically relevant efficiency measures that are relevant to both patient and health care professionals.
- As patients with isolated SSPE and a high risk of adverse events (e.g., patients with cancer) are excluded from this trial due to safety reasons, generalizability of the results will be limited to low-risk patients with isolated SSPE.

For peer review only

Introduction

Depending on thromboembolic burden and patient factors, the clinical spectrum of pulmonary embolism (PE) ranges from asymptomatic cases to massive PE with hemodynamic collapse.[1,2] Anticoagulant treatment for at least 3 months effectively reduces the risk of recurrent venous thromboembolism (VTE).[3 4] The benefit of anticoagulation, however, comes at the cost of potentially disabling and life-threatening bleeding events, with a 90-day risk of major bleeding of up to 5%.[5-8]

The widespread introduction and technological advances of multi-detector computed tomography pulmonary angiography (CTPA) have led to an 80% increase in the diagnosis of acute PE between 1998 and 2006.[9] This increase is in part due to an increase in the detection of small, peripheral PE limited to the subsegmental pulmonary arteries, i.e. subsegmental PE (SSPE),[10,11] which currently comprise 10-15% of cases with PE.[12-15] However, the clinical significance of isolated SSPE is questionable. Epidemiologic evidence suggests overdiagnosis: despite the increase in PE diagnoses in recent years, PE-related mortality has remained stable or has even decraesed.[9,16] The positive predictive value of CTPA to diagnose SSPE is as low as 25% (compared to a composite reference standard with ventilation-perfusion lung scanning with or without lower limb venous ultrasonography, or pulmonary digital-subtraction angiography),[17] and inter-observer agreement between radiologists for SSPE diagnosis is only fair,[18] indicating that differentiation between true emboli and artifacts in the subsegmental pulmonary arteries is difficult. Small PE may even occur in healthy individuals without clinical consequences,[19-21] suggesting that SSPE may be the result from of the physiological filter function of the lung to protect the systemic circulation.

Whether patients with isolated SSPE benefit from anticoagulant treatment is currently uncertain.[4] There is growing evidence from observational studies that withholding anticoagulation may be safe in patients with isolated SSPE who are at low risk of recurrent or progressive VTE,[22-27] but most of such patients currently receive anticoagulant treatment,[28-31] potentially exposing them to an unnecessary risk of bleeding. Given the ongoing controversy and clinical equipoise about the risk-benefit ratio of anticoagulation for isolated SSPE, we aim to evaluate the efficacy and safety of

For peer review only - http://bmjopen.bmj.com/site/about/guidelines.xhtml

clinical surveillance without anticoagulation compared to standard anticoagulation treatment in low-risk patients with SSPE in a randomized clinical trial.

For peer review only

For peer review only - http://bmjopen.bmj.com/site/about/guidelines.xhtml

Methods and Analysis

This study protocol has been developed according to the Standard Protocol Items: Recommendation for Interventional Trials (SPIRIT) guidelines.[32]

Objectives and Hypotheses

The primary objective of this randomized trial is to compare the frequency of symptomatic, recurrent VTE in low-risk patients with isolated SSPE randomized to receive clinical surveillance plus placebo or clinical surveillance plus anticoagulant treatment with rivaroxaban. As a secondary objective, the frequency of clinically significant bleeding and all-cause mortality is compared in the two groups. Ancillary endpoints include health-related quality of life, functional status, and medical resource utilization. We hypothesize that clinical surveillance without anticoagulant treatment is non-inferior to anticoagulation in terms of recurrent VTE, while resulting in fewer clinically significant bleeding events and similar all-cause mortality. We also hypothesize that clinical surveillance, compared to anticoagulation, improves health-related quality of life and functional status and reduces medical resource utilization.

Study Design and Setting

SAFE-SSPE (clinical Surveillance vs. Anticoagulation For low-risk patiEnts with isolated SubSegmental Pulmonary Embolism) is an investigator-initiated, multicenter, randomized, placebo-controlled, double-blind, parallel-group non-inferiority trial. Patients with isolated SSPE at low risk for VTE progression or recurrence and without concomitant deep vein thrombosis (DVT) are randomly assigned in a 1:1 ratio to receive clinical surveillance plus placebo or clinical surveillance plus anticoagulation with rivaroxaban (**Figure**). Randomization is blocked and stratified by study site. To ensure concealment of allocation, the allocation sequence is generated by a data manager not involved in the study, using a computer-generated randomization schedule. All participants, care providers, investigators, study personnel, members of the outcomes adjudication committee, data management personnel, and analysts are blinded to group assignment. Eligible patients are recruited

For peer review only - http://bmjopen.bmj.com/site/about/guidelines.xhtml

in at least 27 university and medium- to high-volume non-university teaching hospitals in Switzerland, the Netherlands, and Canada.

Selection of Patients

Consecutive patients aged ≥18 years with objectively diagnosed symptomatic or asymptomatic isolated SSPE on CTPA based on the assessment of the local radiologist at the time of patient presentation are eligible for study participation after provision of informed consent (**Table 1**). Isolated SSPE is defined as multi-detector CTPA demonstrating an intraluminal filling defect in ≥1 subsegmental pulmonary artery (4th order or higher) without filling defects visualized at more proximal pulmonary artery levels.[24,33,34] Isolated subsegmental defects are classified as either single (1 subsegmental vessel involved) or multiple (≥2 subsegmental vessels involved). Patients with both symptomatic and incidentally detected, asymptomatic isolated SSPE are potentially eligible because symptoms of PE may be subtle and difficult to elucidate, and symptomatic and asymptomatic PE appear to have a similar prognosis.[35] We will enroll both patients with single and multiple isolated SSPE because there is no convincing evidence that these conditions are prognostically different.

The exclusion criteria were selected based on a high early risk of VTE recurrence (≥8%), the presence of cardiopulmonary compromise (i.e. hypotension or hypoxemia), contraindications to anticoagulant treatment with rivaroxaban, and a high risk of confounding (**Table 2**).[4,14,36-44] All exclusion criteria are listed in **Table 2**. Because PE usually originates from a thrombus in the deep leg veins,[45] potentially eligible patients systematically receive a single bilateral whole-leg compression ultrasonography (CUS) examination to exclude concomitant DVT, which is a well-known prognostic factor for mortality in patients with PE and represents an absolute indication for therapeutic anticoagulation.[46] If a proximal or distal DVT (with an incompressible distal vein diameter of ≥5mm)[47] is detected, patients are excluded from study participation and treated at the discretion of their managing physician. We cannot fully exclude the small possibility that an eligible patient with SSPE in whom the presence of a leg vein DVT was ruled out by a bilateral whole-leg CUS may have a

For peer review only - http://bmjopen.bmj.com/site/about/guidelines.xhtml

concomitant isolated iliac vein thrombosis or a thrombosis at an unusual site (e.g., in the cerebral, splanchnic, or ovarian veins). However, these are rare thrombotic conditions that often occur in specific clinical situations representing study exclusion criteria (cancer, pregnancy, puerperium).[48] The risk that such patients would present with an isolated SSPE is even lower. Although whole-leg CUS is not an effective method to exclude an isolated iliac vein thrombosis, a meta-analysis of randomized trials and prospective management studies has convincingly shown that in patients with suspected DVT in whom DVT has been excluded by a single whole-leg CUS and in whom anticoagulation is withheld, the risk of recurrent VTE is very low.[49]

Intervention

Patients in the intervention group receive clinical surveillance and a matching rivaroxaban placebo orally using the same dosing schedule, frequency of administration, and duration of treatment as for rivaroxaban (see below). Clinical surveillance is done during the follow-up interviews by phone or in-person, depending on local practice. At each contact, trained study personnel complete an assessment of symptoms and review for suspected recurrent VTE and bleeding. If patients report symptoms or signs suggestive of recurrent VTE or significant bleeding, they are asked to present immediately to an ED for evaluation.

Control

Patients who are assigned to the anticoagulation group receive oral rivaroxaban 15 mg twice daily for the first 21 days, followed by 20 mg once daily for the remaining 90-day study period. After completion of the treatment period, the study drug is discontinued. The same surveillance schedule as in the intervention group is used.

Study Procedures

For peer review only - http://bmjopen.bmj.com/site/about/guidelines.xhtml

Patients are screened for eligibility if they receive a CTPA in the emergency department or within 72 hours of hospitalization to rule-in or rule-out PE, or if they present to an outpatient service within 72 hours of SSPE diagnosis. Eligibility criteria are assessed for all consecutive patients with suspected SSPE, and potentially eligible patients are asked to provide informed consent by investigators or their delegates (see Supplementary File 1 for an English language example of the informed consent form). For those who consent to participate, a bilateral whole-leg CUS is performed by qualified examiners (e.g. radiologists, vascular specialists, or emergency physicians). All eligible and consenting patients without DVT are randomized to receive clinical surveillance or anticoagulation with rivaroxaban, and trained study personnel collect baseline data by reviewing medical records, medication lists, laboratory results that have been obtained as part of routine care, radiology reports, and by patient interview (**Table 3**). After provision of the assigned study medication, the participants are asked to immediately start with the treatment. In addition, all participants receive a patient diary consisting of two parts: the first contains important information about the study, and the second part consists of a diary to record dates and type of outcomes in order to minimize recall bias (**Table 3**).

Participants are followed for 90 days using phone or in-person interviews at 10, 30, and 90 days after randomization (Supplementary File 2). Trained study personnel contacts patients, family members, and/or primary care physicians and review medical charts to obtain information about outcome events. At the end of the treatment period, patients are instructed to return the medication bottles and the patient diary. Drug-adherence is assessed by counting the pills in the returned medication bottles (**Table 3**).

Data Collection and Quality

Data are collected using an electronic database (secuTrial®). The following measures are implemented to ensure optimal data quality and completeness: 1) training of study personnel in the methods of data abstraction, patient inquiry, and data recording, 2) recording of study data on

For peer review only - http://bmjopen.bmj.com/site/about/guidelines.xhtml

standardized electronic case report forms (eCRFs), 3) operations manual providing information on definitions and acceptable data sources for all variables, 4) central data monitoring with generation of statistical reports and individual data checks, and 5) risk based on-site monitoring (i.e., based on key performance indicators such as inappropriate recruitment rate, change of principal investigator, high number of queries raised by central data monitoring, high number of protocol deviations, etc.).

Criteria for Discontinuation of the Study Medication and Unblinding

In participants requiring prophylactic or therapeutic anticoagulation during the study period for reasons other than the index SSPE or if treatment with another prohibited agent is necessary (i.e. strong CYP3A4 inhibitors or inducers, dual antiplatelet therapy, GP IIb/IIIa inhibitors), the study drug should be temporarily interrupted and restarted as soon as possible following discontinuation of the prohibited medication. Patients who develop any condition requiring permanent therapeutic anticoagulation (e.g., atrial fibrillation) should permanently discontinue the study drug. If an invasive procedure or surgical intervention is required, the study medication should be stopped at least 24 hours prior to an elective intervention, or immediately for emergency procedures. If objectively confirmed recurrent VTE or pregnancy is diagnosed during the treatment period, the study drug should be discontinued and treatment allocation unblinded. Similarly, in case of major bleeding, the study drug should be stopped, and unblinding may be necessary if emergency anticoagulation reversal is indicated.

Primary and Secondary Outcomes

The primary (efficacy) outcome is the proportion of recurrent, clinically symptomatic, objectively confirmed VTE within 90 days of randomization, defined as recurrent PE or lower limb DVT.[50,51] The objective diagnostic criterion for PE, based on available radiographic reports, is a new intraluminal filling defect on CTPA or pulmonary angiography; a perfusion defect involving at least 75% of a segment, with corresponding normal ventilation (i.e., high probability lung scan); the

For peer review only - http://bmjopen.bmj.com/site/about/guidelines.xhtml

confirmation of new PE at autopsy; or objectively confirmed proximal DVT of the lower extremity in patients with symptoms of PE. The objective diagnosis of DVT is the non-compressibility of a venous segment on CUS or an intraluminal filling defect on contrast venography. Because compression of iliac veins and the inferior vena cava may be technically difficult, additional diagnostic criteria for iliac and caval DVT also include abnormal duplex flow patterns compatible with thrombosis or an intraluminal filling defect on computed tomography or magnetic resonance imaging venography.[52] Both proximal and distal DVTs are considered.

Separate secondary (safety) outcomes include the proportion of clinically significant bleeding and all-cause mortality 90 days following randomization. Clinically significant bleeding is a composite endpoint of major and clinically relevant non-major bleeding. Major bleeding is defined as fatal bleeding, symptomatic bleeding at critical sites (intracranial, intraspinal, intraocular, retroperitoneal, intraarticular, pericardial, or intramuscular with compartment syndrome), or bleeding with a reduction of hemoglobin ≥20 g/L, or bleeding leading to transfusion of ≥2 units of packed red blood cells according to the definition of the International Society on Thrombosis and Haemostasis.[53] Clinically relevant non-major bleeding is defined as overt bleeding that does not meet criteria for major bleeding but is associated with a medical intervention, unscheduled physician contact (visit or telephone call), temporary cessation of the study drug, pain, or impairment of activities of daily life.[54]

Information about the date, type, and circumstances of outcome events is obtained from patients, family members, and/or health care providers during the follow-up interviews or by reviewing medical charts. In patients who experience recurrent VTE, the radiographic report and images confirming VTE recurrence are obtained. For patients who died during follow-up, the cause of death based on medical reports, death certificates, and autopsy reports (if available) is recorded. All medical outcome events are reviewed and adjudicated by a committee of 3 independent clinical experts unaware of treatment assignment. Based on available information, death is adjudicated as PE-related, due to major bleeding (any death following an intracranial hemorrhage or a bleeding episode leading to hemodynamic deterioration), due to another cause, or due to an undetermined

For peer review only - http://bmjopen.bmj.com/site/about/guidelines.xhtml

cause. Death is considered PE-related in the following situations: a) autopsy-confirmed PE in the absence of another more likely cause of death, b) objectively confirmed PE within the last 48 hours before death in the absence of another more likely cause of death, or c) PE is not objectively confirmed, but is most likely the main cause of death.[55] Final classification of all medical outcomes is based on the full consensus of the committee.

Ancillary Outcomes

Ancillary outcomes include health-related quality of life and functional status, both important patient-centered outcomes, and medical resource utilization, an economically relevant efficiency measure. Disease-specific, health-related quality of life at baseline, 30 and 90 days after the index PE is assessed using the Pulmonary Embolism Quality of Life (PEmb-QoL) questionnaire.[56,57] The PEmb-QoL is a validated, self-administered 40-item questionnaire to quantify quality of life in patients having experienced PE. Functional status is measured at 90 days after randomization using the post-VTE functional status scale.[58,59] This scale has been proposed to assess functional limitations after VTE, covering aspects of daily life (including limitations in usual activity and changes in lifestyle) that are affected by the consequences of VTE and its complications. The assessment is done using a short, structured interview 90 days after randomization.

The following measures of medical resource utilization and productivity are assessed:[44,60] initial length of stay (LOS), subsequent overall hospitalizations as well as overall ED and physician outpatient visits within 90 days of randomization, and time to return to work in workers or usual activities (e.g., household) in non-workers. Information on these outcomes are obtained from the participant during the follow-up interviews, by interview of the patient's primary care physician, and by hospital chart review. Subsequent health care contacts are classified as potentially related to VTE if a patient had chest or leg symptoms or signs (dyspnea, chest pain, pleural effusion, or leg pain or swelling), or bleeding complications.

For peer review only - http://bmjopen.bmj.com/site/about/guidelines.xhtml

Withdrawals and Loss to Follow-Up

Given the low treatment and follow-up burden and the short follow-up period of 90 days, we expect that completeness of follow-up data collection will be close to 100% based on prior experience.[44] A patient who withdraws consent for follow-up at 10 or 30 days may still agree with passive follow-up (i.e. that study personnel may collect follow-up information from medical records of the participant's primary care physician) or to continue with the assessment at 90 days, if given the option. If study withdrawal occurs, data collected up to the time of withdrawal is used in a coded manner. If a participant is lost to follow-up, primary care physicians and surrogates are contacted, and the hospital records are consulted to obtain information about primary and secondary outcomes and survival status.

Post-hoc Evaluation of Radiologic Inter-Observer Agreement

CTPA images undergo central review at the Bern University Hospital by a panel of two experienced thoracic radiologists blinded to the interpretation of the radiologist at the enrolling site. Based on the consensus of this panel, the initial CTPA readings are classified into 3 categories: 1) presence of isolated SSPE (true-positive isolated SSPE), 2) absence of any PE (false-positive isolated SSPE), and 3) presence of segmental, lobar, or central PE (false-negative isolated SSPE). The panel also evaluates the technical quality of the CTPA examination (adequacy of opacification, breathing artifacts) and the number of filling defects (i.e., single vs. multiple isolated SSPE). Confirmation of the SSPE diagnosis prior to enrolling the patient is logistically not feasible due to the short timeline between diagnosis and enrolment, and it would not reflect real-world practice, thus limiting the external validity of our study results. Therefore, the central review of CTPA images is done post-hoc in 6-month batches.

Sample Size Calculation

For peer review only - http://bmjopen.bmj.com/site/about/guidelines.xhtml

Assumptions on VTE recurrence risk are based on data from 127 low-risk patients with isolated SSPE who received anticoagulants (warfarin or low-molecular-weight heparin), showing a VTE recurrence risk of 0.8% at 90 days after diagnosis.[23] We chose an absolute non-inferiority margin of 3.5% on the basis of recruitment feasibility, clinical acceptability, and previous studies. This corresponds to a difference which is considered acceptable by most physicians and patients for the following reasons. First, our margin is within the range of the 3-month VTE recurrence proportion (0.5-5%) below which thrombosis specialists would not initiate anticoagulation for PE.[61] Second, the definition of a clinically acceptable non-inferiority margin for recurrent VTE must also take into account the potential benefits of withholding anticoagulation, i.e. the substantially lower risk of clinically significant bleeding (<1% versus 7% within 3 months for patients receiving anticoagulants).[5,23,25,62] Indeed, a patient group with PE who was involved in the trial planning process indicated that given the bleeding risk associated with anticoagulants, a VTE recurrence proportion of <5% seemed acceptable. Finally, similar non-inferiority margins (3-5%) have been used in key studies comparing different drug treatment regimens and inpatient vs. outpatient management for acute VTE.[42,44,63-67]

To determine the sample size, we used a Monte-Carlo simulation approach based on an Agresti-Caffo confidence interval (CI) for risk difference.[68] Assuming a baseline VTE recurrence proportion of 1.0% at 90 days in both treatment groups, an absolute margin of 3.5% defining non-inferiority for clinical surveillance, and a sampling ratio of 1:1 allowing 5% attrition (dropouts, including patients who died from non-VTE-related causes) in each group during 90 days, we estimated that 276 patients (138 per group) would result in at least 80% power to establish non-inferiority at an one-sided type I error of 5%.

Planned Statistical Analyses

As recommended by the CONSORT statement for non-inferiority trials,[69] we will perform both intention-to-treat (ITT) and per-protocol (PP) analyses. For the primary outcome, ITT and PP analyses

For peer review only - http://bmjopen.bmj.com/site/about/guidelines.xhtml

should reach the same conclusion to consider results to be robust. For the remaining outcomes, the ITT analysis will be the primary analysis, the PP analysis a secondary analysis.

In the ITT analysis, all randomized patients will be analyzed within the treatment group to which they were randomized. In the PP analysis, patients with protocol violations will be excluded (crossover to study treatment from other group; patients receiving a different type or dose of anticoagulation than requested by the study protocol; patients with missing primary outcome data; patients violating relevant eligibility criteria; patients stopping study treatment within 1 month after randomization, or patients in whom the diagnosis of isolated SSPE was refuted by the central CTPA review panel).

We will describe the prevalence of recurrent VTE, clinically significant bleeding (including its individual components, major and clinically relevant non-major bleeding), and all-cause mortality at 90 days after randomization with 95% Wilson CIs by treatment group. Non-inferiority of the primary outcome (VTE recurrence) among patients in the clinical surveillance vs. the anticoagulation group will be assessed based on the Agresti-Caffo CI for risk difference.[68] If the upper limit of the one-sided 95% Agresti-Caffo CI will be lower than the pre-specified non-inferiority margin, clinical surveillance will be considered non-inferior to anticoagulation. For secondary outcomes, we will calculate the risk difference with a two-sided 95% Agresti-Caffo CI and compare groups using an exact binomial test.[68]

PEmb-QoL dimension scores at 30 and 90 days as well as the change in scores from baseline will be presented by treatment group as means with 95% CIs. The change in dimension scores and the differences in the change between groups will be analyzed using a repeated-measures, linear mixed-effects model adjusted for the respective baseline value. Functional status, which is measured on a scale from 0 (no functional limitations) to 5 (death) at 90 days, will be presented as median and interquartile range and compared between groups using the non-parametric Wilcoxon rank-sum test. Count data (subsequent hospitalizations, outpatient visits) will be presented by treatment group as rate with an exact 95% Poisson CI and compared using a rate ratio and exact p-value. Time-to-event outcomes (initial LOS, time to return to work/usual activities) will be presented as medians and

For peer review only - http://bmjopen.bmj.com/site/about/guidelines.xhtml

interquartile ranges. For LOS, we will compare groups using the Wilcoxon rank-sum test. For return to work, we will display Kaplan-Meier curves and compare groups by the log-rank test. An alpha level of <0.05 will define statistical significance.

In secondary analyses, we will calculate the cumulative incidence and the difference in cumulative incidence of VTE recurrence and clinically significant bleeding, correcting for withdrawals and losses to follow-up by censoring and for death unrelated to VTE recurrence or bleeding as a competing event. All-cause mortality will be assessed likewise, however a competing event does not apply. In a further secondary analysis, we will use model-based approaches for all outcomes. For time-to-event outcomes (VTE recurrence, clinically significant bleeding, LOS, return to work/usual activity), we will use competing risk regression according to Fine and Gray,[70] accounting for non-VTE/non-bleeding-related death or all-cause death as a competing event. For mortality, we will use Cox regression. For count data, we will use a negative binomial model, and also consider zero-inflation. In case of heterogeneity across sites, we will adjust models for site using random-effects models.

Data Monitoring and Interim Safety Analysis

An independent Data and Safety Monitoring Board (DSMB) consisting of 3 members unaffiliated with any of the participating institutions will evaluate unblinded interim safety results after 100 and 190 randomized patients have completed the 90-day follow-up. To monitor recurrent VTE in the intervention group, formal interim analyses will be performed using a Bayesian approach. Based on literature,[23] we expect a frequency of VTE recurrence of 1.0% (0-4.5%). We regard a frequency of >5% in the surveillance group as clinically unacceptable. We will calculate the prior probability distribution of events based on the expected frequency as well as the posterior probability distribution using actually observed data at each interim analysis. Based on the posterior distribution, we will calculate the probability that the true proportion of VTE recurrence in the surveillance group exceeds the threshold of unacceptable frequency. If the probability of exceedance

For peer review only - http://bmjopen.bmj.com/site/about/guidelines.xhtml

is >70%, the surveillance arm will be considered inferior and the DSMB will recommend to stop the trial. The final decision will lie with the Steering Committee. This procedure is conservative regarding the type-I error rate; therefore, we will not adjust the significance level in the final analysis.

Patient and Public Involvement

We have partnered with a group of patients who had recently experienced PE in the planning process of this trial, including the selection of patient-centered outcomes, the establishment of a safe surveillance schedule, and the determination of a clinically acceptable non-inferiority margin. Patients are not involved in the recruitment and conduct of the study or the dissemination of study results.

Data Sharing

After publication of the study results, a de-identified patient-level data set relating to the primary publication along with the latest version of the study protocol, the informed consent form, the statistical analysis plan, the analysis code, and the data management plan of the study will be made publicly available in the *Bern Open Repository and Information System* (*BORIS) Research Data*.

Ethics and Dissemination

This trial is conducted in accordance with the Declaration of Helsinki, the International Council on Harmonization Good Clinical Practice (ICH-GCP) guidelines, and all applicable national legal and regulatory requirements. Authorization by the local Ethics Committees and Swissmedic has been obtained in Switzerland, and the submission process to the relevant Dutch and Canadian Ethics Committees and regulatory authorities is ongoing. All changes in research activity or unanticipated problems involving risks to human subjects will be reported promptly to the Ethics Committees. All participating investigators/institutions will permit study-related monitoring, audits, Ethics Committee

For peer review only - http://bmjopen.bmj.com/site/about/guidelines.xhtml

reviews, and regulatory inspections, and will provide direct access to source documents and data. Protection of confidentiality is ensured according to regulatory requirements.

Study personnel informs eligible patients with a diagnosis of isolated SSPE about all aspects of the study participation, including the goals, procedures, and potential risks/benefits associated with the study. Potential participants are informed that the decision to participate in the study is entirely voluntary and that they may withdraw from the study at any time, with no effect on their current or future treatment. Written informed consent is obtained from all eligible patients prior to any study-related procedure, including bilateral whole-leg CUS. The primary study results will be presented at scientific conferences and published in a peer-reviewed medical journal. We also plan local presentations for physicians at the participating sites. Participants will receive a letter with the study results explained in lay language. Further public dissemination of the results is planned through publications in the lay press and via social media.

Conclusion

The SAFE-SSPE trial addresses an important gap of knowledge, the optimal management of SSPE, and represents the first randomized, direct comparison of clinical surveillance alone versus anticoagulant treatment for low-risk patients with isolated SSPE. As the number of SSPE patients who may eventually receive anticoagulant treatment is likely to rise in the future with the further dissemination and advancement of CTPA technology, a strong scientific basis for withholding anticoagulation in low risk patients with SSPE is urgently needed. The results of this trial have the potential to improve quality and efficiency of care by reducing bleeding episodes and resource utilization and increasing health-related quality of life.

Current Status of the SAFE-SSPE trial

Patient recruitment has started in May 2020; by July 2020, 6 patients have been enrolled in the trial. Follow-up of the last participant is expected to be completed in February 2024.

For peer review only - http://bmjopen.bmj.com/site/about/guidelines.xhtml

Authors' Contributions

Study concept and design: DA, MH, MC, FAK, JHB, MR, AL, CB

Drafting of the manuscript: CB, DA

Critical revision of the manuscript for important intellectual content: MH, MC, FAK, JHB, MR, AL, JM, MP, DR

Statistical analysis plan: AL

Obtained funding: DA, MR, JHB

Administrative, technical, or material support: MP, JM, DR, CB

Supervision: DA

Acknowledgements

We would like to thank Sven Trelle, Director of the CTU Bern (Clinical Trials Unit) for his methodological inputs and organizational work concerning this study.

Funding Statement

This study is supported by an Investigator Initiated Clinical Trials (IICT) grant of the Swiss National Science Foundation (SNSF 33IC30_185616). The study medication was provided free of charge by Bayer AG.

Competing Interests Statement

Dr. Carrier reports grants from Leo Pharma, BMS, Pfizer, personal fees from Bayer, BMS, Pfizer, Sanofi, Leo Pharma, Servier, outside the submitted work. All other authors declare that they have no competing interest with regard to the intellectual concern and proprietary of affairs. The funding source had no role in the design of this study and does not have any role in the conduct, the collection, management, analysis, and interpretation of the data, the writing of the manuscript, or the decision to submit the results for publication.

For peer review only

For peer review only - http://bmjopen.bmj.com/site/about/guidelines.xhtml

Conveyance and Peer Review

This trial protocol has been peer-reviewed for approval of funding and ethical approval prior to submission.

Roles and Responsibilities

Sponsor-Investigator:

Prof. Drahomir Aujesky, MD, MSc, Department of General Internal Medicine, Inselspital, Bern University Hospital, University of Bern, Freiburgstrasse, CH-3010 Bern, Switzerland. Phone: +41 (0)31 632 88 84; e-mail: drahomir.aujesky@insel.ch

Role: sponsor-investigator, coordinating principal investigator, chair of the Steering Committee; responsible for the study design, overseeing the management of the study, interpretation of the data, and writing of the manuscript.

Legal Representative of the Sponsor in the Netherlands:

Frederikus A. Klok, MD, PhD, FESC, Leiden University Medical Center, Albinusdreef 2, NL-2333 ZA Leiden, The Netherlands. Phone: +31 71 529 8127; e-mail: F.A.Klok@lumc.nl

role: Steering Committee member, venous thromboembolism expert, site investigator, recruitment, legal representative of the sponsor in the Netherlands

Legal representative of the Sponsor in Canada:

Prof. Marc Carrier, MD, MSc, Department of Medicine, The Ottawa Hospital Research Institute at the University of Ottawa, CA-Ottawa, K1H8L6, Canada. Phone: +1 613 737 8899, e-mail: mcarrier@toh.ca

role: Steering Committee member, venous thromboembolism expert, site investigator, recruitment, legal representative of the sponsor in Canada

Steering Committee:

For peer review only - http://bmjopen.bmj.com/site/about/guidelines.xhtml

Chair: Prof. Drahomir Aujesky, MD, MSc

Steering committee members: Prof. Jürg-Hans Beer, MD; Prof. Marc Carrier, MD, MSc; Dr. Frederikus A. Klok, MD, PhD, FESC; Prof. Marc Righini, MD

The study steering committee is responsible for the overall supervision of the trial and regularly meets to discuss scientific and logistic aspects of the trial.

Monitoring Institution and Data Management:

CTU Bern (Clinical Trials Unit), University of Bern, Mittelstrasse 43, CH-3012 Bern, Switzerland.

Phone: +41 (0)31 631 35 04; e-mail: sven.trelle@ctu.unibe.ch

Date Safety Monitoring Board:

An independent Data Safety Monitoring Board (DSMB) evaluates unblinded VTE outcomes on a regular basis and make recommendations to the Steering Committee to amend or terminate the trial should any safety concerns arise. The DSMB includes 3 members unaffiliated with Bern University Hospital or any research partners: 2 experts in the area of VTE, and 1 expert in statistics and methodology of randomized trials.

Other Relevant Committees and Institutions:

The study is managed and coordinated at the Inselspital, Bern University Hospital.

An independent outcome adjudication committee consisting of 3 clinical experts at the Inselspital, Bern University Hospital, is responsible for adjudication of the primary and secondary outcomes events in a blinded fashion.

A central CT review panel, consisting of two experienced thoracic radiologists at the Inselspital, Bern, University Hospital, centrally reviews all CTPA images.

The Hospital Pharmacy of the Inselspital, Bern University Hospital, is in charge of the pharmaceutical tasks.

For peer review only - http://bmjopen.bmj.com/site/about/guidelines.xhtml

References

1. Di Nisio M, van Es N, Buller HR. Deep vein thrombosis and pulmonary embolism. *Lancet.* 2016;388(10063):3060-3073.
2. Huisman MV, Barco S, Cannegieter SC, et al. Pulmonary embolism. *Nat Rev Dis Primers.* 2018;4:18028.
3. Castellucci LA, Cameron C, Le Gal G, et al. Clinical and safety outcomes associated with treatment of acute venous thromboembolism: a systematic review and meta-analysis. *JAMA.* 2014;312(11):1122-1135.
4. Kearon C, Akl EA, Ornelas J, et al. Antithrombotic Therapy for VTE Disease: CHEST Guideline and Expert Panel Report. *Chest.* 2016;149(2):315-352.
5. Buller HR, Prins MH, Lensin AW, et al. Oral rivaroxaban for the treatment of symptomatic pulmonary embolism. *N Engl J Med.* 2012;366(14):1287-1297.
6. Jun M, Lix LM, Durand M, et al. Comparative safety of direct oral anticoagulants and warfarin in venous thromboembolism: multicentre, population based, observational study. *Bmj.* 2017;359:j4323.
7. Linkins LA, Choi PT, Douketis JD. Clinical impact of bleeding in patients taking oral anticoagulant therapy for venous thromboembolism: a meta-analysis. *Ann Intern Med.* 2003;139(11):893-900.
8. Scherz N, Mean M, Limacher A, et al. Prospective, multicenter validation of prediction scores for major bleeding in elderly patients with venous thromboembolism. *J Thromb Haemost.* 2013;11(3):435-443.
9. Wiener RS, Schwartz LM, Woloshin S. Time trends in pulmonary embolism in the United States: evidence of overdiagnosis. *Arch Intern Med.* 2011;171(9):831-837.
10. Auer RC, Schulman AR, Tuorto S, et al. Use of helical CT is associated with an increased incidence of postoperative pulmonary emboli in cancer patients with no change in the number of fatal pulmonary emboli. *J Am Coll Surg.* 2009;208(5):871-878; discussion 878-880.

For peer review only - http://bmjopen.bmj.com/site/about/guidelines.xhtml

11. Ikesaka R, Carrier M. Clinical significance and management of subsegmental pulmonary embolism. *J Thromb Thrombolysis.* 2015;39(3):311-314.

12. Carrier M, Righini M, Wells PS, et al. Subsegmental pulmonary embolism diagnosed by computed tomography: incidence and clinical implications. A systematic review and meta-analysis of the management outcome studies. *J Thromb Haemost.* 2010;8(8):1716-1722.

13. Carrier M, Righini M, Le Gal G. Symptomatic subsegmental pulmonary embolism: what is the next step? *J Thromb Haemost.* 2012;10(8):1486-1490.

14. Stein PD, Goodman LR, Hull RD, Dalen JE, Matta F. Diagnosis and management of isolated subsegmental pulmonary embolism: review and assessment of the options. *Clin Appl Thromb Hemost.* 2012;18(1):20-26.

15. van der Pol LM, Bistervels IM, van Mens TE, et al. Lower prevalence of subsegmental pulmonary embolism after application of the YEARS diagnostic algorithm. *Br J Haematol.* 2018.

16. Barco S, Mahmoudpour SH, Valerio L, et al. Trends in mortality related to pulmonary embolism in the European Region, 2000-15: analysis of vital registration data from the WHO Mortality Database. *Lancet Respir Med.* 2020;8(3):277-287.

17. Stein PD, Fowler SE, Goodman LR, et al. Multidetector computed tomography for acute pulmonary embolism. *N Engl J Med.* 2006;354(22):2317-2327.

18. Ghanima W, Nielssen BE, Holmen LO, Witwit A, Al-Ashtari A, Sandset PM. Multidetector computed tomography (MDCT) in the diagnosis of pulmonary embolism: interobserver agreement among radiologists with varied levels of experience. *Acta Radiol.* 2007;48(2):165-170.

19. Gurney JW. No fooling around: direct visualization of pulmonary embolism. *Radiology.* 1993;188(3):618-619.

20. Schoepf UJ, Costello P. CT angiography for diagnosis of pulmonary embolism: state of the art. *Radiology.* 2004;230(2):329-337.

For peer review only

21. Carrier M, Klok FA. Symptomatic subsegmental pulmonary embolism: to treat or not to treat? *Hematology Am Soc Hematol Educ Program.* 2017;2017(1):237-241.

22. Cha SI, Shin KM, Lee JW, et al. Clinical characteristics of patients with peripheral pulmonary embolism. *Respiration.* 2010;80(6):500-508.

23. Donato AA, Khoche S, Santora J, Wagner B. Clinical outcomes in patients with isolated subsegmental pulmonary emboli diagnosed by multidetector CT pulmonary angiography. *Thromb Res.* 2010;126(4):e266-270.

24. Le Gal G, Righini M, Parent F, van Strijen M, Couturaud F. Diagnosis and management of subsegmental pulmonary embolism. *J Thromb Haemost.* 2006;4(4):724-731.

25. Mehta D, Barnett M, Zhou L, et al. Management and outcomes of single subsegmental pulmonary embolus: a retrospective audit at North Shore Hospital, New Zealand. *Intern Med J.* 2014;44(9):872-876.

26. Bariteau A, Stewart LK, Emmett TW, Kline JA. Systematic review and meta-analysis of outcomes of patients with subsegmental pulmonary embolism with and without anticoagulation treatment. *Acad Emerg Med.* 2018.

27. Eyer BA, Goodman LR, Washington L. Clinicians' response to radiologists' reports of isolated subsegmental pulmonary embolism or inconclusive interpretation of pulmonary embolism using MDCT. *AJR Am J Roentgenol.* 2005;184(2):623-628.

28. Douketis JD, Foster GA, Crowther MA, Prins MH, Ginsberg JS. Clinical risk factors and timing of recurrent venous thromboembolism during the initial 3 months of anticoagulant therapy. *Arch Intern Med.* 2000;160(22):3431-3436.

29. Pesavento R, de Conti G, Minotto I, et al. The value of 64-detector row computed tomography for the exclusion of pulmonary embolism. *Thromb Haemost.* 2011;105(5):901-907.

For peer review only - http://bmjopen.bmj.com/site/about/guidelines.xhtml

30. Raslan IA, Chong J, Gallix B, Lee TC, McDonald EG. Rates of Overtreatment and Treatment-Related Adverse Effects Among Patients With Subsegmental Pulmonary Embolism. *JAMA Intern Med.* 2018;178(9):1272-1274.

31. Yoo HH, Queluz TH, El Dib R. Anticoagulant treatment for subsegmental pulmonary embolism. *Cochrane Database Syst Rev.* 2016(1):CD010222.

32. Chan AW, Tetzlaff JM, Altman DG, et al. SPIRIT 2013 statement: defining standard protocol items for clinical trials. *Ann Intern Med.* 2013;158(3):200-207.

33. Peiman S, Abbasi M, Allameh SF, Asadi Gharabaghi M, Abtahi H, Safavi E. Subsegmental pulmonary embolism: A narrative review. *Thromb Res.* 2016;138:55-60.

34. Schoellnast H, Deutschmann HA, Fritz GA, Stessel U, Schaffler GJ, Tillich M. MDCT angiography of the pulmonary arteries: influence of iodine flow concentration on vessel attenuation and visualization. *AJR Am J Roentgenol.* 2005;184(6):1935-1939.

35. Klok FA, Huisman MV. Management of incidental pulmonary embolism. *Eur Respir J.* 2017;49(6).

36. van der Hulle T, den Exter PL, Planquette B, et al. Risk of recurrent venous thromboembolism and major hemorrhage in cancer-associated incidental pulmonary embolism among treated and untreated patients: a pooled analysis of 926 patients. *J Thromb Haemost.* 2016;14(1):105-113.

37. Kearon C, Ageno W, Cannegieter SC, et al. Categorization of patients as having provoked or unprovoked venous thromboembolism: guidance from the SSC of ISTH. *J Thromb Haemost.* 2016;14(7):1480-1483.

38. Prandoni P, Noventa F, Ghirarduzzi A, et al. The risk of recurrent venous thromboembolism after discontinuing anticoagulation in patients with acute proximal deep vein thrombosis or pulmonary embolism. A prospective cohort study in 1,626 patients. *Haematologica.* 2007;92(2):199-205.

For peer review only - http://bmjopen.bmj.com/site/about/guidelines.xhtml

39. Iorio A, Kearon C, Filippucci E, et al. Risk of recurrence after a first episode of symptomatic venous thromboembolism provoked by a transient risk factor: a systematic review. *Arch Intern Med.* 2010;170(19):1710-1716.

40. van der Hulle T, Tan M, den Exter PL, et al. Recurrence risk after anticoagulant treatment of limited duration for late, second venous thromboembolism. *Haematologica.* 2015;100(2):188-193.

41. Moores LK. Are We Overtreating Isolated Subsegmental Pulmonary Embolism?: First Do No Harm. *JAMA Intern Med.* 2018;178(9):1274-1275.

42. Agnelli G, Buller HR, Cohen A, et al. Oral apixaban for the treatment of acute venous thromboembolism. *N Engl J Med.* 2013;369(9):799-808.

43. Giugliano RP, Ruff CT, Braunwald E, et al. Edoxaban versus warfarin in patients with atrial fibrillation. *N Engl J Med.* 2013;369(22):2093-2104.

44. Aujesky D, Roy PM, Verschuren F, et al. Outpatient versus inpatient treatment for patients with acute pulmonary embolism: an international, open-label, randomised, non-inferiority trial. *Lancet.* 2011;378(9785):41-48.

45. Kearon C. Natural history of venous thromboembolism. *Circulation.* 2003;107(23 Suppl 1):I22-30.

46. Jimenez D, Aujesky D, Diaz G, et al. Prognostic significance of deep vein thrombosis in patients presenting with acute symptomatic pulmonary embolism. *Am J Respir Crit Care Med.* 2010;181(9):983-991.

47. Righini M, Le Gal G, Aujesky D, et al. Complete venous ultrasound in outpatients with suspected pulmonary embolism. *J Thromb Haemost.* 2009;7(3):406-412.

48. Mimier MK, Janczak DT, McBane RD, Houghton DE, Wysokinski WE. Thrombosis of atypical location: how to treat patients in the era of direct oral anticoagulants? *Pol Arch Intern Med.* 2018;128(10):604-608.

For peer review only - http://bmjopen.bmj.com/site/about/guidelines.xhtml

49. Johnson SA, Stevens SM, Woller SC, et al. Risk of deep vein thrombosis following a single negative whole-leg compression ultrasound: a systematic review and meta-analysis. *JAMA.* 2010;303(5):438-445.

50. Buller HR, Cohen AT, Davidson B, et al. Idraparinux versus standard therapy for venous thromboembolic disease. *N Engl J Med.* 2007;357(11):1094-1104.

51. Lim W, Le Gal G, Bates SM, et al. American Society of Hematology 2018 guidelines for management of venous thromboembolism: diagnosis of venous thromboembolism. *Blood Adv.* 2018;2(22):3226-3256.

52. Mean M, Righini M, Jaeger K, et al. The Swiss cohort of elderly patients with venous thromboembolism (SWITCO65+): rationale and methodology. *J Thromb Thrombolysis.* 2013;36(4):475-483.

53. Schulman S, Kearon C. Definition of major bleeding in clinical investigations of antihemostatic medicinal products in non-surgical patients. *J Thromb Haemost.* 2005;3(4):692-694.

54. Raskob GE, Spyropoulos AC, Zrubek J, et al. The MARINER trial of rivaroxaban after hospital discharge for medical patients at high risk of VTE. Design, rationale, and clinical implications. *Thromb Haemost.* 2016;115(6):1240-1248.

55. Tritschler T, Kraaijpoel N, Girard P, et al. Definition of pulmonary embolism-related death and classification of the cause of death in venous thromboembolism studies: communication from the SSC of the ISTH. *J Thromb Haemost.* in press.

56. Cohn DM, Nelis EA, Busweiler LA, Kaptein AA, Middeldorp S. Quality of life after pulmonary embolism: the development of the PEmb-QoL questionnaire. *J Thromb Haemost.* 2009;7(6):1044-1046.

57. Klok FA, Cohn DM, Middeldorp S, et al. Quality of life after pulmonary embolism: validation of the PEmb-QoL Questionnaire. *J Thromb Haemost.* 2010;8(3):523-532.

58. Klok FA, Barco S, Siegerink B. Measuring functional limitations after venous thromboembolism: A call to action. *Thromb Res.* 2019;178:59-62.

For peer review only - http://bmjopen.bmj.com/site/about/guidelines.xhtml

59. Boon G, Barco S, Bertoletti L, et al. Measuring functional limitations after venous thromboembolism: Optimization of the Post-VTE Functional Status (PVFS) Scale. *Thromb Res.* 2020;190:45-51.

60. Yealy DM, Auble TE, Stone RA, et al. Effect of increasing the intensity of implementing pneumonia guidelines: a randomized, controlled trial. *Ann Intern Med.* 2005;143(12):881-894.

61. Carrier M, Kimpton M, G LEG, et al. The management of a sub-segmental pulmonary embolism: a cross-sectional survey of Canadian thrombosis physicians. *J Thromb Haemost.* 2011;9(7):1412-1415.

62. Mauri L, D'Agostino RB, Sr. Challenges in the Design and Interpretation of Noninferiority Trials. *N Engl J Med.* 2017;377(14):1357-1367.

63. Levine M, Gent M, Hirsh J, et al. A comparison of low-molecular-weight heparin administered primarily at home with unfractionated heparin administered in the hospital for proximal deep-vein thrombosis. *N Engl J Med.* 1996;334(11):677-681.

64. Koopman MM, Prandoni P, Piovella F, et al. Treatment of venous thrombosis with intravenous unfractionated heparin administered in the hospital as compared with subcutaneous low-molecular-weight heparin administered at home. The Tasman Study Group. *N Engl J Med.* 1996;334(11):682-687.

65. Columbus I, Buller HR, Gent M, et al. Low-molecular-weight heparin in the treatment of patients with venous thromboembolism. *N Engl J Med.* 1997;337(10):657-662.

66. Buller HR, Davidson BL, Decousus H, et al. Fondaparinux or enoxaparin for the initial treatment of symptomatic deep venous thrombosis: a randomized trial. *Ann Intern Med.* 2004;140(11):867-873.

67. Buller HR, Davidson BL, Decousus H, et al. Subcutaneous fondaparinux versus intravenous unfractionated heparin in the initial treatment of pulmonary embolism. *N Engl J Med.* 2003;349(18):1695-1702.

For peer review only - http://bmjopen.bmj.com/site/about/guidelines.xhtml

68. Agresti A, Caffo B. Simple and effective confidence intervals for proportions and differences of proportions result from adding two successes and two failures. *The American Statistician.* 2000;54:280-288.

69. Piaggio G, Elbourne DR, Pocock SJ, Evans SJ, Altman DG, Group C. Reporting of noninferiority and equivalence randomized trials: extension of the CONSORT 2010 statement. *JAMA.* 2012;308(24):2594-2604.

70. Fine JP, Gray RJ. Proportional hazards model for the subdistribution of a competing risk. *J Am Stat Assoc.* 1999;94:496-509.

For peer review only

For peer review only - http://bmjopen.bmj.com/site/about/guidelines.xhtml

Figure Legend

Figure. Overview of the Study Design.

SAFE-SSPE is a randomized, placebo-controlled, parallel-group non-inferiority trial. Low-risk patients with isolated SSPE without concomitant DVT are randomly assigned in a 1:1 ratio to receive placebo ("clinical surveillance group") or anticoagulant treatment with rivaroxaban ("anticoagulation group"). The primary study outcomes are symptomatic recurrent VTE within 90 days of randomization. Secondary outcomes include clinically significant bleeding and all-cause mortality, and ancillary outcomes will be health-related quality of life, functional status, and medical resource utilization. Abbreviations: DVT, deep vein thrombosis; PE, pulmonary embolism; SSPE, subsegmental pulmonary embolism; VTE, venous thromboembolism.

For peer review only

For peer review only - http://bmjopen.bmj.com/site/about/guidelines.xhtml

Table 1. Study Population, Intervention, Control, and Outcomes

**Population**	Consecutive adult low-risk patients with an objective diagnosis of **isolated subsegmental PE** who have no concomitant DVT.
**Intervention**	**Clinical surveillance plus a matching rivaroxaban placebo,** one tablet twice daily for the first 21 days, followed by 1 tablet once daily for the remaining 90-da study period. Clinical surveillance is done at 10, 30 and 90 days following randomization by phone or by in-person visits, depending on local practice. At each contact, trained study personnel complete an assessment of symptoms and review for suspected recurrent VTE and bleeding using a checklist of pre-defined questions. Patients are also instructed to contact study personnel or report to the ED immediately if any symptoms/signs compatible with recurrent VTE or significant bleeding occur.
**Control**	**Clinical surveillance plus anticoagulation with rivaroxaban**, dosed at 15 mg twice daily for the first 21 days, followed by 20 mg once daily for the remaining 90-day study period. The same surveillance schedule as in the clinical surveillance group is used. Patients also receive the same instructions to contact study personnel or report to the ED if any signs or symptoms of VTE or significant bleeding occur.
**Outcomes**	Primary outcome: **recurrent, clinically symptomatic, objectively confirmed VTE** within 90 days of randomization, defined as recurrent fatal or nonfatal PE or lower limb DVT (efficacy).<br>Secondary outcomes: **clinically significant bleeding** and **all-cause mortality** at 90 days of randomization (safety).<br>Ancillary outcomes: **health-related quality of life, functional status, and medical resource utilization** at 90 days of randomization.<br>In a post-hoc analysis, **radiological inter-observer agreement** for SSPE will be assessed.

Abbreviations: DVT, deep vein thrombosis; ED, emergency department; PE, pulmonary embolism; SSPE, subsegmental pulmonary embolism; VTE, venous thromboembolism.

For peer review only

For peer review only - http://bmjopen.bmj.com/site/about/guidelines.xhtml

1 **Table 2. Exclusion Criteria and their Rationale**

Exclusion Criterion	Rationale
1) Presence of leg DVT or upper extremity DVT (subclavian vein or above)	absolute indication for therapeutic anticoagulation
2) Active cancer	high risk of recurrent VTE if left untreated[36]
3) History of ≥1 prior episode of unprovoked VTE (±thrombophilia) [37]	high risk of recurrent VTE after stopping anticoagulation[38-40]
4) Clinical instability (systolic blood pressure <100 mm Hg or arterial oxygen saturation <92% at ambient air) at the time of presentation	risk of clinical deterioration[4,14,41]
5) Active bleeding or at high risk of bleeding (e.g., signs of active bleeding, ischemic stroke during preceding <10 days,[42] major gastrointestinal bleeding during preceding <3 months, intracranial or intraocular bleeding <6 months,[42] major trauma or surgery during preceding <1 month,[42,43] platelets <75,000 per mm^3,[44] or double anti-platelet therapy at the time of enrolment)	contraindication to rivaroxaban
6) Severe renal failure (creatinine clearance <30 ml/min.)	contraindication to rivaroxaban
7) Severe liver insufficiency (Child-Pugh B and C)	contraindication to rivaroxaban
8) Concomitant use of strong CYP3A4 inhibitors (i.e., HIV protease inhibitors [saquinavir, indinavir, ritonavir, nelfinavir, amprenavir, lopinavir, atazanavir, fosamprenavir, tipranavir, darunavir], systemic azole antifungals [i.e., ketoconazole, itraconazole, voriconazole, or posaconazole]) or strong CYP3A4 inducers (i.e., rifampicin, rifabutin, rifapentin, phenytoin, phenobarbital, primidone, carbamazepine, or St. John's Wort)	contraindication to rivaroxaban
9) Known hypersensitivity to rivaroxaban	contraindication to rivaroxaban
10) Need for therapeutic anticoagulation for another reason (atrial fibrillation/flutter, mechanical heart valves, previous VTE, known antiphospholipid antibody syndrome with unprovoked VTE)	randomization to placebo unethical
11) Therapeutic anticoagulation for >72 hours for any reason at the time of screening	could confound study outcomes
12) Hospitalized for >72 hours prior to the diagnosis of isolated SSPE (hospital-acquired VTE)	could confound study outcomes due to influence of co-treatments
13) Known pregnancy or breast-feeding	contraindication to rivaroxaban
14) Lack of safe contraception in women of childbearing potential	rivaroxaban is contraindicated in pregnancy
15) Refusal or inability to provide informed consent	unethical
16) Prior enrolment in this trial	confounds study outcomes

2

3 Abbreviations: DVT, deep vein thrombosis; VTE, venous thromboembolism

4

For peer review only - http://bmjopen.bmj.com/site/about/guidelines.xhtml

Table 3. Study Schedule

Study Period	Enrolment & Allocation	Baseline	Post-allocation		Close-out
**Visit/Follow-up phone call**	**1**		**2✆**††**	**3✆****	**4✆****
**Time (day with allowed visit window)**	**d0**	**d0**	**d10 (7-12)**	**d30 (28-35)**	**d90 (88-95)**
Eligibility screen (in-/exclusion criteria)	x				
Patient information and informed consent	x				
Bilateral whole-leg compression ultrasonography *	x				
Randomization	x				
Demographic characteristics		x			
Risk factors for venous thromboembolism		x			
Symptoms of venous thromboembolism		x			
Comorbid conditions		x			
Physical examination findings		x			
Laboratory test results		x			
Imaging findings		x			
Concomitant treatments		x			
Health-related quality of life (PEmb-QoL) †		x		x	x
Functional status ‡					x
Treatment setting		x			
Distribution and instruction of study drug		x			
Daily intake of study medication: placebo or rivaroxaban		x (immediate start on day 0)			
Instructions & distribution of patient diary §		x			
Recurrent venous thromboembolism			x	x	x
Clinically significant bleeding			x	x	x
All-cause mortality			x	x	x
Medical resource utilization			x	x	x
Time to symptom resolution			x	x	x
New concomitant treatments			x	x	x
Interruption of the study drug			x	x	x
Adherence ||					x
Serious adverse events reporting #		x (immediate start after inclusion on day 0)			

* Participants with concomitant deep vein thrombosis are excluded from further study participation (screening failures).

† At the baseline visit, participants are asked to fill in the PEmb-QoL questionnaire. For follow-up assessments, participants receive the PEmb-QoL including a pre-stamped envelope by mail and are asked to complete and return the questionnaire. If the follow-up interview is done in-person, the PEmb-QoL can be administered during the office visit.

For peer review only - http://bmjopen.bmj.com/site/about/guidelines.xhtml

For peer review only

‡ Functional status is assessed using the post-VTE functional status scale

§ The first part of the patient diary consists of an information on the study outline, surveillance interviews, and symptoms and signs suggestive for recurrent venous thromboembolism and bleeding, the investigator's contact information/emergency telephone number if these symptoms/signs occur, how to take the study medications, and instructions to return the drug bottles at the end of the study. The second part consists of a patient diary where patients are asked to record dates and type of outcomes and measures of health resource utilization (e.g., physician visits), and the time to symptom resolution and return to work/usual activities.

|| Adherence is assessed by counting the pill count of the returned medication bottles.

A serious adverse event is defined as any untoward medical occurrence that results in in death, is life-threatening, requires in-patient hospitalization or prolongation of existing hospitalization, results in persistent or significant disability/incapacity, or is a congenital anomaly/birth defect. In addition, important medical events that may jeopardize the patient or may require an intervention to prevent one of these outcomes is also considered serious.

** phone or in-person follow-up, depending on local practice

†† see Supplementary File 2 for the case report form of the 10-day follow-up interview as an illustration of the content of the follow-up phone calls

For peer review only

For peer review only - http://bmjopen.bmj.com/site/about/guidelines.xhtml

Figure. Overview of the Study Design

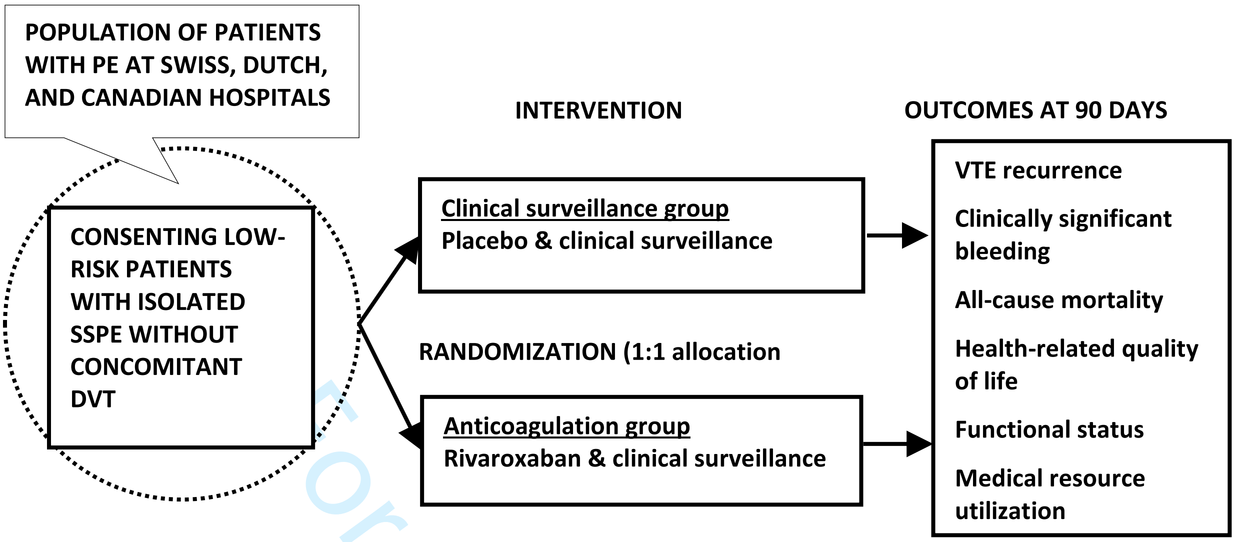

For peer review only

SUPPLEMENTARY FILES

Supplementary File 1: English language example of the informed consent form

For peer review only

SAFE-SSPE

Clinical surveillance vs. anticoagulation for low-risk patients with isolated subsegmental pulmonary embolism: a multicenter randomized placebo-controlled study

Original title of the study:
Clinical surveillance vs. anticoagulation for low-risk patients with isolated subsegmental pulmonary embolism: a multicenter randomized placebo-controlled non-inferiority trial (SAFE-SSPE)

This study is organized by:
Prof. Drahomir Aujesky, MD, MSc, Inselspital, Bern University Hospital

Dear Sir / Madam,

You have been diagnosed with a small blood clot in your pulmonary (lung) blood vessels, which specialists call a subsegmental pulmonary embolism. For this reason we would like to ask you if you would be willing to participate in a clinical study. This study is described below as follows: first a short summary to inform you about the study, followed by a more detailed description.

Summary

1	**Aim of the study**<br>The study explores the optimal management for adults with small blood clots in the pulmonary arteries, so called isolated subsegmental pulmonary embolism. It is currently unclear whether patients with isolated subsegmental pulmonary embolism really do benefit from the current treatment with blood thinners (anticoagulants). We are conducting this study to compare the effectiveness and safety of treating subsegmental pulmonary embolism with and without blood thinners.
2	**Selection of study participants**<br>You suffer from an isolated subsegmental pulmonary embolism and have a low-risk of complications. This is why we are providing you with this information leaflet.
3	**General Information about the study**<br>After exclusion of the simultaneous presence of a blood clot in the leg veins (deep vein thrombosis), patients with isolated subsegmental pulmonary embolism, will be 1:1 randomly divided with a probability of 50% each into one of two groups in this study: clinical surveillance plus placebo (a dummy-drug; a pill that does not contain any active medication) or clinical surveillance plus a blood thinner medication called rivaroxaban. Rivaroxaban is a tablet that acts as a blood thinner and is approved in Switzerland and other countries for the treatment of pulmonary embolism. The placebo/rivaroxaban group allocation is blinded, i.e neither you nor your physician nor the research team will know whether you are receiving placebo or rivaroxaban (double-blind). In an emergency however, this information can be made available at any time. The study will randomize 276 participants over a period of 4 years.<br>The treatment during the study will last 90 days. During this period the two treatment groups will be clinically monitored and compared in terms of risk of blood clots (thrombosis / embolism), bleeding, mortality, quality of life and the use of medical resources. This information will be gathered by means of three telephone calls at day 10, 30 and 90 after study enrollment.

For peer review only - http://bmjopen.bmj.com/site/about/guidelines.xhtml

4	**Procedure**<br>The first study visit will take place on-site immediately after enrollment and signing of the informed consent form (total duration approx. 1.5 hours) and will include the following:<br>- Ultrasound examination of the leg veins to exclude a blood clot (thrombosis). Patients with leg vein thrombosis (deep vein thrombosis) cannot participate in the study.<br>- Study participants who do not have deep vein thrombosis will be randomly assigned to either the group with clinical surveillance plus placebo or to the group with clinical surveillance plus blood thinner medication with rivaroxaban.<br>- Collection of baseline data: questions on symptoms, pre-existing medical conditions, medication; compilation of examination findings, laboratory results; questionnaire on quality of life (1)<br>- Provision of a patient diary containing study information and to record any symptoms and/or doctor's visits/ hospital stays.<br>- Provision of the corresponding study medication and start of treatment.<br><br>For the 90-day study phase, the study medication should be taken daily as prescribed. Further study contacts will be made by telephone:<br>- Telephone call 1: after approx. 10 days (duration approx. 10 minutes)<br>- Telephone call 2: after approx. 30 days (duration approx. 10 minutes)<br>- Questionnaire on quality of life (2): by mail or in-person after approx. 30 days (duration approx. 10-15 minutes)<br>- Telephone call 3: after approx. 90 days (duration approx. 10 minutes)<br>- Questionnaire on quality of life (3): by mail or in-person after approx. 90 days (duration approx. 10 minutes)<br>During the telephone calls you will be asked about possible recurrent embolism/ thrombosis and about bleeding as well as doctor's visits / hospitalizations, return to work or usual activities, quality of life and functional status, medications, and symptoms.
5	**Benefit**<br>We cannot guarantee you any personal benefit from participating in the study. Even if participation in the study does not directly benefit you, we do expect that the information we gain from the study will enable us to optimize the management for people with subsegmental pulmonary embolism in the future.
6	**Rights**<br>You decide voluntarily whether you would like to participate in the study or not. Your decision does not affect your medical treatment and you do not have to justify it.
7	**Responsibilities**<br>If you decide to participate in the study, we ask you to comply with certain requirements (e.g participation in the first study visit which includes an ultrasound examination of your leg veins, participation in telephone interviews).
8	**Risks**<br>A treatment strategy without blood thinning medication (placebo) for subsegmental pulmonary embolism could potentially lead to an increased risk of recurrent embolism or thrombosis. However, observational studies have shown that low-risk patients like yourself without blood thinners do not have an increased risk of thrombosis or embolism when compared to patients treated with blood thinners (anticoagulation). A treatment strategy with blood thinners increases the risk of bleeding.

For peer review only - http://bmjopen.bmj.com/site/about/guidelines.xhtml

9	**Other treatment options**<br>Your physician will advise you on which other treatment options are available if you do not wish to participate in this study.
10	**Study results**<br>If we detect any findings that may affect your health during the study, we will inform you about these findings.
11	**Data confidentiality**<br>We comply with all legal data protection regulations, and all parties involved are bound by confidentiality. Your contact details will be forwarded to Inselspital, Bern University Hospital so that we can contact you for further follow-up during the study (see section 4. Procedure). The CT images of the lung may be sent unencrypted (i.e. with information about your name and date of birth) to the radiologists at the Bern University Hospital in Switzerland (coordinating study center) for subsequent central review. The rest of your personal and medical data will be protected and only used in an encrypted format. The encrypted data will only be used for other research projects if you give your separate consent for this.
12	**Withdrawal**<br>You can choose to no longer participate and withdraw from the study at any time. The data collected up until the point of study withdrawal will still be analyzed.
13	**Compensation**<br>You will not receive any financial compensation.
14	**Liability**<br>The insurance company Zürich Versicherungs-Gesellschaft AG will cover any damages that may arise within the scope of this study.
15	**Funding**<br>The study is financed by the Swiss National Science Foundation (SNSF). Bayer AG supports the study by providing the study medications (rivaroxaban and placebo).
16	**Contact person:**<br>Principal Investigator<br>Prof. Dr. med. Drahomir Aujesky<br>Universitätsklinik für Allgemeine Innere Medizin<br>Inselspital, Universitätsspital Bern<br>3010 Bern<br>The study team of Prof. Aujesky is available 24h a day at 031 632 77 83 (during office hours) or 079 737 26 55 (in emergencies outside of normal office hours).

For peer review only

For peer review only - http://bmjopen.bmj.com/site/about/guidelines.xhtml

Detailed information

1. Aim of the study

The aim of this study is to investigate the efficacy and safety of a management strategy with and without blood thinning medication for patients with small blood clots in their pulmonary arteries, so-called isolated subsegmental pulmonary embolism.

2. Selection of study participants

All persons aged 18 years and over who suffer from an isolated subsegmental pulmonary embolism, who do not have a simultaneous blood clot in the deep veins of the leg (deep vein thrombosis), and who are at low risk of complications can participate in this study.

However, the following individuals cannot participate in the study: those with simultaneous deep vein thrombosis, previous unprovoked venous thrombosis or pulmonary embolism (venous thromboembolism) i.e. a venous thromboembolism which occurred without a specific trigger, active cancer, unstable cardiovascular or respiratory function, active bleeding or those who have a very high bleeding risk, persons who take medications that interact with rivaroxaban, those with severe kidney or liver disease, an allergy towards rivaroxaban, pregnant or breastfeeding women, as well as persons who have an indication for a strong (therapeutic dose) blood thinning medication because of another concomitant disease, persons who already received therapeutic-dose blood thinning medication for >72 hours before study enrollment, or who were hospitalized for more than 72 hours before the diagnosis of a subsegmental pulmonary embolism was made.

3. General Information

Pulmonary emboli are blood clots in the pulmonary arteries and cover a broad spectrum ranging from large to very small (so-called subsegmental) emboli. The vast majority of pulmonary emboli, regardless of their size, are treated with blood thinners which are associated with a risk of bleeding. However, the risk-benefit ratio of blood thinners in treating subsegmental pulmonary embolism is unclear.

In this international study with study centers in Switzerland, the Netherlands and Canada, the effectiveness and safety of a treatment strategy with or without blood thinning medication for patients with subsegmental pulmonary embolism will therefore be compared. Patients with isolated subsegmental pulmonary embolism but without simultaneous deep vein thrombosis will, after appropriate explanation and informed consent, be randomized 1:1 with a probability of 50% each into one of two groups: clinical surveillance plus placebo or clinical surveillance plus a blood thinning medication called rivaroxaban. The group allocation is blinded, i.e. neither the patient nor the physician (including the study team) are aware of the group allocation (double-blind principle). Patients who have already agreed to participate in the study but who are later diagnosed with a deep vein thrombosis, cannot continue to participate in the study. The data collected up until the diagnosis of the deep vein thrombosis will be kept confidential and stored in our encrypted database.

The study medication rivaroxaban is a blood thinner in tablet form, which is approved for use in Switzerland, the EU, and North America for the treatment of pulmonary embolism. During the first 21 days, rivaroxaban will be taken at a dose of 15mg twice daily; from day 22 the daily dose will be changed to a 20mg tablet once daily. For participants randomized to the placebo group the same treatment regimen applies (i.e. 2 tablets per day for the first 21 days, followed by 1 tablet per day thereafter). The total duration of treatment during the study is 90 days. During this study period, both treatment groups will be interviewed three times via telephone calls on days 10, 30 and 90 after study enrollment in order to carry out clinical surveillance and at the end of the study to compare the two treatment groups with regards to the risk of thrombosis/emboli, bleeding, mortality, quality of life (PEmb-QoL questionnaire), functional status, as well as the use of medical resources.

For peer review only - http://bmjopen.bmj.com/site/about/guidelines.xhtml

Large pulmonary emboli are usually treated with blood thinners for at least 3 months in order to reduce the risk of thrombosis / pulmonary embolism recurrence. As a side effect, however, the blood thinning medication increases the risk of bleeding, and 1-5% of patients within 3 months of starting therapy experience severe bleeding, e.g. gastrointestinal bleeding, or less frequently a brain hemorrhage. For persons with subsegmental pulmonary embolism and a low risk of complications there is no standard treatment currently available, since it is not clear from today's scientific knowledge if treatment with or without a blood thinner is better. Certain physicians prescribe blood thinners in these cases, whilst other physicians avoid using them. If our study shows that treatment with blood thinners is no better at preventing venous thrombosis / pulmonary embolism compared to a management strategy without blood thinners, then unnecessary treatment with blood thinners and the associated bleeding risk could be reduced in low-risk patients with isolated subsegmental pulmonary embolism.
The study will randomize 276 patients over 4 years.

We are conducting this study in accordance with Swiss laws and regulations. We also comply with all internationally recognized guidelines. The responsible ethics commission and EudraCT have reviewed and approved the study.
A description of this study can also be found on the website of the Bundesamt für Gesundheit (Federal Office of Public Health): www.kofam.ch (registration number SNCTP000003905).

4. Procedure

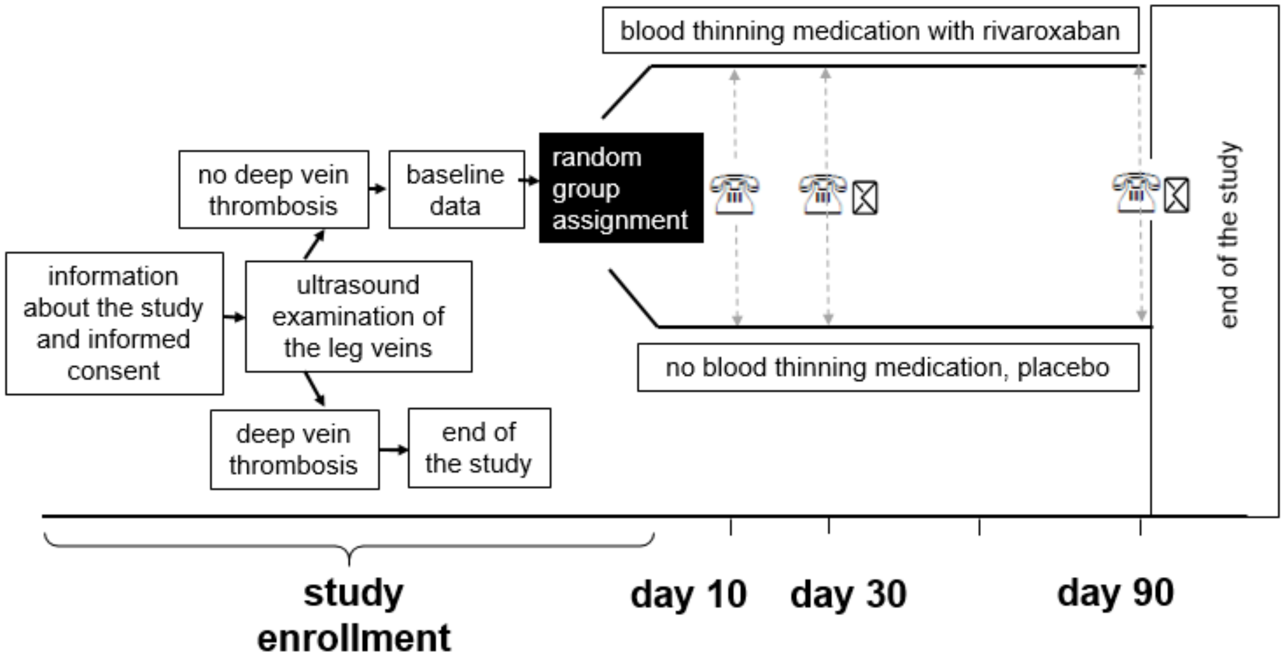

After you have been informed about the study and received this study information document, you will have time to ask any questions you may have about the study, the study information, or the informed consent form. If you wish to participate in the study, you must confirm this in writing by signing the consent form. You can keep this study information and you will receive a copy of the signed consent form.

In women of childbearing age, a pregnancy test (urine or blood test) will be performed prior to inclusion in the study, if a test has not already been performed during routine clinical practice.

For peer review only - http://bmjopen.bmj.com/site/about/guidelines.xhtml

At the time of study enrollment a study visit will take place on-site immediately after signing the consent form. This study visit will include the following:

Collection of contact details: study personnel will ask you about your contact information (so that we can contact you for further monitoring during the study), contact details of family members/next of kin and of your general practitioner.

Ultrasound examination of the leg veins: a systematic and thorough ultrasound examination of the veins in both legs will be carried out to rule out a deep vein thrombosis, i.e. a blood clot causing a blockage in a vein. The technical term for the ultrasound procedure used is called compression ultrasonography. The veins in the thigh and lower leg will be examined for their compressibility. If a thrombosis is present, the corresponding section of vein is barely or not at all compressible. This is a non-invasive and painless procedure without any side effects. This examination will be performed on-site and with the patient lying down (if necessary, parts of the examination may also be performed in the seated position) and will be performed by a physician experienced in ultrasound examinations; the ultrasound examination takes approx. 15-20 minutes per leg, i.e. a total of about 30-40 minutes. This examination is performed because the presence of a deep vein thrombosis is an exclusion criterion for the present study.
For patients who are diagnosed with a deep vein thrombosis during the leg ultrasound examination, the study ends here and the further management plan will be determined by the treating physician.

Data collection at baseline: if a deep vein thrombosis has been excluded, study personnel will ask you questions about demographic data, risk factors for venous thromboembolism (i.e. pulmonary embolism or thrombosis), possible symptoms of venous thromboembolism, other diseases, and the medication you are taking. We will collect information from the treating physician's records on your previous illnesses, examination findings, laboratory results and results from the computer tomography examination (CT scan) through which the subsegmental pulmonary embolism was originally diagnosed. We will also ask you to complete a quality of life questionnaire regarding your diagnosis of subsegmental pulmonary embolism. Completing the questionnaire will take about 10-15 minutes. The collection of baseline data will be carried out on-site and takes approx. 30 minutes.

Randomization and delivery of the study medication and patient diary: We will provide you with the study medication, selected for you at random (randomization), i.e. rivaroxaban or placebo; you should start taking the medication immediately. In addition, we will also provide you with a patient diary which contains information about the study, the study medication and contact details of the Principal Investigator and his study team. Furthermore, we ask you to record in the diary the date of symptom resolution and the date of return to work or your usual housekeeping/leisure activities. You should also note in your diary if you suffer any bleeding or thrombosis / pulmonary embolism recurrence, if you need to visit a physician (general practitioner, specialist or emergency department) or if you are hospitalized.

The remaining study contacts will take place via telephone call and no further study visits are necessary. The remaining study contacts will take place as follows:

Telephone call: approx. 10 days (7-12 days) after study enrollment.

Telephone call: approx. 30 days (28-35 days) after study enrollment.

Questionnaire on quality of life (2): approx. 30 days (28-35 days) after study enrollment, by mail or in-person.

Telephone call: approx. 90 days (88-95 days) after study enrollment.

For peer review only - http://bmjopen.bmj.com/site/about/guidelines.xhtml

Questionnaire on quality of life (3): approx. 90 days (88-95 days) after study enrollment, by mail or in-person.

During the follow-up phone calls or visits you will be personally interviewed by study personnel from the Inselspital, Bern University Hospital (the institution responsible for the study in Switzerland). In case we cannot reach you personally after three attempts, we will contact your general practitioner/treating physician or other health care professional involved in your care (e.g. health care personnel from a retirement home, nursing home, or rehabilitation center) and/or the contact person you identified as your next of kin (relative, other close person). We expect the telephone interviews to take about 10 minutes each. During these telephone calls, the study coordinator will ask you about any new thrombosis / embolism and bleeding. You will also be asked about any doctor's visits or any hospital stays, the date of return to work or your usual daily activities, your functional status in everyday life, your medications and about any possible symptoms or signs compatible with thrombosis or embolism, or bleeding.
Approx. one and three months after study enrollment, we will send you another quality of life questionnaire by mail and ask you to complete it and return it to us in a pre-paid envelope.

Returning the study medication and patient diary: Once treatment is complete at the end of the study, we will ask you to please return the pill bottles containing the remaining tablets, as well as the patient diary to us in a pre-paid envelope.

Your general practitioner will be informed at the beginning of the study about your participation. It is also possible that we may contact your general practitioner / treating physician or another health care professional (e.g. hospital doctors, health care professionals from the emergency department, rehabilitation, nursing home, specialist doctors) to collect healthcare data regarding your hospital stays and any newly diagnosed illnesses. This is in order to obtain accurate medical information about your health and about the effects or side effects of treatment.

We may have to prematurely exclude you from the study. This may happen if you end up needing a long-term blood thinner for a reason other than for treatment of your subsegmental pulmonary embolism, if you end up needing a long-term medication that interacts with the study medication, if you develop recurrent pulmonary embolism or thrombosis, if you experience severe bleeding, or if you become pregnant. In this case we will inform you about the end of your study participation, compile the information collected during the previous telephone calls (see above), and ask you to please return all remaining medication (rivaroxaban or placebo) that we have given you.

5. Benefit

We cannot guarantee you any personal benefit from participating in the study. Even if participation in the study does not directly help you, we expect the information we gain from the study will contribute to improving the management of patients with subsegmental pulmonary embolism in the future. However, it is possible that by participating in the study and having the associated follow-up telephone calls, any complications you may have can be identified more quickly and communicated to your treating physician.

6. Rights

Your participation is voluntary. If you do not want to participate or later choose to withdraw from the study, tyou do not have to justify this if you do not want to. Your medical treatment/care is guaranteed regardless of your decision. You may ask questions about participation in the study at any time. Please contact the person named at the end of this information sheet.

For peer review only - http://bmjopen.bmj.com/site/about/guidelines.xhtml

7. Responsibilities

As a participant, it is necessary that you

- adhere to the necessary guidelines and requirements of the study, such as participation in the first study visit including ultrasound examination of the leg veins, daily intake of the study medication as prescribed, participation in telephone follow-up calls (or in-person visits), keeping a patient diary, completing and returning the questionnaires on quality of life as mentioned above, and returning the pill bottles at the end of the study.
- inform the study team about new symptoms, new ailments and changes in your condition that may be related to a new thrombosis or embolism (e.g. new or increasing shortness of breath, new or worsening cough, new chest pain, coughing up blood, one-sided pain or swelling in your leg), as well as in the event of prolonged or severe bleeding or signs of possible bleeding (unusual weakness, tiredness, paleness, dizziness, headache, unexplained swelling or chest pain).
- inform the study team about simultaneous treatment and therapy from another physician and about the medication that you take.

8. Risks and burden for the participants

A treatment strategy without blood thinners for subsegmental pulmonary embolism could potentially lead to an increased risk of recurrence of pulmonary embolism or deep vein thrombosis. However, observational studies have shown that in low-risk patients like yourself there was no increased risk of recurrent pulmonary embolism or thrombosis in people without blood thinning treatment compared to those who took anticoagulants. A treatment strategy with a blood thinner leads to an increased risk of bleeding.

For women who may become pregnant

Based on animal studies, we know that rivaroxaban does not cause harm to unborn animals. Nevertheless, the effects of the study medication on the unborn child have not yet been adequately investigated. We do however know from animal studies that rivaroxaban during pregnancy is harmful and can lead to maternal bleeding complications. For this reason, female study participants must use a reliable contraceptive method during the study (e.g. hormonal methods such as the pill or coil / intrauterine system).

Should you become pregnant during the study, you must inform the study team immediately and stop taking the study medication. The study physician will discuss the next steps with you. If you are breastfeeding, you are excluded from participation.

9. Other treatment options

You do not have to participate in this study. If you do not participate, your treating physician will work with you to decide whether to start treatment with a blood thinner for at least 3 months (depending on the blood thinner, additional blood thinning injections may be necessary during the first few days), or whether to choose a management strategy without a blood thinning medication.

10. Results from the study

During the trial, the study physician will inform you about any new findings that may affect the benefit of the trial or your safety and thus your consent to participate in the trial. You will receive the information orally and in writing.

11. Confidentiality of data

Your personal and medical data will be collected for this study. Very few professionals will see your unencrypted data, and this will only occur for the purpose of carrying out tasks related to the study. Data collected for study purposes will be encrypted. Encryption means that all data related to you that could identify you (name, date of birth) is deleted and replaced by a number-key. The numbered-key list and your non-encrypted contact details can be accessed by the study team at

For peer review only - http://bmjopen.bmj.com/site/about/guidelines.xhtml

For peer review only

Inselspital, Bern University Hospital (institution responsible for conducting the study in the Switzerland), so that you and, if necessary, the contact person you identified or your treating physician/medical staff can be contacted for further follow-up during the study (e.g. telephone calls, questionnaires etc., see section 4. Procedure).
All other professionals from Switzerland, the Netherlands, and Canada who are involved in our study only have access to your encrypted data for research purposes (analysis). Those who do not know the number-key cannot therefore trace the data back to you. In the case of a publication, the aggregate data also cannot be traced back to an individual person such as yourself. Your name will never appear on the internet or in a publication. Sometimes for publication in a journal there is a requirement to pass on the individual data (so-called raw data). If individual data must be transmitted, then the data is always encrypted and cannot be traced back individually to you. All persons who have access to your data within the scope of the study are bound by the principles of confidentiality. The requirements for data protection will be observed and you as a study participant have the right to view your data at any time.

Data is collected electronically using a database for research purposes (EDC System secuTrial at the Clinical Trial Unit, Mittelstrasse 43, 3012 Bern, Bern University Hospital). Data will be stored for at least 10 years.

It is possible that your encrypted data may be reused for other investigations at a later date or sent to another database in Switzerland or abroad for as yet undefined further use. This other database must comply with the same standards as the database for this study. For this further use of your data, we ask you to sign an additional consent form at the very end of this document.

This study may be reviewed by the responsible ethics committee, the medication authority Swissmedic or by the institution that initiated the study (Bern University Hospital). The Principal Investigator may have to disclose your personal and medical data for such checks. It may also be the case that a representative of the insurance company might also inspect your data in exceptional cases in the event of damage. Bayer AG who provides the study medication, may receive access to your encrypted data (i.e. without any identifying information such as name or date of birth) for monitoring purposes. All persons must maintain absolute confidentiality.

As mentioned above, it is possible that the contact person you identified, your general practitioner/treating physician or other healthcare professionals that treat you (e.g. in retirement homes, nursing homes, rehabilitation centers) may be contacted to provide us with information about your state of health.

Subsequent analyses of the CT images by radiologists at Bern University Hospital

In order to guarantee a uniform diagnosis of an isolated subsegmental pulmonary embolism among all study participants, the CT (computer tomography) images of the lung will be forwarded unencrypted i.e. with details of your name and date of birth, to the radiologists at Bern University Hospital for central review:

Inselspital Bern
University Institute for Diagnostic, Interventional, and Pediatric Radiology (Universitätsinstitut für Diagnostische, Interventionelle und Pädiatrische Radiologie)
Freiburgstrasse 10
CH-3010 Bern

CT images will be stored in a digital radiological images archiving system for at least 10 years.

For peer review only - http://bmjopen.bmj.com/site/about/guidelines.xhtml

12. Withdrawal

You can stop and withdraw from the study at any time if you wish. The data collected up to that point will still be evaluated in an encrypted format, otherwise the entire project will lose its value. It is not possible to completely anonymize your data in case of withdrawal, i.e. the data will remain encrypted (coded), i.e. without any patient-identifiable information. Please check whether you agree to this before you participate in the study.

13. Compensation for participants

If you participate in this study, you will not receive any financial compensation.
You or your health insurance company will not incur any costs as a result of study participation.

14. Liability

The institution (Inselspital, Bern University Hospital), which is responsible for carrying out the study in Switzerland, is liable for any damage that you might incur in connection with the research activities (e.g. investigations/treatment). The conditions and procedure for this is regulated by law. The Inselspital, Bern University Hospital has therefore taken out insurance with Zürich Versicherungs-Gesellschaft AG to cover liability in the event of possible damages.
The same liability rules apply as would to a treatment outside the scope of the present study. Namely, in the event of damages attributable to rivaroxaban (an approved medical substance used in accordance with medical standards), or damages which occur as part of the use of a placebo, or damages which would also have occurred in the case of usual care, the same liability rules apply as for a treatment outside of the study.
If you suffer any damages, please contact your study physician or the insurance company mentioned above.

15. Financing of the study

The study is fully funded by the Swiss National Science Foundation (SNSF). Bayer AG supports the study by providing the study medication.

16. Contact person

If you have any questions, uncertainties or emergencies that arise during or after the study, you can contact the Principal Investigator at any time:

Principal Investigator:
Prof. Dr. med. Drahomir Aujesky
Universitätsklinik für Allgemeine Innere Medizin
Inselspital, Universitätsspital Bern
Freiburgstrasse
3010 Bern

The study team of Prof. Aujesky can be reached 24h a day at: 031 632 77 81 or
079 737 26 55 (in emergencies outside office hours)
Email: safe-sspe@insel.ch

17. Glossary (technical terms explained)

- What does “placebo” mean?
  Some people who receive a medication do not actually get better from the medication itself, but rather from the care and attention of the physician. This can be seen by the fact that some people can feel better, even if they get a so-called sham medication or dummy-drug. This sham medication looks like a real medication and is also packaged identically. However, there is in fact no active ingredient in this sham medication. It is called a "placebo".
  Sometimes one treats some of the participants in a clinical trial with the real medication (which contains the active ingredient) and the other participants with a placebo (without the active

For peer review only - http://bmjopen.bmj.com/site/about/guidelines.xhtml

ingredient). It is then easier to make a comparison and assess how well the medication actually works or whether the improvement observed only occurred because people received care and attention. Sometimes the improvement simply corresponds with the natural course of the disease.

- What does “randomized” mean?
  Many studies compare two or more different types of treatment. For example, one can compare an active “real” medication with a placebo. One then forms two groups of participants, one group receives the real medication and the other group the placebo. "Randomization" then means that participants are randomly assigned to different groups. Therefore, it is a coincidence whether the participant receives the real medication or the placebo.

- What does "double-blind" mean?
  To “blind” a study serves to obtain better and more accurate results.
  "Double-blind" is therefore a study where neither the participants nor the researchers know whether a study participant is receiving the real medication or the placebo. Only the independent person who assigned the group allocation knows who receives what. When the test is over, the "blinding" will be removed. In an emergency, the "blinding" can also be removed earlier.
  A person who knows that he or she is receiving the real medication and not the placebo, will pay very different attention to symptoms in his/her body than someone who knows that he/she is only receiving the placebo. This can lead people who receive the real medication to overestimate the effect of the medication compared to those who only receive the placebo.

For peer review only

For peer review only - http://bmjopen.bmj.com/site/about/guidelines.xhtml

Informed consent

Written declaration of consent for participation in a study project
Please read this form carefully. Please ask if you do not understand something or would like to know more. Your written consent is required for participation.

**IRB-Number (after submission):**	2019-02297
**Title of the study (scientific and layperson language)**:	Clinical surveillance or blood thinner for low-risk patients with isolated subsegmental pulmonary embolism: a multicenter randomized placebo-controlled study
**Responsible institution (Sponsor with address)**:	Inselspital, Universitätsspital Bern<br>Prof. Dr. med. Drahomir Aujesky<br>Freiburgstrasse<br>3010 Bern
**Site where study will be conducted**:	Inselspital, Universitätsspital Bern<br>Universitätsklinik für Notfallmedizin<br>Freiburgstrasse<br>3010 Bern
**Responsible investigator at study site:**<br>Surname und first name in block capital letters:	Prof. Dr. med. Drahomir Aujesky
**Study participant:**<br>Surname and first name in block capital letters:<br>Date of birth:	☐ female ☐ male

- I have been informed, both verbally and in writing, by the study team member signing below about the purpose of the study, the course of the study with clinical surveillance and rivaroxaban (blood thinner) or placebo, and about possible advantages and disadvantages as well as possible risks.
- I am voluntarily participating in this study and acknowledge the content of the written patient information provided. I have been given enough time to make my decision.
- My questions regarding participation in this study have been answered. I will keep the written patient information provided and will receive a copy of my written informed consent form.
- I have been informed about possible other treatments and treatment procedures.
- I agree to my general practitioner being informed about my participation in the study.
- I agree to allow the sponsor-representative's medical experts, the responsible ethics committee and the medication authority Swissmedic to inspect my unencrypted data for testing and control purposes, but in keeping with strict confidentiality regulations.
- I will be informed in the event of study findings that directly affect my health.
- I know that my personal contact details will available to the study team at Inselspital, Bern University Hospital (responsible study center in the Switzerland), so that the study team can contact me and if necessary the contact person I identified, or contact my treating physician/medical staff for further follow-up during the study period. Furthermore, I also authorize my treating physician(s) or the healthcare professionals from other institutions that treat me (e.g. heath care professionals from retirement homes, nursing homes, rehabilitation centers) to

For peer review only - http://bmjopen.bmj.com/site/about/guidelines.xhtml

provide the study team at Inselspital with any of my health-related data that may be relevant to this study. This information will be used exclusively for study purposes only.

- I know that my health-related and personal data can only be passed on in an encrypted format for research purposes for this study (including transfer abroad).
- I agree to the CT (computer tomography) images of my lungs, which were used to diagnose my subsegmental pulmonary embolism, being forwarded unencrypted to the radiologists at Bern University Hospital for central review (i.e. with name and date of birth specified).
- If during the ultrasound examination of my leg veins (which is performed on all study participants during the initial study visit), I am diagnosed with a deep vein thrombosis, I acknowledge that I cannot continue to participate in the study. The data collected up until this point will be kept confidential and stored in an encrypted form.
- I can withdraw from the study at any time and without giving a reason. My further medical treatment is always guaranteed and independent of whether I participate in the study or not. The data and samples collected up until the point of withdrawal will be used for the analyses in the study.
- I have been informed that an insurance company will cover damages that are attributable to this study.
- I am aware that the responsibilities stated in this study participant information sheet must be complied with. In the interest of my own health, the Principal Investigator may exclude me from the study at any time.

Place, date	Signature of participant

Confirmation by the local investigator: I hereby confirm that I have explained the nature, significance and scope of the study to this participant. I confirm that I will fulfil all my obligations regarding this study in accordance with the relevant laws. If at any time during the study, I become aware of any aspect that may affect the participant's willingness to participate in the study, I will inform the participant immediately.

Place, date	Surname and first name of the local investigator in block capital letters<br><br>Signature of the local investigator

For peer review only - http://bmjopen.bmj.com/site/about/guidelines.xhtml

Declaration of consent to further use of data from this study in an encrypted form

**Participant:**<br>Surname and first name in block capital letters:<br>Date of birth:	☐ female ☐ male

I allow my data from this study to be re-used for medical research. This means that the data may be stored in a database and used for future, as yet undefined, research projects for an unlimited period of time. This consent is indefinite.

My decision is voluntary and I can revoke this decision at any time. If I withdraw from the study, my data will be anonymized. In the event of my decision to withdraw, I will inform the investigator and do not have to justify my decision.

I understand that the data is encrypted and the security key to decode it will be kept safe and secure. The data can be sent abroad or inland to other databases for analysis, if the same standards as in Switzerland are complied with. All legal requirements regarding data protection are complied with.

Normally, all data will be analyzed in its entirety and the results published in a summary form. If a result that is important to my health should arise, then it is possible that I will be contacted by a member of the study team.

If results from the data are commercialized, I have no claim to a share of the commercial profits.

Place, date	Signature of the participant

Confirmation by the local investigator: I hereby confirm that I have explained to this participant the nature, significance and implications of the further use of his/her data.

Place, date	Surname and first name of the informing local investigator in block capital letters<br><br>Signature of the local investigator

For peer review only - http://bmjopen.bmj.com/site/about/guidelines.xhtml

Supplementary File 2: Case Report Form (CRF) for follow-up interview 10 days after randomization

For peer review only

Patient ID

Follow up interview

(04.05.2020 - 13:25:25 (CEST))

Follow-up interview number ○ 1 ○ 2 ○ 3

Date of follow-up interview phone call [ ] dd.mm.yyyy

Follow up interview completed ○ yes ○ no

Please indicate the reason why follow-up call wasn't completed ○ lost to follow-up ○ withdrawal from study ○ patient died

Please specify the reason for withdrawal [ ]

Contact(s) made to obtain follow-up information

- **Patient** ☐
- **Designated contact person (family or friend)** ☐
- **Primary care physician** ☐
- **Other** ☐
- **Please specify "other"** [ ]

Agreement for consecutive follow up
○ patient agrees to be contacted for final assessment at 90 days
○ patient agrees for passive follow-up (i.e. information collected from hospital medical records and/or primary care physician)

Planned date of next follow-up call [ ] dd.mm.yyyy

Follow up interview

Patient ID

Medical outcomes

(04.05.2020 - 13:25:25 (CEST))

VTE RECURRENCE

VTE recurrence ○ Yes ○ No

Interruption of study medication and unblinding is indicated if VTE is objectively confirmed

Date of diagnosis [ ] dd.mm.yyyy

Confirmatory diagnostic exam:

- **CTPA** ☐
- **ventilation-perfusion scintigraphy** ☐
- **pulmonary angiography** ☐
- **venous ultrasound** ☐
- **IV phlebography** ☐
- **CT or MRI for thrombosis of the iliac vein or vena cava inferior** ☐
- **echocardiography** ☐

Type of recurrent VTE ○ PE ○ DVT

Localization of PE
○ subsegmental PE
○ more proximal PE

Localization of DVT
○ proximal DVT (V.cava, iliac vein, common or superfician femoral vein, popliteal vein)
○ distal DVT (posterior or anterior tibial vein, peroneal vein, gastrocnemial vein, soleal vein)

Stop of study medication following recurrent VTE ○ Yes ○ No

Enter the date of stopping the study medication [ ] dd.mm.yyyy

CLINICALLY SIGNIFICANT BLEEDING

Clinically significant bleeding ○ Yes ○ No

Clinically significant bleeding 1

Date of bleeding episode [ ] dd.mm.yyyy

Type of physician contact(s)

- **phone call to physician** ☐
- **office visit** ☐
- **ED visit** ☐
- **hospitalization** ☐
- **none** ☐

Treatment with antiplatelet agent or NSAID at the time of bleeding ○ Yes ○ No

- **Aspirin** ☐
- **clopidogrel** ☐

For peer review only - http://bmjopen.bmj.com/site/about/guidelines.xhtml

Patient ID

prasugrel	☐
ticagrelor	☐
NSAIDs	☐
Packed red cell transfusions	○ Yes ○ No
Indicate the number of units	
Decrease in hemoglobin of at least 20 g/L within 7 days after the bleeding event	○ Yes ○ No ○ Unknown
Site of bleeding	
epistaxis	☐
cutaneous	☐
subcutaneous	☐
intramuscular with compartment syndrome	☐
intramuscular without compartment syndrome	☐
gastrointestinal [melena, hematochezia, hematemesis]	☐
intracranial	☐
retroperitoneal	☐
pulmonary	☐
intraarticular	☐
intraspinal	☐
intraocular	☐
pericardial	☐
hematuria	☐
vaginal	☐
other	☐
other, please specify	
Diagnostic test(s) performed to localize bleeding source	
MRI	☐
CT	☐
ultrasound	☐
upper endoscopy	☐
lower endoscopy	☐
capsule endoscopy	☐
bronchoscopy	☐

For peer review only - http://bmjopen.bmj.com/site/about/guidelines.xhtml

Patient ID

cystoscopy	☐
arteriography	☐
erythrocyte scintigraphy	☐
diagnostic surgery	☐
other	☐
other, please specify	
**Treatment(s) of bleeding:**	
fresh frozen plasma	☐
recombinant factor VIIa concentrate	☐
prothrombin complex [factor II, VII, IX, X]	☐
factor Xa inhibitor-specific antidote [andexanet alpha]	☐
tranexamic acid	☐
invasive or surgical hemostasis procedures	☐
other	☐
other, please specify	
Interruption of study medication following bleeding	○ Yes ○ No
duration of interruption	○ definite ○ temporary
date of interruption	dd.mm.yyyy
date of restart	dd.mm.yyyy
**Clinical impact of overt bleeding:**	
temporary or permanent cessation of the study drug	○ Yes ○ No
overt bleeding associated with relevant pain	○ Yes ○ No
impairment of activities of daily life	○ Yes ○ No

More

For peer review only - http://bmjopen.bmj.com/site/about/guidelines.xhtml

Patient ID

Medical resource utilization

(04.05.2020 - 13:25:25 (CEST))

Initial hospitalization ongoing ○ Yes ○ No

Date of discharge from initial hospitalization [ ] dd.mm.yyyy hh:mm

Subsequent hospitalizations ○ Yes ○ No

Subsequent hospitalizations 1

Admission date [ ] dd.mm.yyyy

Discharge date [ ] dd.mm.yyyy

Name of hospital [ ]

Symptoms or signs leading to hospitalization

- **dyspnea** ☐
- **cough** ☐
- **chest pain** ☐
- **bloody cough [hemoptysis]** ☐
- **syncope** ☐
- **pleural effusion** ☐
- **leg pain** ☐
- **leg swelling** ☐
- **bleeding** ☐
- **other** ☐
  - **please specify** [ ]

Primary discharge diagnosis [ ]

Please specify [ ]

Discharge location [ ]

Other, please specify [ ]

Notes [ ]

More

Subsequent emergency department visits ○ Yes ○ No

Subsequent emergency department visits 1

Presentation date [ ] dd.mm.yyyy

Specify hospital name [ ]

For peer review only - http://bmjopen.bmj.com/site/about/guidelines.xhtml

Patient ID

Symptoms or signs leading to ED visit

- dyspnea ☐
- cough ☐
- chest pain ☐
- bloody cough [hemoptysis] ☐
- syncope ☐
- pleural effusion ☐
- leg pain ☐
- leg swelling ☐
- bleeding ☐
- other ☐
  - please specify

Primary diagnosis at the emergency department

Please specify

Notes

More

Subsequent physician outpatient visits ○ Yes ○ No

Subsequent physician outpatient visits 1

Visit date dd.mm.yyyy

Reason for visit

- dyspnea ☐
- cough ☐
- chest pain ☐
- bloody cough [hemoptysis] ☐
- pleural effusion ☐
- leg pain ☐
- leg swelling ☐
- bleeding ☐
- other ☐

Please specify

For peer review only - http://bmjopen.bmj.com/site/about/guidelines.xhtml

Patient ID

Physician diagnosis

Notes

More

Medical Resource Utilization

For peer review only

Patient ID

Time to symptom resolution

(04.05.2020 - 13:25:25 (CEST))

**Resolution of dyspnea**	○ Yes ○ No ○ Symptom has not been present
**Date of symptom resolution**	dd.mm.yyyy
**Resolution of cough**	○ Yes ○ No ○ Symptom has not been present
**Date of symptom resolution**	dd.mm.yyyy
**Resolution of chest pain**	○ Yes ○ No ○ Symptom has not been present
**Date of symptom resolution**	dd.mm.yyyy
**Resolution of bloody cough**	○ Yes ○ No ○ Symptom has not been present
**Date of symptom resolution**	dd.mm.yyyy
**Returned to work or usual activities**	○ Yes ○ No
**date of returning to work or usual activities**	dd.mm.yyyy

For peer review only - http://bmjopen.bmj.com/site/about/guidelines.xhtml

Patient ID

Discontinuation or modification of study medication

(04.05.2020 - 13:25:25 (CEST))

Was another anticoagulant agent started during study period? ○ Yes ○ No

Type of anticoagulant [ ]

Please specify [ ]

Duration of anticoagulant treatment ○ extended ○ temporary

Date of starting the anticoagulant [ ] dd.mm.yyyy

Date of stopping anticoagulant [ ] dd.mm.yyyy

Dose of anticoagulant ○ therapeutic ○ prophylactic

Reason for starting another anticoagulant

- **Atrial fibrillation / atrial flutter with a CHADS2 score of ≥1 or CHA2DS2-VASc ≥2** ☐
- **mechanical heart valve** ☐
- **upper or lower extremity DVT** ☐
- **pulmonary embolism** ☐
- **intraabdominal thrombosis (e.g. portal vein thrombosis)** ☐
- **intracranial vein thrombosis** ☐
- **VTE prophylaxis** ☐
- **bridging for intervention/procedure** ☐
- **other** ☐
- **please specify** [ ]

Discontinuation of study medication during follow-up ○ Yes ○ No

Date of discontinuation [ ] dd.mm.yyyy

<u>Reason for discontinuation:</u>

need for prophylactic anticoagulation ☐

new indication for permanent therapeutic anticoagulation ☐

specify indication:

- **new diagnosis of atrial fibrillation/flutter with a CHADS2 score of ≥1 or CHA2DS2-VASc ≥2** ☐
- **mechanical heart valve** ☐
- **upper or lower extremity DVT** ☐

For peer review only - http://bmjopen.bmj.com/site/about/guidelines.xhtml

Patient ID

pulmonary embolism ☐

intraabdominal thrombosis (e.g. portal vein thrombosis) ☐

intracranial vein thrombosis ☐

other ☐

please specify

bleeding ☐

elective invasive procedure or surgical intervention requiring interruption of anticoagulation ☐

please specify procedure/ intervention

emergency invasive procedure or surgical intervention requiring interruption of anticoagulation ☐

please specify procedure/ intervention

was an emergency anticoagulation reversal done prior to the emergency procedure? ○ Yes ○ No

indicate agent/method used for anticoagulation reversal:

fresh frozen plasma ☐

recombinant factor VIIa concentrate ☐

prothrombin complex [factor II, VII, IX, X] ☐

factor Xa inhibitor-specific antidote [andexanet alpha] ☐

tranexamic acid ☐

invasive or surgical hemostasis procedures) ☐

For peer review only - http://bmjopen.bmj.com/site/about/guidelines.xhtml

Patient ID

new diagnosis of pregnancy	☐
new use of medications interacting with rivaroxaban	☐
strong CYP3A4 inhibitors or inducers	☐
saquinavir	☐
indinavir	☐
ritonavir	☐
nelfinavir	☐
atazanavir	☐
fosamprenavir	☐
tipranavir	☐
darunavir	☐
ketoconazole	☐
itraconazole	☐
voriconazole	☐
posaconazole	☐
rifampicin	☐
rifabutin	☐
rifapentin	☐
phenytoin	☐
phenobarbital	☐
primidone	☐
carbamazepine	☐
St. John's Wort	☐
specify indication	
HIV	☐
fungal infection	☐
epilepsy	☐
depression	☐
other	☐
please specify	

new dual antiplatelet therapy (aspirin plus P2Y12-inhibitor)	☐
Please specify	○ Aspirin plus clopidogrel<br>○ aspirin plus ticagrelor<br>○ Aspirin plus prasugrel<br>○ Other

Discontinuation or Modification of Study Medication

Patient ID

Please specify indication [ ]

new GP IIb/IIIa inhibitors ☐

specify medication
- ○ abciximab
- ○ eptifibatide
- ○ tirofiban

specify indication [ ]

patient withdraws from study participation ☐

Was the discontinuation temporary? ○ Yes ○ No

Date of resuming study medication [ ] dd.mm.yyyy

ew only

For peer review only - http://bmjopen.bmj.com/site/about/guidelines.xhtml

SPIRIT 2013 Checklist: Recommended items to address in a clinical trial protocol and related documents*

Section/item	Item No	Description	Page No
**Administrative information**			
Title	1	Descriptive title identifying the study design, population, interventions, and, if applicable, trial acronym	1
Trial registration	2a	Trial identifier and registry name. If not yet registered, name of intended registry	4
	2b	All items from the World Health Organization Trial Registration Data Set	4
Protocol version	3	Date and version identifier	4
Funding	4	Sources and types of financial, material, and other support	3
Roles and responsibilities	5a	Names, affiliations, and roles of protocol contributors	1, 22
	5b	Name and contact information for the trial sponsor	23
	5c	Role of study sponsor and funders, if any, in study design; collection, management, analysis, and interpretation of data; writing of the report; and the decision to submit the report for publication, including whether they will have ultimate authority over any of these activities	22
	5d	Composition, roles, and responsibilities of the coordinating centre, steering committee, endpoint adjudication committee, data management team, and other individuals or groups overseeing the trial, if applicable (see Item 21a for data monitoring committee)	22-25
**Introduction**			
Background and rationale	6a	Description of research question and justification for undertaking the trial, including summary of relevant studies (published and unpublished) examining benefits and harms for each intervention	6
	6b	Explanation for choice of comparators	6
Objectives	7	Specific objectives or hypotheses	8

For peer review only - http://bmjopen.bmj.com/site/about/guidelines.xhtml

Trial design	8	Description of trial design including type of trial (eg, parallel group, crossover, factorial, single group), allocation ratio, and framework (eg, superiority, equivalence, noninferiority, exploratory)	8
**Methods: Participants, interventions, and outcomes**			
Study setting	9	Description of study settings (eg, community clinic, academic hospital) and list of countries where data will be collected. Reference to where list of study sites can be obtained	8
Eligibility criteria	10	Inclusion and exclusion criteria for participants. If applicable, eligibility criteria for study centres and individuals who will perform the interventions (eg, surgeons, psychotherapists)	9
Interventions	11a	Interventions for each group with sufficient detail to allow replication, including how and when they will be administered	10
	11b	Criteria for discontinuing or modifying allocated interventions for a given trial participant (eg, drug dose change in response to harms, participant request, or improving/worsening disease)	12
	11c	Strategies to improve adherence to intervention protocols, and any procedures for monitoring adherence (eg, drug tablet return, laboratory tests)	11
	11d	Relevant concomitant care and interventions that are permitted or prohibited during the trial	11
Outcomes	12	Primary, secondary, and other outcomes, including the specific measurement variable (eg, systolic blood pressure), analysis metric (eg, change from baseline, final value, time to event), method of aggregation (eg, median, proportion), and time point for each outcome. Explanation of the clinical relevance of chosen efficacy and harm outcomes is strongly recommended	12-14
Participant timeline	13	Time schedule of enrolment, interventions (including any run-ins and washouts), assessments, and visits for participants. A schematic diagram is highly recommended (see Figure)	37
Sample size	14	Estimated number of participants needed to achieve study objectives and how it was determined, including clinical and statistical assumptions supporting any sample size calculations	15-16

Recruitment	15	Strategies for achieving adequate participant enrolment to reach target sample size	10
**Methods: Assignment of interventions (for controlled trials)**			
Allocation:			
Sequence generation	16a	Method of generating the allocation sequence (eg, computer-generated random numbers), and list of any factors for stratification. To reduce predictability of a random sequence, details of any planned restriction (eg, blocking) should be provided in a separate document that is unavailable to those who enrol participants or assign interventions	8
Allocation concealment mechanism	16b	Mechanism of implementing the allocation sequence (eg, central telephone; sequentially numbered, opaque, sealed envelopes), describing any steps to conceal the sequence until interventions are assigned	8
Implementation	16c	Who will generate the allocation sequence, who will enrol participants, and who will assign participants to interventions	8
Blinding (masking)	17a	Who will be blinded after assignment to interventions (eg, trial participants, care providers, outcome assessors, data analysts), and how	8-9
	17b	If blinded, circumstances under which unblinding is permissible, and procedure for revealing a participant's allocated intervention during the trial	12
**Methods: Data collection, management, and analysis**			
Data collection methods	18a	Plans for assessment and collection of outcome, baseline, and other trial data, including any related processes to promote data quality (eg, duplicate measurements, training of assessors) and a description of study instruments (eg, questionnaires, laboratory tests) along with their reliability and validity, if known. Reference to where data collection forms can be found, if not in the protocol	11
	18b	Plans to promote participant retention and complete follow-up, including list of any outcome data to be collected for participants who discontinue or deviate from intervention protocols	15

For peer review only - http://bmjopen.bmj.com/site/about/guidelines.xhtml

Data management	19	Plans for data entry, coding, security, and storage, including any related processes to promote data quality (eg, double data entry; range checks for data values). Reference to where details of data management procedures can be found, if not in the protocol	11
Statistical methods	20a	Statistical methods for analysing primary and secondary outcomes. Reference to where other details of the statistical analysis plan can be found, if not in the protocol	16
	20b	Methods for any additional analyses (eg, subgroup and adjusted analyses)	16-18
	20c	Definition of analysis population relating to protocol non-adherence (eg, as randomised analysis), and any statistical methods to handle missing data (eg, multiple imputation)	16- 18
**Methods: Monitoring**			
Data monitoring	21a	Composition of data monitoring committee (DMC); summary of its role and reporting structure; statement of whether it is independent from the sponsor and competing interests; and reference to where further details about its charter can be found, if not in the protocol. Alternatively, an explanation of why a DMC is not needed	18
	21b	Description of any interim analyses and stopping guidelines, including who will have access to these interim results and make the final decision to terminate the trial	18, 18
Harms	22	Plans for collecting, assessing, reporting, and managing solicited and spontaneously reported adverse events and other unintended effects of trial interventions or trial conduct	(18), 37
Auditing	23	Frequency and procedures for auditing trial conduct, if any, and whether the process will be independent from investigators and the sponsor	11
**Ethics and dissemination**			
Research ethics approval	24	Plans for seeking research ethics committee/institutional review board (REC/IRB) approval	20

Protocol amendments	25	Plans for communicating important protocol modifications (eg, changes to eligibility criteria, outcomes, analyses) to relevant parties (eg, investigators, REC/IRBs, trial participants, trial registries, journals, regulators)	19
Consent or assent	26a	Who will obtain informed consent or assent from potential trial participants or authorised surrogates, and how (see Item 32)	10, 20
	26b	Additional consent provisions for collection and use of participant data and biological specimens in ancillary studies, if applicable	n.a.
Confidentiality	27	How personal information about potential and enrolled participants will be collected, shared, and maintained in order to protect confidentiality before, during, and after the trial	20
Declaration of interests	28	Financial and other competing interests for principal investigators for the overall trial and each study site	22
Access to data	29	Statement of who will have access to the final trial dataset, and disclosure of contractual agreements that limit such access for investigators	19
Ancillary and post-trial care	30	Provisions, if any, for ancillary and post-trial care, and for compensation to those who suffer harm from trial participation	n.a.
Dissemination policy	31a	Plans for investigators and sponsor to communicate trial results to participants, healthcare professionals, the public, and other relevant groups (eg, via publication, reporting in results databases, or other data sharing arrangements), including any publication restrictions	20
	31b	Authorship eligibility guidelines and any intended use of professional writers	(23)
	31c	Plans, if any, for granting public access to the full protocol, participant-level dataset, and statistical code	19
**Appendices**			
Informed consent materials	32	Model consent form and other related documentation given to participants and authorised surrogates	
Biological specimens	33	Plans for collection, laboratory evaluation, and storage of biological specimens for genetic or molecular analysis in the current trial and for future use in ancillary studies, if applicable	n/a

For peer review only

*It is strongly recommended that this checklist be read in conjunction with the SPIRIT 2013 Explanation & Elaboration for important clarification on the items. Amendments to the protocol should be tracked and dated. The SPIRIT checklist is copyrighted by the SPIRIT Group under the Creative Commons "Attribution-NonCommercial-NoDerivs 3.0 Unported" license.

For peer review only
